# Expression of targets of the RNA-binding protein AUF-1 in human airway epithelium indicates its role in cellular senescence and inflammation

**DOI:** 10.3389/fimmu.2023.1192028

**Published:** 2023-07-07

**Authors:** Ilaria Salvato, Luca Ricciardi, Jessica Dal Col, Annunziata Nigro, Giorgio Giurato, Domenico Memoli, Assunta Sellitto, Erwin Pavel Lamparelli, Maria Assunta Crescenzi, Monica Vitale, Alessandro Vatrella, Francesco Nucera, Paola Brun, Federico Caicci, Paola Dama, Thomas Stiff, Leandro Castellano, Sobia Idrees, Matt D. Johansen, Alen Faiz, Peter A. Wark, Philip M. Hansbro, Ian M. Adcock, Gaetano Caramori, Cristiana Stellato

**Affiliations:** ^1^ Department of Medicine, Surgery and Dentistry ‘Scuola Medica Salernitana’, University of Salerno, Salerno, Italy; ^2^ Respiratory Medicine Unit, Department of Biomedical Sciences, Dentistry and Morphological and Functional Imaging (BIOMORF), University of Messina, Messina, Italy; ^3^ Department of Molecular Medicine, University of Padua, Padua, Italy; ^4^ Department of Biology, University of Padua, Padua, Italy; ^5^ Department of Biochemistry and Biomedicine, School of Life Sciences, University of Sussex, Brighton, United Kingdom; ^6^ Centre for Inflammation, Centenary Institute and University of Technology Sydney, Faculty of Science, School of Life Sciences, Sydney, NSW, Australia; ^7^ Immune Health, Hunter Medical Research Institute and The University of Newcastle, Newcastle, NSW, Australia; ^8^ National Heart and Lung Institute, Imperial College London and the National Institute for Health and Care Research (NIHR) Imperial Biomedical Research Centre, London, United Kingdom

**Keywords:** airway epithelium, AU-rich element factor 1 (AUF-1), cell senescence, chronic inflammation, chronic obstructive pulmonary disease, inflammaging, oxidative stress, RNA-binding proteins

## Abstract

**Introduction:**

The RNA-binding protein AU-rich-element factor-1 (AUF-1) participates to posttranscriptional regulation of genes involved in inflammation and cellular senescence, two pathogenic mechanisms of chronic obstructive pulmonary disease (COPD). Decreased AUF-1 expression was described in bronchiolar epithelium of COPD patients versus controls and *in vitro* cytokine- and cigarette smoke-challenged human airway epithelial cells, prompting the identification of epithelial AUF-1-targeted transcripts and function, and investigation on the mechanism of its loss.

**Results:**

RNA immunoprecipitation-sequencing (RIP-Seq) identified, in the human airway epithelial cell line BEAS-2B, 494 AUF-1-bound mRNAs enriched in their 3’-untranslated regions for a Guanine-Cytosine (GC)-rich binding motif. AUF-1 association with selected transcripts and with a synthetic GC-rich motif were validated by biotin pulldown. AUF-1-targets’ steady-state levels were equally affected by partial or near-total AUF-1 loss induced by cytomix (TNFα/IL1β/IFNγ/10 nM each) and siRNA, respectively, with differential transcript decay rates. Cytomix-mediated decrease in AUF-1 levels in BEAS-2B and primary human small-airways epithelium (HSAEC) was replicated by treatment with the senescence- inducer compound etoposide and associated with readouts of cell-cycle arrest, increase in lysosomal damage and senescence-associated secretory phenotype (SASP) factors, and with AUF-1 transfer in extracellular vesicles, detected by transmission electron microscopy and immunoblotting. Extensive *in-silico* and genome ontology analysis found, consistent with AUF-1 functions, enriched RIP-Seq-derived AUF-1-targets in COPD-related pathways involved in inflammation, senescence, gene regulation and also in the public SASP proteome atlas; AUF-1 target signature was also significantly represented in multiple transcriptomic COPD databases generated from primary HSAEC, from lung tissue and from single-cell RNA-sequencing, displaying a predominant downregulation of expression.

**Discussion:**

Loss of intracellular AUF-1 may alter posttranscriptional regulation of targets particularly relevant for protection of genomic integrity and gene regulation, thus concurring to airway epithelial inflammatory responses related to oxidative stress and accelerated aging. Exosomal-associated AUF-1 may in turn preserve bound RNA targets and sustain their function, participating to spreading of inflammation and senescence to neighbouring cells.

## Introduction

RNA-binding proteins (RBPs) participate in posttranscriptional gene regulation (PTGR) by mediating the processing, transport and cytoplasmic fate of mRNAs. Among their multiple tasks, RBPs regulate transcript stability and translation, largely by recognizing *cis*-elements that are mostly present on the 3’-Untranslated Region (3’-UTR) on mRNAs targets and forming ribonucleoprotein complex (mRNPs) with other regulatory proteins and RNA species (miRNA, lncRNAs) (1, 2). Through context-driven interplay in levels and activation of mRNP partners, PTGR actions ultimately determine and adapt the rate of protein output in fundamental processes like cell cycle, proliferation and stress responses (3–7). Alterations of these events, for example through changes in RNP composition favouring aberrant mRNA stabilization and/or increased translation rate, participate to development or persistence of cancer and other pathological conditions (1, 8–11) chiefly through vast regulation of immune and inflammatory responses. These pathogenic events are finely coordinated through regulated mRNA turnover and translation of transcription factors, cytokines, chemokines and other mediators (12–14). In homeostasis and in acute inflammation, mRNA degradation and/or translational repression of these molecules controls the physiologic resolution of inflammatory reactions, preventing an excessive inflammatory response. This role is clearly shown by preclinical evidence and animal models in which altered RBP expression and functions are present in overexpressed immune and inflammatory responses (12, 13, 15, 16). Current understanding of RBP participation in the pathogenesis of human chronic inflammatory diseases is still largely incomplete, yet understanding of their role could uncover novel targeting strategies, as currently investigated for cancer (17–22).

A major non-transmissible chronic disease and leading cause of morbidity and mortality worldwide (23), chronic obstructive pulmonary disease (COPD) displays multiple pathogenic features in which RBP-mediated function may be involved and relevant to disease definition and treatment. There is in fact a lack of effective therapies that reduce disease progression, which results from only a partial understanding of molecular mechanisms underlying disease pathology. COPD is characterized by chronic pulmonary inflammation leading to remodeling of the airways, destruction of the lung parenchyma and pulmonary emphysema and impaired lung function (24, 25). Chronic inflammation in the lungs of COPD patients is triggered, on a complex background of genetic and epigenetic factors, by chronic exposure to environmental noxious stimuli, chiefly cigarette smoke (CS). The ensuing oxidative stress in the airways and lung drives DNA damage and accelerated cellular senescence, characterized by cell cycle arrest and continued metabolic activity (26). These changes trigger a cellular Senescence-Associated Secretory Phenotype (SASP), where transcriptomic and epigenetic changes drive over-expression of cytokines, chemokines, growth factors and many mediators, altering the local tissue response and spreading the effects of SASP mediators through increase in macrovesicles and exosome generation (27–29). A large-scale proteomic analysis of SASP profiles in different human cell types confirmed the enrichment for protein markers of senescence (30).

Indeed, RBPs are key determinants of oxidative stress-mediated inflammatory response and cellular senescence (31–34). In particular, they mediate PTGR of numerous SASP mediators, including interleukin (IL)-6 and IL-1β, chemokine (C-X-C motif) ligand 1 (CXCL8 or IL-8) and chemokine (C-C motif) ligand 2 (CCL2), transforming growth factor-β (TGF- β) and others (35–39). Recent evidence show that RBPs are also present in extracellular vesicles (EVs) and could mediate the transfer of mRNAs and miRNA into other cells (40, 41).

The functional profile of the RBP AUF-1 is of particular relevance in this setting. AUF-1, encoded by the Heteronuclear Ribonucleoprotein D (*HNRNPD*) gene belongs to a family of ubiquitously expressed proteins, which chiefly promote the decay of mRNA targets (8, 42). Mouse models of AUF-1 deficiency (^-/-^) indicate its critical involvement in both inflammatory responses and in mechanisms of cell senescence. *Auf1^-^/^-^* mice are highly susceptible to endotoxin-induced septic shock with increased mortality due to exaggerated inflammatory responses, mediated by the lack of AUF-1- mediated degradation of the inflammatory cytokines tumor necrosis factor (TNF)-α and IL-1β mRNA (43). In these animals, *Auf1^-/-^* T cells and macrophages have increased expression of IL-2, TNF -α, and IL-1β (44). The mice have early-onset aging with increased telomere erosion and accelerated cellular senescence. This phenotype results from complex mechanisms, as AUF-1 acts as transcriptional regulator of the telomere subunit TERT but also exerts post-transcriptional control by destabilizing cell-cycle checkpoint mRNAs, such as cyclin-dependent kinase inhibitors p21^WAP/CIP1^ (45) and p16 (46). Large-scale *in vitro* deconvolution of basic molecular determinants of AUF-1 function confirmed its complex control in cell senescence and mechanisms of DNA repair (47).

We reported a selective loss of AUF-1 expression in bronchiolar epithelium of COPD patients versus matched control subjects and in the airway epithelial cell line BEAS-2B stimulated with cigarette smoke extract (CSE) and cytomix (48). This finding was specific for the epithelium, as neither other structural cells (endothelium, fibroblasts) or immune cells (macrophages, infiltrating leukocytes) displayed this difference, nor the levels of the RNA binding proteins Tristetraprolin (TTP) and HuR were changed in the *ex vivo* and *in vitro* models (48). We again documented changes in AUF-1 expression in COPD *in silico* within the identification of a global downregulation for a curated list of 600 RBPs (49) in two COPD bronchiolar epithelium transcriptomic databases (50). The downregulated RBP expression pattern was significantly represented for several pathogenic COPD pathways by Genome Ontology (GO) analysis, expanding relevance of RBP biology in chronic lung inflammation beyond a single member.

These data prompted the investigation of AUF-1 mRNA targets in airway epithelium and of the relevance of AUF-1-dependent functions, along with the mechanisms of decreased AUF-1 levels found in this experimental model. AUF-1-associated transcripts were identified by RIP-Seq, validated to confirm AUF-1 association and analysed for shared binding motifs. We then investigated the effect of AUF-1 loss (upon cytokine challenge or siRNA-mediated silencing) on the expression profile and stability of its mRNA targets and its role in inducing markers of cell senescence. Investigation of mechanisms mediating cytomix-induced AUF-1 loss led to the identification of induced transfer of AUF-1 into EVs released by airway epithelial cells. GO and *in silico* analyses of multiple COPD transcriptomic datasets validated the findings showing significant changes in RIP-Seq-identified AUF-1 targets in human disease, indicating their participation in pathways mutually relevant for AUF-1 regulation and COPD pathogenesis.

Collectively, we show that experimental conditions of inflammation and cellular senescence in airway epithelial cells lead to loss of intracellular AUF-1 and its transfer in exosomes, possibly involving this RBP in the spreading of inflammation and senescence, by acting as cargo for bound transcripts to exosomes. Changes in AUF-1 target expression in multiple epithelial COPD transcriptomic profiles - from lung tissue, bronchiolar epithelium and single-cell sequencing databases - are consistent with its relevant participation to epithelial responses during chronic inflammation and underscore the need of further knowledge on AUF-1 and general RBP-mediated gene regulation in human inflammatory diseases.

## Methods

Study materials and commercial sources are listed in **Table S1**.

### Cell culture and treatments

The SV40-immortalized human bronchial epithelial cell line BEAS-2B (ATCC) was cultured in DMEM/Ham’s F12 (EuroClone) supplemented with 5% heat-inactivated FBS (EuroClone), 2 mM L-glutamine (Lonza), penicillin (100 U/ml)-streptomycin (100 mg/ml) (Lonza) and 0.2% fungizone (EuroClone) (37). Human small airway epithelial cells (HSAECs, PCS-301-010, ATCC) were cultured as submerged monolayers in Airway Epithelial Cell Basal Medium (PCS-300-030, ATCC) supplemented with Bronchial Epithelial Growth Kit (PCS-300-040, ATCC). Both cell lines were incubated at 37°C, 5% CO_2_. For challenge, when reaching 70% confluency cells were kept in medium only or stimulated using cytomix (10 nM each rHuIL-1β, TNFα, IFN-γ, GoldBio) for 48 h. For exogenous AUF-1 silencing, BEAS-2B cells were transfected when reaching 50%–60% confluency using the non-liposomal cationic vehicle FuGENE HD (Promega) according to the manufacturer’s instructions, using 100 nM AUF-1 siRNA (5′-AAGAUCCUAUCACAGGGCGATdTdT-3′) (47) or a scrambled control siRNA (5′-GAGUCAACCUUAUGAUACUdTdT-3′). After 48 h, cells were exposed to cytomix or medium for additional 48 h prior to cell harvesting. For mRNA stability assays, resting and cytomix-treated cells were either harvested at 48 h (time 0), for analysis of steady state levels or after 1, 2 and 4 h of culture with the transcriptional inhibitor actinomycin D (3 µg/ml ActD, Sigma) (51). For proteasome inhibition experiments, cells were incubated for 2h with 10 μM of MG-132 compound (Sigma), then medium was replaced and cells were treated with cytomix or medium for additional 48 h prior to cell harvesting. In all experiments, cells were harvested using trypsin/EDTA (Lonza), counted and viability was verified by Trypan Blue exclusion (EuroClone). Cell viability was ≥ 90% at harvest in all conditions.

### RNA immunoprecipitation and sequencing assay

RIP is an antibody (Ab)-based technique developed to study the interaction between a RBP and its endogenous targets (52, 53), performed according to established protocols for BEAS-2B (37, 38). Cytosolic fractions were extracted after lysing BEAS-2B cells (n=3, 10^8^ cells/condition) with polysome lysis buffer (10 mM HEPES pH 7.0, 100 mM KCl, 5 mM MgCl_2_, 0.5% NP40, 1 mM DTT, 100 U/ml RNase out, 400 μM Vanadyl-Ribonucleoside Complex, 1x Protease Inhibitors) (52). An aliquot of cytosolic extract (10%) was taken as Input. For IP with, 2 mg of cytosolic extract were incubated (4°C, overnight) with 4 μg of anti-AUF-1 Ab (HPA004911, Atlas Antibodies) and for control IP, IgG isotype (02-6102, Thermo Fisher Scientific). Then, 100 μl of pre-blocked magnetic beads (Dynabeads, Thermofisher) were added and the incubation was continued (4°C, 4 h). Total RNA pools bound to AUF-1/control Ab were extracted by adding TriFast reagent (EuroClone) directly to the washed beads, following the manufacturer’s instructions. The size distribution of each RNA sample was assessed by running a 1 μl aliquot on an Agilent High Sensitivity RNA chip using an Agilent Technologies 2100 Bioanalyzer (Agilent Technologies). The RNA concentration of each sample was determined using a Quant-IT RNA Assay Kit-High Sensitivity and a Qubit Fluorometer (Life Technologies).Total RNA was used to prepare sequencing libraries (54). Briefly, 1 μg of RNA Input and 300 ng of AUF-1- and Ctrl Ab-IP RNA were used as the starting material for sequencing library preparation from three independent experiments. Immunoprecipitated RNAs were fragmented and converted to cDNA after adaptor ligations in preparation for sequencing. Principal Component Analysis (PCA) indicated that one of the biological replicates was discordant respect to the other two samples; therefore, two of the three original experiments were considered for further analysis. After normalization, a total of 12,727 transcripts were expressed in the cell line Input samples. Indexed triplicate libraries were prepared with a TruSeq Stranded Total RNA (Illumina Inc.). The quality of the libraries was evaluated with 2100 Bioanalyzer (Agilent) and Qubit dsDNA HS Assay Kits (Thermo Fisher Scientific). Libraries were sequenced at 3 pM/lane (paired-end, 2 × 75 cycles) on a NextSeq 500 (Illumina Inc.). Bioinformatic analysis was performed. as described (54). An average of 27,466,311, 36,765,708 and 25,288,124 reads were obtained from the Input, AUF-1 IP and IgG IP libraries, respectively. Despite differences in total read numbers due to the low amount of Input cDNA, the IPs consistently yielded many more mappable reads than the controls. The quality of the sequenced reads was assessed by evaluating the quality score, presence of k-mers and balance of the GC percentage, using FastQC software (55). Cutadapt software was used to remove the adapter sequences (56) using default parameters. The human transcriptome and genome (assembly hg38) was used as a reference for the alignment, which was performed using STAR version 2.7 (default parameters) (57).

### RIP-Seq data analysis

Feature-count was used with default parameter to compute gene-level read counts (58). Only the genes whose read count was ≥ 10 in at least one sample were considered for the further analysis. The R bioconductor package DESeq2 was used to test the differential expression of genes from RIP-Seq data compared to controls (59). RNAs showing Enrichment Factors (EFs) ≥1.5 and False Discovery Rates (FDRs) ≤0.05 computed according to Benjamini–Hochberg were considered for further analysis. Transcript per million (TPM) was computed using RSEM (60). Scatter plot and box plot were elaborated with R (v3.6.2) (61). Raw Rip-sequencing data are deposited in the EBIArrayExpress database with accession number E-MTAB-12583.

### Prediction of binding motifs

The p45^AUF-1^ sequence from NCBI was used for analysis with the CatRAPID algorithm (62). The list of coding and non-coding targets of AUF-1 protein was filtered considering a Discriminative Power (DP) ≥0.75. Enrichment ratios for every transcript in each RIP-Seq experiment were log transformed. Graphic visualization was elaborated with R version 3.6.2. Prediction of binding motifs for AUF-1 were identified on 3’UTR sequences of experimental targets with Sequence & Structure Motif enrichment Analysis for Ranked RNA data generated from *in vivo* binding experiments (SMARTIV) (63, 64), with standard setting and Multiple Expectation maximizations for Motif Elicitation (MEME version 5.4.1) (65), a position weight matrix-based tool for motif identification, using the following parameters: number of repetitions, any; minimum width for each motif: 5; maximum width for each motif: 35; and maximum number of motifs to be found: 20. Motifs with E-value ≤ 0.05 according to minimum hypergeometric statistical approach (mmHG) were considered significant.

### Biotin pulldown assay

Biotinylated 3’UTRs were generated by PCR of BEAS-2B RNA (**Table S2**). Long 3’UTRs were fragmented in adjacent sequences to allow correct *in vitro* transcription and biotinylation. PCR products were purified from agarose gels and used as templates for biotinylated RNAs synthesis using MAXIscript™ T7 Kits (AM1312, Invitrogen) and Biotin-11-cytidine-5’-triphosphate (ENZ-42818, Enzolife). Unstimulated BEAS-2B cells were lysed with polysomal extraction buffer (100 mM KCl, 5 mM MgCl_2_, 10 mM Hepes pH 7.0, 0.5% NP-40, 1X protease inhibitor) to obtain cytoplasmic fractions. Cytosolic lysates (500 µg) were incubated with 1 µg of biotinylated transcripts (30 min) and, then, ribonucleoprotein complexes were isolated with streptavidin-conjugated Dynabeads (11205D, Invitrogen). The presence of AUF-1 in the pulldown material was verified by immunoblot analysis (38, 66).

### Protein extraction and immunoblot

Proteins were separated, quantified, and subjected to Western blot analysis as described (48). For total protein extraction, cells were directly lysed in buffer containing 50 mM Tris/HCl at pH 7.5, 150 mM NaCl, 2 mM EDTA, 2 mM EGTA, 25 mM NaF, 25 mM β-glycerolphosphate, 0.1 mM Na3VO4, 0.1 mM PMSF, 0.2% Triton X-100, 0.3% NP40, and a cocktail of protease inhibitors (100 X, EuroClone). After incubation (4°C, 30 min) the lysates were centrifuged (15,700 x*g*, 4°C, 15 min). A total of 15 µg of protein per well was separated using 4–15% sodium dodecyl sulfate-polyacrylamide gel electrophoresis and transferred to nitrocellulose membranes. Membranes were then blocked with 5% milk-TBS-Tween buffer (TBS plus 0.1% Tween-20, room temperature, 1h) and incubated with primary antibodies at 4°C overnight, washed with TBS-Tween buffer three times and incubated with corresponding horseradish peroxidase-conjugated secondary antibodies (room temperature, 45 min). Signals were detected using the “Pierce ECL Western Blotting Substrate” method (Thermo Fisher Scientific) and analysed using ImageLab software with the Chemidoc image acquisition and analysis tool (BioRad).

The following primary antibodies were used: anti-AUF-1 (HPA004911, Atlas), anti- Human antigen R (HuR) (sc-5261, Santa Cruz), anti- Tristetraprolin (TTP) (ab83579, Abcam), anti-p53 (sc-126, Santa Cruz), from Cell Signaling Technology, anti-phospho-Rb (9308), anti-p21 (2947), anti-phospho-p53 (9286), anti- β-actin (3700), anti-β-tubulin (9F3), anti-CD9 (10626D); anti-CD63 (10628D, Invitrogen, used in non-reducing conditions).

### RNA extraction, cDNA synthesis and quantitative real-time PCR

Total RNA was extracted using TriFast reagent (EuroClone) and reverse transcription was achieved using LunaScript^®^ RT SuperMix Kits (New England Biolabs) following the manufacturer’s protocol. Template complementary DNA (cDNA) was subjected to qRT-PCR using FluoCycle II SYBR Master Mix (Euroclone) according to the manufacturer’s protocol (48). Primers were published or designed with Primer-BLAST software https://www.ncbi.nlm.nih.gov/tools/primer-blast/ (**Table S2**). Reactions were run in duplicate on a LightCycler 480 II (Roche), using the following setup: denaturation at 95°C for 5 minutes; amplification at 95°C for 15 seconds, and 60°C for 60 second (45 cycles). Target expression was normalized to GAPDH by the cycle threshold (Ct) method and expressed using the 2^−ΔΔCt^ calculation as fold over control.

### Cellular senescence assays

Cells were grown in submerged cultures in presence of: media only; cytomix, for 48h: etoposide (6 µM) for 24 h, used as positive control for senescence. The culture media was replaced with no further stimulation (as post-challenge time 0) and cells were cultured at 37°C for an additional 5 days (as post-challenge time 5). Cellular senescence was then detected using flow cytometric analysis of β-D-galactopyranoside, a fluorogenic β-galactosidase substrate, using Cell Meter™ Cellular Senescence Activity Assay Kits (23005, AAT Bioquest) according to the manufacturer’s protocol (67). Fluorescence was detected using a FACSVerse™ flow cytometer (BD Biosciences), in the FITC channel, and the data were analyzed using BD FACSuite™ software.

### Analysis of SASP cytokines

BEAS-2B and HSAEC supernatants were screened for inflammatory cytokines associated with SASP (IL-1β, MCP-1, IL-6, IL-8) using bead-based immunoassays LEGENDplex™ (740809, Biolegend), following the manufacturer’s protocol. Briefly, cell supernatants were incubated for 2 h with two sets of specific anti-cytokine antibodies-conjugated beads, differentiated by size and internal fluorescence intensities, followed by a 1 h incubation with a detection antibody. After 30 min incubation of the mix with streptavidin-conjugated phycoerythrin, the fluorescent signal intensity, which is proportional to the amount of bound analytes, was detected using a FACSVerse™ flow cytometer (BD Biosciences) and data were analyzed with the LEGENDplex Data Analysis Software.

### EVs isolation

BEAS-2B and HSAEC cells were seeded with equivalent cell numbers between conditions (40 x10^6^ cells/condition) and cultured with corresponding EV-depleted culture mediums, obtained by overnight centrifugation (100,000 x*g*). Cell-derived EVs were isolated from culture media of unstimulated and cytomix-stimulated cells by differential centrifugation following an established protocol (68, 69). Briefly, cell supernatants were centrifuged at 300 x*g* for 10 min to pellet cells, then at 2,000 x*g* for 10 min to pellet dead cells and 10,000 x*g* for 30 min to remove cell debris. Finally, supernatants were ultra-centrifuged (100,000 x*g*, 70 min) using a Beckman Coulter Optima XE-100 Ultracentrifuge with SW 32.1 swinging-rotor. EV pellets were resuspended in 50 µl of PBS.

### Dynamic light scattering analysis and nanoparticle tracking analysis

DLS analyses the velocity distribution of particle movement caused by Brownian motion by measuring fluctuations of scattered light intensity. Then, the particle size is calculated size *via* the Stokes-Einstein equation (70). 10 µL of EVs were diluted in 990 µL of water and size was measured using a Nano ZS Malvern Zeta Sizer (model 1000HSa, UK, 25°C), equipped with a He-Ne laser of 633 nm and detector angle of 173°C. Analyses were performed in three independent technical replicates for each sample. EV size was expressed as mean ± Standard deviation (SD).

The NTA determines the concentration and size of particles in EV samples through identification and tracking of individual nanoparticle movements under Brownian motion. In detail, 10 µL of EVs suspension was diluted in 1000 µL of water and then injected into the Nanosight NS300 (Malven) for measurement. EVs size and concentration were expressed as mean ± SEM of nm and number of particles/ml, respectively.

### Transmission electron microscopy

One drop of sample solution (~25µl) was placed on 400 mesh holey film grids. After staining with 2% uranyl acetate (2 min) samples were observed with a Tecnai G2 (FEI) transmission electron microscope operating at 100 kV. Images were captured with a Veleta (Olympus Soft Imaging System) digital camera. For cell monolayer samples, seeded cells were washed in 1x HBSS and fixed in 2.5% glutaraldehyde (Sigma-Aldrich) in 0.1M Hepes buffer (4 °C, 1 h, pH 7.4). After three water washes, samples were dehydrated in a graded ethanol series and embedded in epoxy resin (Sigma-Aldrich). Ultrathin sections (60-70 nm) were obtained with an Ultrotome V (LKB) ultra-microtome, counterstained with uranyl acetate and lead citrate and viewed with a Tecnai G2 (FEI) transmission electron microscope. Images were captured with a Veleta (Olympus Soft Imaging System) digital camera. The mean ± SD EVs size was calculated using ImageJ software https://imagej.nih.gov/ on 100 particles chosen randomly in pictures from control and cytomix-treated samples.

### Immunogold

A drop of sample solution (~25 µl) was placed on a 400 mesh holey film grid for 2-3 min. Subsequently they were incubated with blocking solution (0.5% bovine serum albumin (BSA), in PBS, room temperature, 30 min). Immediately the grids were then incubated (30 min, RT) with a primary antibody anti-AUF-1 (HPA004911, Atlas) diluted 1:40 in blocking solution and then washed three times with PBS (5 min each, RT). The grids were then incubated with an IgG Gold II secondary anti-rabbit antibody coupled to gold particles (5nm, Sigma Aldrich G3779, RT, 30 min). After washing, in PBS (3X) and water (2X), grids were counterstained with uranyl acetate and lead citrate and viewed with a Tecnai G^2^ (FEI) transmission electron microscope. Images were captured with a Veleta (Olympus Soft Imaging System) digital camera.

### GO and pathway analyses

GO analysis was performed with Ingenuity Pathway Analysis (IPA) software (71). Heatmaps and Pearson correlation matrices for correlated expression changes were generated using tMEV. GOPlot was used to visualize the Circos plot (72, 73).

### Donors providing lung biopsy samples for RNA sequencing

Bronchial rings and peripheral lung samples were obtained from subjects recruited from the Respiratory Unit of the University Hospital of Messina, Italy, among patients undergoing lung resection for peripheral lung carcinoma (**Table S3**). Smokers with mild-to-moderate stable COPD (n=7) were compared with age- and smoke history-matched smokers with normal lung function (NLF, n=5). Diagnosis of COPD was defined according to international guidelines as the presence of post-bronchodilator forced expiratory volume in 1 s (FEV_1_)/forced vital capacity (FVC) ratio <70% or the presence of cough and sputum production for at least 3 months in each of two consecutive years (24, 74). All patients were in a stable condition at the time of the surgery and had not suffered acute exacerbations or upper respiratory tract infections in the preceding two months. None had received glucocorticoids or antibiotics within the month preceding surgery, or inhaled bronchodilators within the previous 48 h. Patients had no history of asthma or other allergic diseases. All former smokers had stopped smoking for >1 year. Each patient underwent medical history collection, physical examination, chest radiography, electrocardiogram, routine blood tests, and pulmonary function tests during the week prior to surgery. Pulmonary function tests (Biomedin Spirometer, Padova, Italy) were performed as described (75) according to published guidelines. The study was approved by the local Ethics Committees of the University Hospitals of Messina and participating patients and control subjects signed the approved informed consent forms.

### RNA-seq of human lung biopsies

For gene expression analysis libraries were prepared with the Lexogen QuantSeq 3′ mRNA-Seq Library Prep Kit (FWD) for Illumina (cat. no. 015.96), as per the manufacturer’s instructions. The modified protocol for FFPE samples was used and RNA input was 250ng. qPCR was performed to find the optimal cycle number for endpoint PCRs, using the PCR Add-on Kit for Illumina (cat. no. 020.96) to quantify cDNA before final library amplification. Library pooling was performed by BGI and NGS was run on their DNBSEQ platform (BGI Genomics, HK) with PE100 reads. Read count was performed on the BlueBee platform using standard settings for the Quantseq 3’ kit (www.lexogen.bluebee.com). Differential expression analysis was performed using DESeq2 package from the Bioconductor (https://bioconductor.org/packages/release/bioc/html/DESeq2.html). Heatmaps were generated using tMEV tools v4_9_0.45. (76, 77).

### Gene set variation analysis

This statistical method evaluates variations in underlying mechanisms between groups (78) and was used to compute the Enrichment Score (ES) of the AUF-1 RIP-seq gene set in all subjects included in the database GSE5058 (79, 80). The analysis was performed according to described parameters (81). Gene signatures were considered significantly differentially expressed with differences in Ess (dES) ≥ 0.2 between the groups and p-value <0.05.

### Single cell RNA-sequencing of differentiated primary broncho-epithelial cells

AUF1 target expression was evaluated in scRNAseq datasets generated from primary broncho-epithelial cells from stable COPD patients and healthy control subjects differentiated at the air-liquid interface (ALI) (82).

### Statistical analysis

For RIP-seq data statistical analysis, FDR ≤ 0.05 computed according to Benjamini–Hochberg were considered for further analysis. Data from immunoblot densitometry and qRT-PCR were analyzed using Student’s paired *t*-test. For cellular senescence activity assays, ANOVA test with FDR *post hoc* multiple comparison analysis was performed. Statistical analysis was performed using GraphPad Prism 5 (*GraphPad Software Inc.*). A probability *p* ≤ 0.05 was considered significant.

## Results

### Identification of AUF-1-associated transcripts in BEAS-2B cells by RIP-Seq analysis

**Figure 1:**
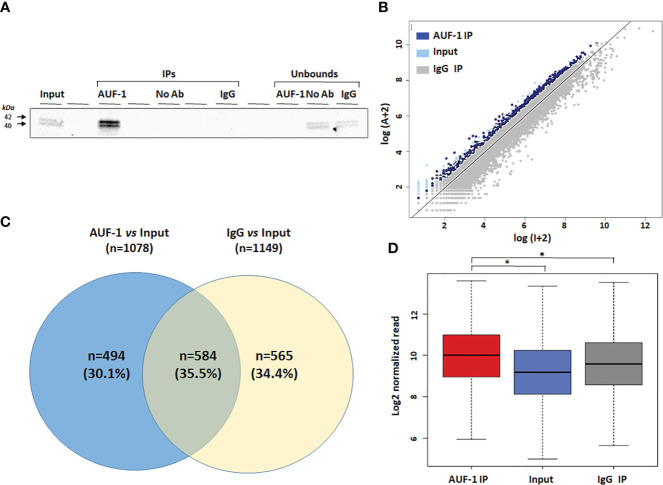
Transcripts associated with AUF-1 in unstimulated BEAS-2B cells identified by RIP-Seq. **(A)** Representative immunoblot analysis (n=3) showing selective AUF-1 IP compared to unbound controls. The Input samples (Input) were incubated with both AUF-1 and IgG antibodies (AUF-1 IP and IgG IP, respectively) and then immunoprecipitated with magnetic beads. An additional antibody-free control sample (no Ab) was performed. **(B)** Scatter plot of RIP-Seq data. Read counts for AUF-1 IP, IgG and Input controls were normalized and log-transformed. Dark blue and light blue dots represent enriched AUF-1 IP and IgG targets (EF≥1.5 and FDR ≤ 0.05), respectively. Gray dots represent background (Input). Axes represent log_2_ read count in Input (X) and AUF-1 IP (Y). **(C)** Venn diagram showing exclusive and overlapping targets between AUF-1 IP- and IgG-IP-enriched transcripts (each total number in parenthesis) vs Input (EF ≥ 1.5 and FDR ≤ 0.05). **(D)** Boxplot showing the enrichment of the 494 AUF-1 transcript targets in Input, AUF-1 IP and IgG IP samples. Y axis represents the log_2_ of the normalized read count. *p ≤ 0.05 (Student’s t-test).

Cytosolic extracts of unstimulated BEAS-2B cells (n=3) were isolated and subjected to RIP-Seq analysis (see Methods). Immunoblot analysis of the protein fraction (**Figure 1A**) revealed a high level of enrichment in IP AUF-1 compared to IP IgG, No-Ab IP and unbound fraction controls. To visualize the enrichment data, each sequenced transcript in the IP sample *vs* the Input samples were plotted. A scatter plot was constructed using the log-transformed and normalized read numbers (**Figure 1B**). Enrichment analysis was set with EF ratio in AUF-1 IP vs Input and IgG IP vs Input at ≥ 1.5 and FDR ≤ 0.05. With these cutoff values, 1,078 transcripts were significantly immunoprecipitated in AUF-1 IP *vs* Input and 1,149 transcripts in IgG IP *vs* Input samples. Subsequently, the two datasets were crossed and overlapping targets with IgG IP *vs* Input transcripts were excluded. As a result, 494 RIP-Seq-identified AUF-1 targets were considered for further analysis (**Figure 1C**), a number in line with previous RIP-based transcriptomic studies in these cells (38). **Table 1** lists the top 20 RIP-Seq-identified AUF-1 targeted transcripts ranked by enrichment value (full target list in **Table S4**). For these selected targets, the average of reads enrichment was significantly higher in AUF-1 IP compared to the Input and to IgG IP (median normalized reads: 1,024, 583 and 762 in AUF-1 IP, Input and IgG IP samples, respectively, **Figure 1D**). Of relevance, the AUF-1 IP-target pool includes transcripts previously identified as associated to AUF-1, such as DICER (109) and ZFP36L1 (110) and almost all targets (n= 490) were listed among the over 2,000 mRNAs identified as bearing AUF-1 binding sites in a model of AUF-1-overexpression in human embryonic kidney cells by the more stringent PAR-CLIP analysis (47).

**Table 1:** Top 20 AUF-1 target genes ranked according to enrichment factor (AUF-1 IP vs Input) in RIP-Seq experiments.

Gene symbol	Full Name	EF	FDR	Main Functions	References
**PRR36**	Proline Rich 36	5,73	0,020203	Unknown function	(83)
**GLIS2**	GLIS Family Zinc Finger 2	4,95	0,00745	Transcription factor	(84, 85)
**ZNF385A**	Zinc Finger Protein 385A	4,4	5,15E-05	Zinc finger protein	(86)
**TCF7L1**	Transcription Factor 7 Like 1	4,14	0,003217	Wnt signaling pathway	(87–89)
**PIANP**	PILR Alpha Associated Neural Protein	3,52	0,000871	Ligand for the paired Ig-like type 2 receptor alpha	(90)
**MBD6**	Methyl-CpG Binding Domain Protein 6	3,42	2,49E-09	Binds to heterochromatin	(91)
**MUC1**	Mucin 1, Cell Surface Associated	3,37	1,81E-06	Binds to oligosaccharides by the extracellular domain	(92, 93)
**FOXP4**	Forkhead Box P4	3,27	7,46E-05	Transcriptor factor	(94, 95)
**KDM6B**	Lysine Demethylase 6B	3,2	0,000139	Lysine-specific demethylase	(96, 97)
**FBRSL1**	Fibrosin Like 1	3,09	0,000336	Unknown function	(98)
**C1orf226**	Chromosome 1 Open Reading Frame 226	2,86	0,039358	Unknown function	(99)
**AP001972.5**	AP001972.5	2,84	0,032884	Unknown function	
**STX1B**	Syntaxin 1B	2,83	0,012534	Mediator of calcium-dependent synaptic vesicle release	(100)
**CRTC1**	CREB regulated transcription coactivator 1	2,73	0,001314	Co-activator of the transcription factor CREB	(101)
**AL513165.1**	AL513165.1	2,66	0,034496	Unknown function	
**ATXN2L**	Ataxin 2 Like	2,6	1,37E-08	Regulator of stress granules	(102)
**RNF44**	Ring Finger Protein 44	2,6	0,000146	E3 ligase	(103)
**IL17RD**	Interleukin-17 Receptor D	2,58	0,026678	Orphan receptor member of the IL-17R family	(104, 105)
**KIAA1522**	KIAA1522	2,58	1,23E-08	Unknown function	(106)
**HIVEP3**	Human Immunodeficiency Virus Type 1 Enhancer-Binding Protein 3	2,47	0,000702	Transcription factor	(107, 108)

Full list (n=494) in **Supplementary Table S4**. EF, Enrichment Factor; FDR, False Discovery Rate.

### Identification of predicted binding motifs in RIP-Seq-identified AUF-1 targets; AUF-1 association to 3’UTR regions of selected transcripts by biotin pull-down

**Figure 2:**
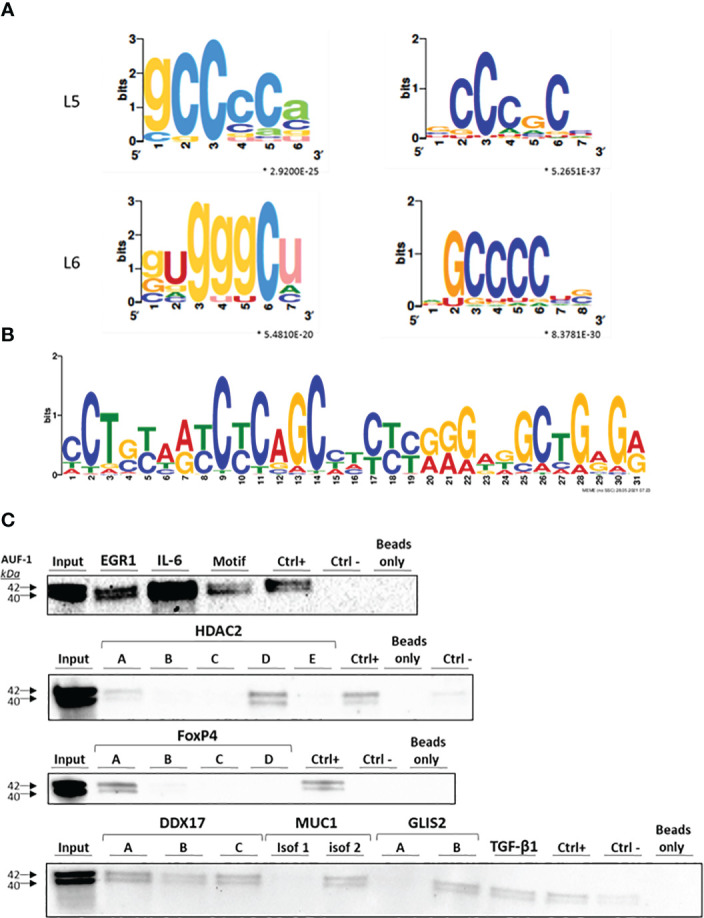
Identification of predicted binding motifs in AUF-1-associated transcripts. **(A)** *k*-mer length 5 (L5) and 6 (L6) graphic generated using the SMARTIV tool representing the probability matrix of the AUF-1 motif, showing the relative frequency of each nucleotide for each position within the motif sequence. The motif originated from the 3’UTR sequences of the exclusive n= 494 AUF-1 transcripts (**Figure 1C**) obtained from the RIP-Seq study. Upper and lower case letters show the secondary structure prediction (A, G, C, U for unpaired nucleotides and a,g,c,u for paired nucleotides). *p-value ≤ 0.05 according to minimum hypergeometric statistical approach. **(B)** Graphic of the 30-mer consensus AUF-1-binding motif generated using the MEME tool, identified within the top 1,000 peaks originating from the 3’UTR sequences of RIP-Seq – derived AUF-1 targets (full list of consensus motifs in **Figure S2**). This motif was utilized to generate the biotinylated probe for validation with biotin pull-down reported in panel C (listed in **Table 2**). **(C)** Representative immunoblots (n=2) of AUF-1 detection by RNA biotin pulldown in BEAS-2B cytoplasmic lysates using biotinylated 3’UTR probes for the indicated AUF-1 targets and the *in vitro* biotinylated AUF-1 GC-rich motif, synthesized from consensus sequence shown in panel B, selecting highest-frequency nucleotides. Capital letters (A to E) represent biotinylated fragments of adjacent sequences used for long 3’UTRs; “CTRL+”, positive control (Cyclin D1 3’UTR); “CTRL-”, negative control (PD-L1 coding sequence).

The interaction of AUF-1 with its target mRNAs is mediated predominantly by motifs located in the 3′UTR of the transcripts (39, 47, 111, 112). Thus, we focused our analysis of enriched elements to the 3’UTR of the AUF-1-bound targets. We screened the 494 epithelial AUF-1 targets for the occurrence of 3’UTR motifs using the SMARTIV tool (63, 64). **Figure 2A** shows 4 core motifs with k-mer length of 5 and 6 nucleotides. Within the experimental dataset 12 enriched gapped k-mer motifs were further identified, mostly comprising Guanine-Cytosine (GC) nucleotides, which had the highest frequency of hits over the entire SMARTIV database (**Figure S1A**). We also searched for an extended 30-mer core motif using a MEME Suite tool. This analysis confirmed that the majority of experimental AUF-1 epithelial targets shared a GC-rich motif. We selected for validation the motif displaying the most significant E-value (see Methods) (**Figure 2B** for validated motif; remaining are shown in **Figure S1B**).

To validate AUF-1 association with targets identified by RIP-Seq analysis, biotin pull-down experiments were set for selected transcripts, chosen to represent the spectrum of EF (**Figure 2C**). Cytoplasmic lysates from unstimulated BEAS-2B cells (n=2) were incubated with biotin-labelled synthetic RNAs corresponding to full-length or segments of targets’ 3’UTRs, in case the 3’UTR was too long for synthesis of a single biotin-labelled molecule (**Table 2**). IL-6 mRNA was included since it is an important mediator of SASP also known to be an ARE-bearing gene whose mRNA decay is accelerated by AUF-1 binding (44, 113, 114).

**Table 2:** AUF-1-bound mRNAs, with relative EF (AUF-1 IP vs Input), selected for biotin pull-down validation.

Gene symbol	Full name	EF	3’UTR pulled-down fragment (Table S2)
**GLIS2**	GLIS Family Zinc Finger 2	4.95	B
**MUC1**	Mucin 1, Cell Surface Associated	3.37	Isoform 1
**FoxP4**	Forkhead Box P4	3.27	A
**TGF-β1**	Transforming growth factor beta 1	2.16	Full length
**EGR1**	Early growth response 1	2.15	Full length
**DDX17**	DEAD-Box helicase 17	2.13	A, B, C
**HDAC2**	Histone deacetylase 2	1.7	A, D
**IL-6**	Interleukin-6	As control	Full length
**GC-rich motif**			CCTGTAATCTCAGCCTCCTGGGAGGCTGAGA

For the newly identified GC-rich motif selected for validation, a synthesized biotinylated sequence containing the nucleotides with highest frequency was utilized. The 3’UTR of Cyclin D1, a known target of AUF-1 (115) and a beads-only sample and a non-AUF-1 target sequence (PD-L1 coding sequence) were included as positive and negative controls, respectively. Immunoblot analysis (**Figure 2C**) revealed the presence of AUF-1 in the starting lysate (Input) and in the pulldown fractions obtained with biotinylated 3’UTR sequences of all experimental transcripts. In particular, AUF-1 was detected in the pulldown fraction obtained with full-length 3’UTRs (EGR1, TGF-β1, isoform 1 of MUC1 and IL-6) and specific segments for other transcripts: segment A and, more abundantly, with segment D of HDAC2 3’UTR, segment A of FoxP4-3’UTR, all segment of DDX17-3’UTR, segment B of GLIS2-3’UTR. Importantly, AUF-1 was detected in association with the sequence modelled on the motif shown in **Figure 2B**. These results support the data obtained from RIP-Seq analysis, confirming the association of AUF-1 to the 3’UTR of the selected transcripts, pointing in some cases to specific regions of 3’UTRs.

### Effect of AUF-1 loss on steady state and stability of selected AUF-1 targeted transcripts in BEAS-2B cells

**Figure 3:**
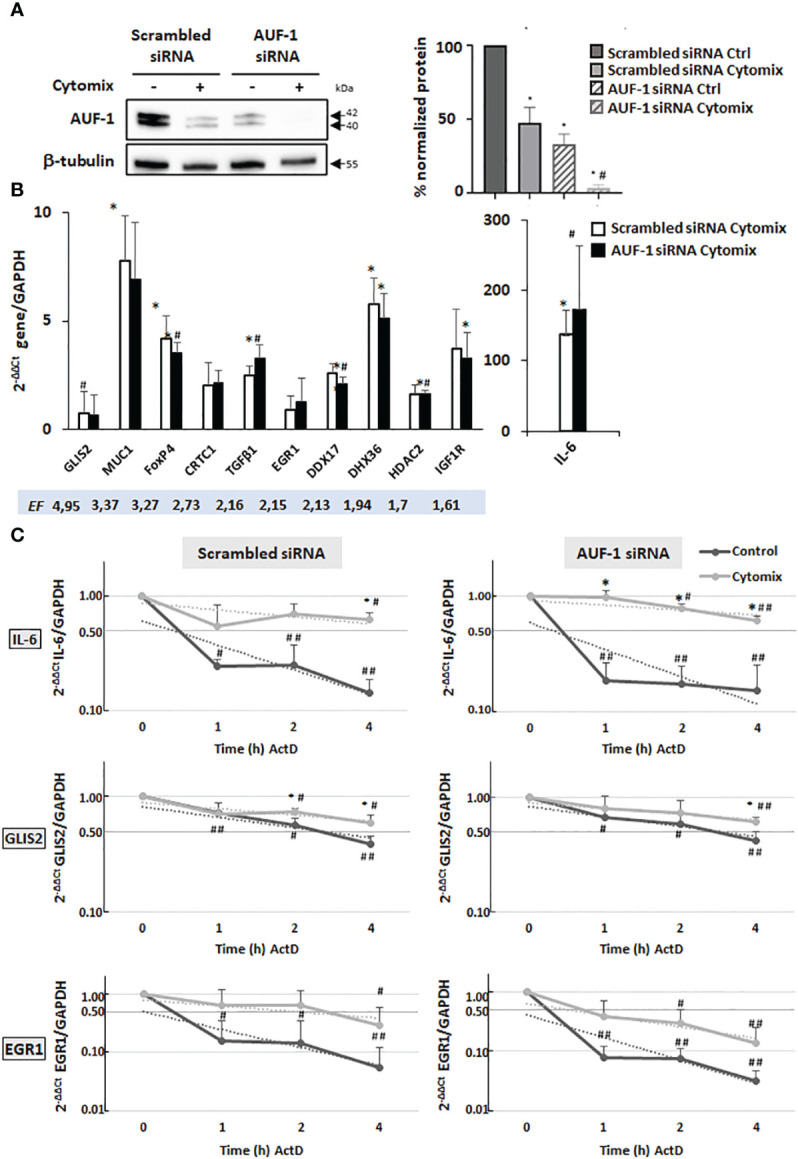
Analysis of AUF-1 target mRNA decay according to changes in AUF-1 intracellular levels. **(A)** Representative immunoblot (upper panel) and densitometric analysis (lower panel) of AUF-1 levels after transfection with scrambled siRNA or AUF-1 siRNA (48 h) and subsequent culture (48 h) with cytomix or medium control (mean ± SEM of n=3). β-tubulin was detected as the loading and normalization control. *p<0,05 compared to the scrambled-transfected medium control, #p<0,05 compared to AUF-1-siRNA (siAUF-1) control. **(B)** qRT-PCR analysis of steady-state mRNA expression of indicated AUF-1 targets, listed by decreasing EF, shown as housekeeping gene-normalized Ct value as fold change over unstimulated condition (2^-ΔΔCt^). *p<0,05 cytomix compared to the scrambled-transfected medium control, #p<0,05 compared to AUF-1-siRNA (siAUF-1) control. **(C)** qRT-PCR analysis (mean ± SEM of n=3) of mRNA decay rate of IL-6, a known AUF-1-regulated gene, and AUF-1 targets GLIS2 and EGR-1 upon treatment with actinomycin D (ActD) for indicated times after 48 h of cytomix stimulation (Time 0). Target mRNA expression levels were normalized to housekeeping mRNA (GAPDH) and expressed for each timepoint as fold change over time 0, as 2^-ΔΔCt^. *p<0,05 cytomix value *vs* corresponding unstimulated control value (CTRL in legend) at each datapoint; #p<0,05 and ##p<0.01 for ActD time points vs t=0 in each condition.

We previously documented that cytomix- and CSE-induced loss of AUF-1 in BEAS-2B cells occurred along with changes in expression levels of many established AUF-1-regulated cytokines and chemokines. This modulation was replicated with greater AUF-1 loss induced by siRNA (48). We hypothesized that loss of AUF-1 occurring upon cytomix stimulation might be reflected in changes in mRNA stability of its newly identified epithelial targets. We therefore evaluated steady-state levels and mRNA decay rates of selected targets displaying different EF from the RIP-Seq analysis (GLIS2, MUC1, FOXP4, CRTC1, TGFβ1, EGR1, DDX17, DHX36, HDAC2, IGF1R). Since diseased phenotypes were already observable in heterozygous *Auf1* ^-/-^ mice (39, 43, 44), these parameters were evaluated in conditions associated with three different levels of AUF-1 expression (48): in resting cells (basal AUF-1 levels), cytomix-stimulated (lower AUF-1 levels compared to basal) and cytomix-stimulated, AUF-1 siRNA-transfected cells (near-complete AUF-1 loss), which we show to be present in the experimental system (**Figure 3A**): in BEAS-2B cells transfected with scrambled siRNA, cytomix stimulation significantly decreased basal AUF-1 protein level (by 53.1%). Upon AUF-1 silencing, AUF-1 levels in resting and cytomix-treated cells were reduced by 67% and by 96.6%, respectively, compared to scrambled siRNA-transfected, unstimulated cells.

We first examined steady-state mRNA levels of the chosen AUF-1 targets. In unstimulated cells, silencing of AUF-1 did not change significantly their basal expression (**Figure S2**). In cells stimulated with cytomix (**Figure 3B**), near-complete loss of AUF-1 by siRNA-mediated silencing reproduced the changes in expression induced by cytomix in scrambled siRNA-transfected cells, where AUF-1 levels were lowered by this treatment, with no further enhancement.

Examining mRNA decay by ActD assay, in cells transfected with scrambled siRNA (**Figure 3C**, left panels) cytomix induced mRNA stabilization for GLIS2 and EGR1 along with IL-6; however, AUF-1 silencing changed cytomix-induced decay rates differently (**Figure 3C**, right panels).

As expected for IL-6, in scrambled siRNA-transfected cells (left panel) cytomix treatment increased the mRNA stability (55%, 70% and 63% of mRNA remaining *vs* time 0) compared to resting cells (24%, 25% and 14% vs time 0, half-life: > 4 h in cytomix-treated *vs* 0.4 h in resting cells). In AUF-1-silenced cells (right panel), IL-6 mRNA stabilization by cytomix was further accelerated, with 96% remaining mRNA at 1h *vs* 55% left at 1h *vs* time 0 in scrambled-transfected cells.

Also for GLIS2 mRNA, in scrambled siRNA-transfected cells (left panel) cytomix treatment increased mRNA stability over unstimulated cells, with 74% and 60% mRNA remaining at 2 h and 4 h, respectively *vs* 56% and 39% left in resting cells *vs* time 0 (half-life: > 4 h in cytomix-treated *vs* 3 h in resting cells, a basal decay rate slower than IL-6); in AUF-1-silenced cells (right panel) no further stabilization occurred.

EGR1 mRNA had a yet different pattern according to partial or total loss of AUF-1. In scrambled siRNA-transfected cells (left panel), similar to IL-6 mRNA, cytomix treatment triggered a marked mRNA stabilization (64%, 63% and 30% *vs* time 0) over the rapid mRNA decay rate in unstimulated cells (16%, 15% and 5% *vs* time 0, half-life: 2 h in cytomix-treated *vs* 0.3 h in resting cells). Surprisingly, in AUF-1 silenced cells (right panel) EGR1 mRNA decay rate became faster in all timepoints in both cytomix-treated cells (39%, 30% and 14% *vs* time 0) and in resting condition (8%, 8% and 3% *vs* time 0, half-life: 0.5 h in cytomix-treated *vs* < 0.5 h in resting cells) though differences with the corresponding values in scrambled-transfected cells was statistically non-significant due to data variability.

For the remaining validated transcripts (MUC-1, FOXP4, CRTC1, TGFβ1, DDX17, DHX36, HDAC2 and IGF1R), we detected a slow rate of mRNA decay in resting cells with half-lives > 4 h, with small measurable changes induced by cytomix regardless of AUF-1 levels (**Figure S3**).

### AUF-1 loss induced by cytomix is associated with cellular senescence features in BEAS-2B and HSAEC

**Figure 4:**
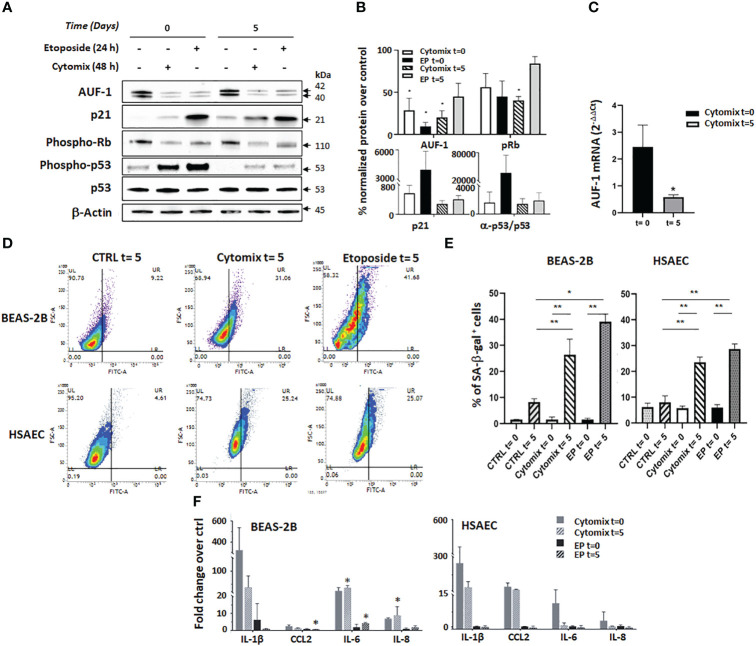
AUF-1 loss and cellular senescence in BEAS-2B and HSAEC upon cytomix stimulation. **(A, B)** Representative immunoblots **(A)** and densitometric analysis **(B)** of AUF-1, phospho-retinoblastoma (p-Rb) protein, p21, p53 and phospho-p53 after prolonged cell culture for senescence assays. BEAS-2B cell lysates were harvested after 48 h in resting or cytomix-stimulated conditions, or after 24 h etoposide stimulation (6 µM, as control inducer of senescence), as post-treatment time 0 and after additional 5 days of unstimulated cultures (as post-treatment time 5). β-actin was used as the loading control (mean ± SEM of n=3). *p<0,05 compared to corresponding unstimulated control. **(C)** qRT-PCR analysis of AUF-1 mRNA from BEAS-2B cells at time 0 and time 5 (mean ± SEM of n=3). mRNA levels were normalized to housekeeping mRNA levels (GAPDH) and expressed as fold change over unstimulated cells as 2^-ΔΔCt^. **(D, E)**. Representative contour plots of SA-β-gal activity **(D)** in BEAS-2B cells (upper panels) and HSAEC cells (lower panels). **(E)** Relative mean MFI upon cytomix stimulation for 48 h or etoposide for 24 h (mean ± SEM of n=3). After stimulation, the culture media was replaced with no further stimulation and cells were incubated (37°C, 5 days). **(F)** Detection of SASP –related cytokines in BEAS-2B (left panel, mean ± SEM of n=3) and HSAEC (right panel, mean ± SEM of n=2) supernatants upon indicated conditions. Cytokine levels are represented as fold change of mean fluorescence intensity over values in unstimulated (control) cell supernatants. *p<0,05, **p<0,01 *vs* corresponding control.

Findings in *Auf1^-/-^* KO mice indicate that its loss favours exaggerated cytokine responses and promotes cellular senescence through multiple mechanisms, providing a strong rationale for pathogenic relevance of the described decrease in AUF-1 bronchiolar expression in COPD (48). We therefore investigated whether epithelial cell senescence was present in conditions of AUF-1 loss, modelled by cytomix stimulation. We assessed this in BEAS-2B and also in HSAEC, in which we confirmed the decrease in AUF-1 protein by cytomix without concurrent changes in mRNA levels, and lack of changes in expression of other two relevant RBPs, TTP and HuR (**Figure S4**), as previously reported in BEAS-2B (48). Both cell types were stimulated with cytomix for 48 h or with low-dose etoposide (6 μM) as trigger control for senescence (67, 116) for 24 h (**Figure 4A, B**). At the end of the treatment period, set as time 0, culture media was replaced without adding any further stimulus and cells were incubated for 5 days, set as time 5.

Immunoblot analysis showed that cytomix induced a significant decrease of AUF-1 that persisted at time 5 (28.7% and 20.1% of corresponding controls at time 0 and 5, respectively). Importantly, levels of AUF-1 were significantly reduced also by etoposide treatment, more markedly at time 0 than at time 5 (9.6% and 45.0% of expression in controls, respectively). In the same experiments, markers of senescence displayed corresponding time-dependent changes: cytomix pre-stimulation significantly decreased levels of phospho-Retinoblastoma (56.1 and 40.4% of controls at time 0 and 5, respectively), it increased levels of the cyclin-dependent kinase (CDK) inhibitor p21 (563.4 and 280.1% of controls at time 0 and 5, respectively) and of phospho-p53/p53 ratio (1596,6% and 1407,7% of controls at time 0 and 5, respectively). A similar effect was induced by etoposide pre-treatment, which induced a decrease in phospho-Retinoblastoma levels (45.0% and 84.1% of controls at time 0 and 5, respectively), an increase in p21 levels (3998.4% and 404.4% of controls at time 0 and 5, respectively) and of phospho-p53/p53 ratio (37364,5 and 1887,9% of controls at time 0 and 5, respectively). Overall, cytomix effects were less marked, or comparable in amplitude, to those exerted by etoposide yet they were more persistent, being in all cases present at time 5, although experimental variability hampered in some case the finding’s statistical significance. Concurrently, real time-PCR showed significant decrease of mRNA levels of AUF-1 (42% inhibition over unstimulated control) after 5 days of cytomix stimulation, in contrast to unchanged mRNA levels determined after 48 h, at time 0 (**Figure 4C**).

In the same model, flow-cytometric senescence-associated β-galactosidase (SA-β-gal) assay detected a consistent and significant increase in β-gal activity upon cytomix treatment at time 5 in both cell types (up to 26.4% for BEAS-2B and 23.6% for HSAEC vs controls, p ≤ 0.05) which was in this case comparable in amplitude and duration to the effect seen at time 5 with etoposide treatment (39.1% for BEAS-2B and 28.7% for HSAEC vs controls, p ≤ 0.05) (**Figures 4D, E**).

Lastly, we evaluated in the culture supernatants the expression of SASP-related inflammatory mediators IL-1β, CCL2 (MCP-1), IL-6 and IL-8 (117) using LEGENDplex immunoassays (**Figure 4F**). In this setting, cytomix and etoposide displayed markedly different effects. In BEAS-2B cells (left panel), cytomix stimulation induced a robust and prolonged upregulation of IL-1β, IL-6 and IL-8 with little CCL2 modulation, in contrast with little or no cytokine increase by etoposide treatment. Differently from BEAS-2B, in HSAEC (right panel) cytomix induced a cytokine profile with smaller and more transient IL-6 and IL-8 upregulation, while IL-1β and CCL2 levels remained elevated at time 5. Despite increased β-gal activity (**Figure 4D, E**), in HSAEC etoposide did not elicit cytokine release.

These results suggest that cytomix pre-stimulated cells, while expressing low levels of AUF-1 also underwent cell cycle arrest and displayed features of senescent phenotype, likely with mechanisms only partially common to those occurring in etoposide-treated cells.

### Cytomix-induced enrichment of AUF-1 in extracellular vesicles

**Figure 5:**
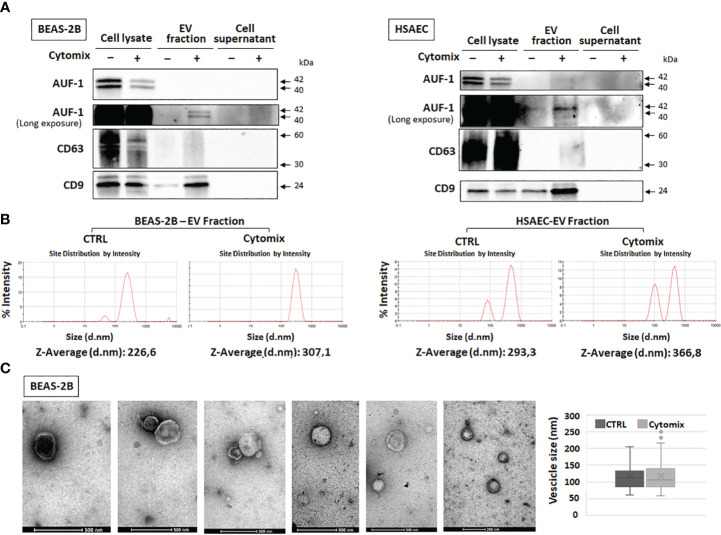
Detection of AUF-1 in extracellular vesicles (EVs) from cytomix-stimulated BEAS-2B and HSAEC. **(A)** Representative immunoblots (n=3) of AUF-1 in whole cell lysates, EV fractions and remaining supernatants (as EV isolation control) obtained by differential centrifugation of culture media of BEAS-2B (left panel) and primary HSAEC cells (right panel) in the indicated conditions, showing cytomix-induced changes in AUF-1 cellular and extracellular fractions. CD63 and CD9 were used as markers for EVs. **(B)** Dynamic light scattering (DLS) analysis showing the average size of EVs isolated from resting and cytomix-treated BEAS-2B (left panels) and HSAEC (right panels). **(C)** Representative transmission electron microscopy (TEM) images of EVs isolated from BEAS-2B cells; graph shows mean ± SD EVs size in experimental conditions. Scale bars are shown.

**Figure 6:**
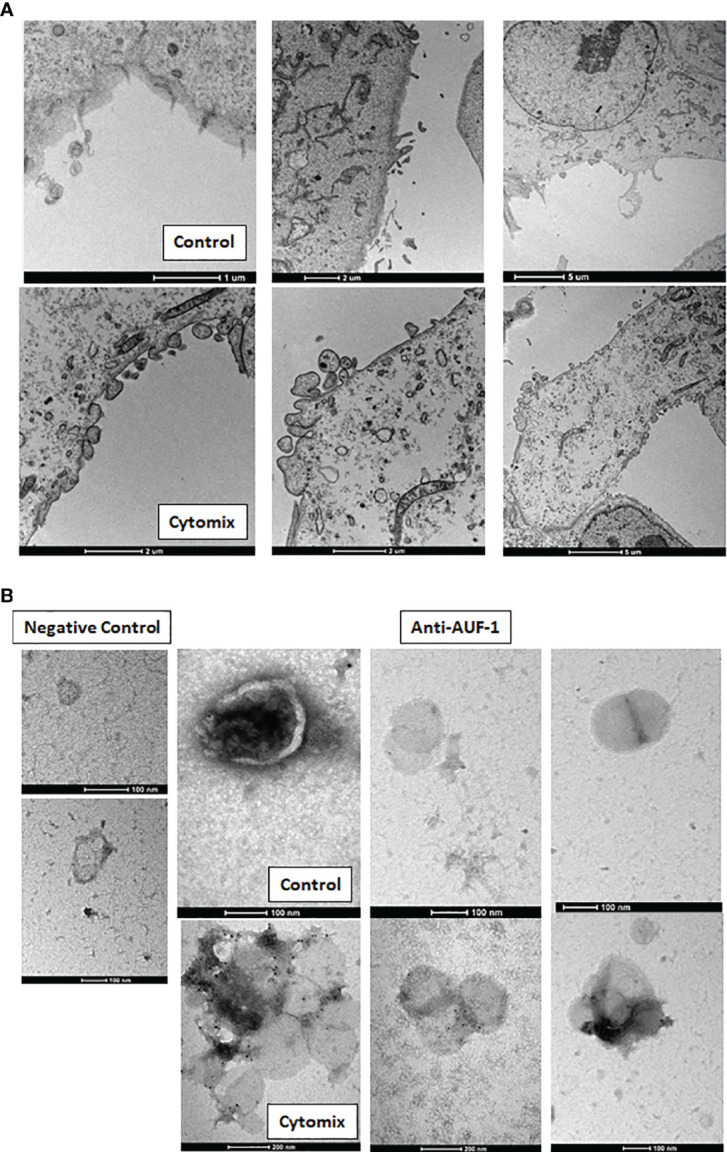
Cytomix-induced morphological changes in BEAS-2B cell monolayers and AUF-1 detection by immunogold labelling of BEAS-2B-derived EVs. **(A)** Representative TEM images of BEAS-2B cells monolayer in basal conditions (upper panels) and with cytomix stimulation (lower panels). Scale bars are shown. **(B)** Immunogold labelling for AUF-1in EVs derived from BEAS-2B cells in basal conditions (upper panels) and with cytomix stimulation (lower panels). Left panels show the negative control (isotype matched Ab) for immunogold staining. Scale bars are shown.

Given that AUF-1 mRNA expression was unchanged upon 48h cytomix stimulation in HSAEC (**Figure S4**) and in BEAS-2B (48) and decreasing significantly only at time 5 (**Figure 4C**), we initially evaluated whether cytomix decreased AUF-1 protein *via* proteasome-regulated degradation (118, 119). Cell preincubation with proteasome inhibitor MG 132 (10 μM) did not increase AUF-1 protein in resting and cytomix-treated BEAS-2B (**Figure S5**). In parallel, we assessed whether cytomix triggered an increase in EVs with relative transfer of AUF-1 in this compartment. Supernatants from resting and cytomix-treated BEAS-2B and HSAEC cells, both seeded at equal cell density/condition, were collected and EV isolated by differential centrifugation were characterized by immunoblot, DLS and TEM. Immunoblot analysis (**Figure 5A**) revealed that in parallel with the decrease in intracellular AUF-1 levels, in both cell types cytomix induced an increase in AUF-1 detection in the extracellular EVs fraction. Together with AUF-1, CD63 and CD9 were included as markers for EVs, and supernatants of EVs pellet were also loaded to confirm isolation of EVs. DLS analysis (**Figure 5B**) confirmed the release of particles with similar size range in BEAS-2B (average size of 226.6 nm and 307.1 nm) and in HSAEC (average size of 293.3 nm and 366.8 nm) in resting and cytomix-treated cells. Quantification of EVs by NTA analysis showed a concentration of 3.94e+10 ± 1.71e+09 (mean ± SEM) particles/ml and 8.88e+10 ± 2.34e+09 particles/ml released by resting and cytomix-stimulated cells, respectively; the mean ± SEM size in these conditions were 193.1 ± 1.5 nm and 209.7 ± 0.7 nm, respectively. Further analysis by TEM of EVs from BEAS-2B cells (**Figure 5C**) revealed EVs of spherical shape surrounded by a bilayer. Assessment of particle size by Image software by randomly selected vesicles (100 in each condition) indicated a mean average diameter of 116.73 nm in unstimulated and 117 nm in cytomix-stimulated samples. Differences in size measurement in TEM compared to DLS for EVs have been previously documented (120, 121). TEM analysis of the corresponding BEAS-2B cell monolayers (**Figure 6A**) showed an enrichment of membrane protrusions in cytomix-stimulated cells (lower panels) suggestive of budding vesicles. Immunogold labelling with anti-AUF-1 antibody (**Figure 6B**) revealed detectable staining in EVs, further supporting localization of AUF-1 in this extracellular compartment.

### Expression of AUF-1-associated transcripts in primary airway epithelial transcriptome and lung biopsy databases of COPD patients versus control subjects

**Figure 7:**
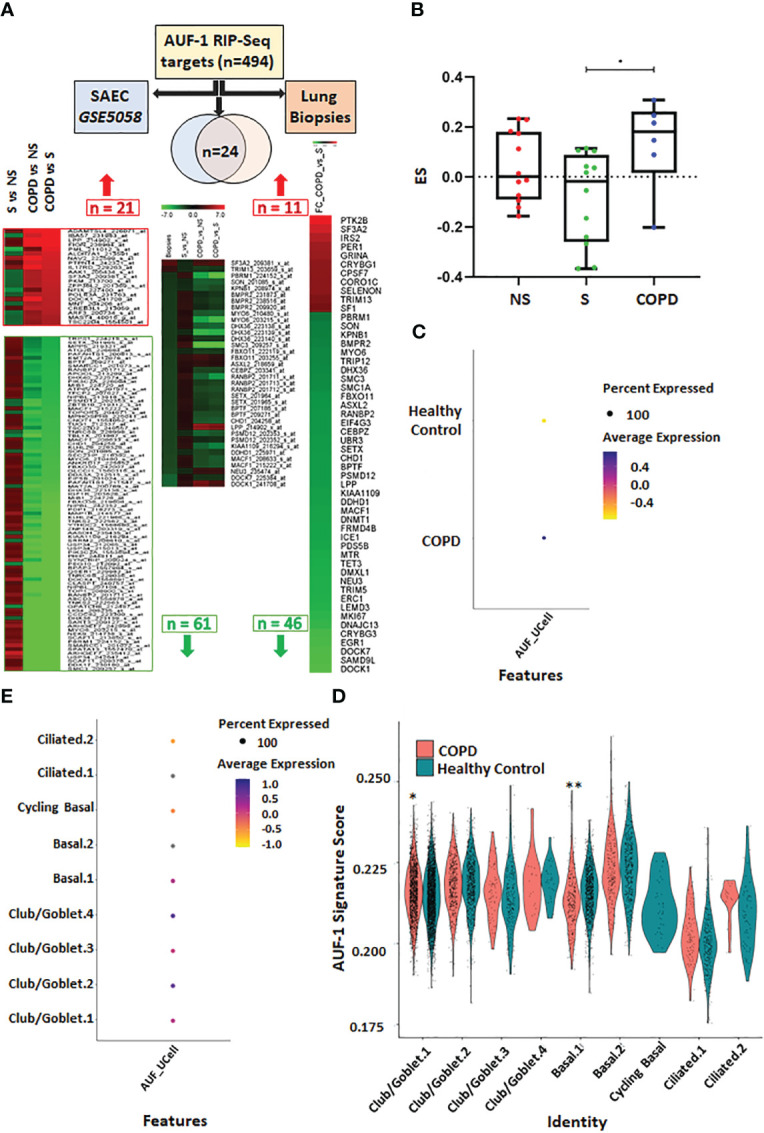
Analysis of AUF-1-bound transcript pool expression in transcriptomic databases of small airway/broncho-epithelium, lung biopsies and of single cell RNA-sequencing (scRNA-Seq) of COPD. **(A)** Analysis of AUF-1 target expression identified by RIP-Seq (n=494) in small airway epithelial cell (SAEC) gene array database GSE5058 (left-pointing arrow) (full list in **Table S5**) and in RNA sequencing database of lung biopsies of COPD vs smokers with NLF (right-pointing arrow) (full list in **Table S6**). *For GSE5058 data:* heatmap showing probes, indicated with Gene name and Probe ID, of 21 upregulated and 66 downregulated AUF-1 targets identified as DEG (FC ≥ |2.0|, FDR ≤ 0.5) in COPD *vs* smoker controls with differential expression in (left to right): smokers *vs* non-smoker controls; COPD *vs* non-smokers; COPD *vs* smoker controls. *For lung biopsies data:* heatmap showing FC of 57 AUF-1 targets, identified as DEG (log_2_FC ≥ |0.40|, FDR ≤ 0.5) in COPD vs smoker controls. *Central arrow down*: Venn diagram indicating overlap of n=24 AUF-1 targets shared by the two databases and heatmap showing relative expression. **(B)** Gene signatures identified by GSVA in GSE5058 dataset showing the enrichment score (ES) of the AUF-1 transcript targets (EF≥2 and FDR ≤ 0.05) in non-smokers (NS), smokers (S) and chronic obstructive pulmonary disease (COPD) patients. *p ≤ 0.05(Student’s t-test). **(C, D)** Signature plots of global **(C)** and cell-specific **(D)** average expression of AUF-1 signature, *p ≤ 0.005, ≤ 0.000005** (Student’s t-test). **(E)** Expression of AUF-1 signatures in individual broncho-epithelial cell types, all generated from scRNAseq datasets of primary broncho-epithelial cells from stable COPD patients and healthy controls differentiated at the air-liquid-interface (82).

Given decreased expression of AUF-1 in COPD epithelium (48), changes in the 494 RIP-Seq-derived AUF-1 target mRNAs levels were investigated in multiple transcriptomic studies of airway bronchiolar epithelium, to further study the potential impact of AUF-1 regulation in airway epithelial responses. A first analysis was conducted in a public microarray database from small airway epithelium obtained by bronchial brushings of stable COPD patients, smokers and non-smokers both with NLF (GEO ID: GSE5058) (79) (**Figure 7A**). Genes whose relative probes showed discordant FC values (up- and down-regulated) were not included in the total gene count. Out of the 494 genes, 150 (30%) were differentially expressed genes (DEG) in COPD patients *vs* smokers with NLF (FC ≥ |1.5|, FDR ≤ 0.05), with the large majority (102 of them, 66% of DEG, with FC ≤ -2) down-regulated, with changes of decreasing amplitude in non-smokers and healthy controls (**Figure 7A**, **Table S5**). The same search was implemented in a newly generated RNA sequencing database from whole lung biopsies of stable moderate-to severe COPD patients and age- and smoking history-matched smoker subjects with NLF (**Table S3**). Fifty-two (10%) of the AUF-1- bound target mRNA were expressed as DEG, and in this case as well the majority (41 of them, 79%) were significantly downregulated (log_2_FC ≤ -0.40) (**Figure 7A**, **Table S6**). Cross comparisons of the two AUF-1 target DEG lists identified 24 common transcripts, also in this case largely down-regulated, with only 4 genes (*LPP, SF3A2, NEU3, DOCK1*) upregulated. These 24 transcripts encode for a core of DNA/RNA binding proteins involved in regulation of transcription, DNA repair and genomic stability, telomere maintenance, RNA metabolism and translation, which are all within described AUF-1 regulatory functions; a part of them is involved in cell adhesion, cytoskeletal rearrangements for motility, migration, endocytosis, phagocytosis and nucleocytoplasmic shuttling, and protein ubiquitination (**Table 3**).

**Table 3:** AUF-1 RIP-Seq-derived gene targets expressed as DEGS in COPD subjects vs smoker controls in both GSE 5058 and in RNAseq lung biopsies datasets (see **Figure 8A**; full AUF-1 Rip Seq-derived gene target lists in **Tables S4, S5**).

Gene Acronym	Gene Full name	Functions of encoded protein	Refs
**BMPR2**	Bone morphogenetic protein receptor type 2	Gene mutations are the main genetic cause of pulmonary arterial hypertension. BMPR2 signaling is anti-inflammatory in vascular endothelium. Genetic factor involved in the development of COPD. Expression is decreased by exposure to cigarette smoke in lung tissue samples.	(122–126)
**BPTF**	Bromodomain PHD Finger Transcription Factor	Facilitates access to DNA during DNA-templated processes such as DNA replication, transcription, and repair.	(127, 128)
**CEBPZ**	CCAAT Enhancer Binding Protein Zeta	DNA-binding as transcriptional activator, regulates the heat-shock protein 70 (HSP70) promoter. RNA binding.	(129, 130)
**CHD1**	Chromodomain Helicase DNA Binding Protein 1	ATP-dependent helicase Involved in transcription-related chromatin-remodeling. Associated with histone deacetylase (HDAC) activity. Targeted disruption of the CHD1 gene in human cells leads to a defect in early double-strand break (DSB) repair *via* homologous recombination (HR). Modulates the efficiency of pre-mRNA splicing in part through physical bridging of spliceosomal components to H3K4me3.	(131–134)
**DDHD1**	DDHD Domain Containing 1	Phospholipase required for the organization of the endoplasmic reticulum exit sites (ERES), also known as transitional endoplasmic reticulum (tER).	(135)
**DHX36**	DEAH-Box Helicase 36	ATP-dependent DNA/RNA helicase that unwinds G-quadruplex (G4) structures. Plays a role in genomic integrity. Plays a role in transcriptional regulation. Plays a role in post-transcriptional regulation. Binds also to ARE sequences present in several mRNAs mediating exosome-mediated 3’-5’ mRNA degradation. DHX36 regulates transcription, genomic stability, telomere maintenance, translation and RNA metabolism.	(136–143)
**DOCK1**	Dedicator Of Cytokinesis 1	Involved in cytoskeletal rearrangements required for phagocytosis of apoptotic cells and cell motility.	(144)
**FBXO11**	F-Box Protein 11	Substrate recognition component of a SCF (SKP1-CUL1-F-box protein) E3 ubiquitin-protein ligase complex which mediates the ubiquitination and subsequent proteasomal degradation of target proteins.	(145)
**KIAA1109**	as BLTP1, Bridge-Like Lipid Transfer Protein Family Member 1	Plays a role in endosomal trafficking and endosome recycling. Involved in the actin cytoskeleton and cilia structural dynamics. Acts as regulator of phagocytosis.	(146, 147)
**KPNB1**	Karyopherin Subunit Beta 1	Functions in nuclear protein import. Gene Ontology (GO) annotations related to this gene include RNA binding and enzyme binding.	(148)
**LPP**	LIM Domain Containing Preferred Translocation Partner In Lipoma	Localizes to the cell periphery in focal adhesions and may be involved in cell-cell adhesion and cell motility. Shuttles through the nucleus and may function as a transcriptional co-activator.	(149)
**MACF1**	Microtubule Actin Crosslinking Factor 1	F-actin-binding protein which plays a role in cross-linking actin to other cytoskeletal proteins and binds to microtubules. Plays a key role in wound healing and epidermal cell migration (By similarity).	(150, 151)
**MYO6**	Myosin VI	A reverse-direction motor protein that moves towards the minus-end of actin filaments. Functions in multiple intracellular processes such as vesicular membrane trafficking and cell migration. Required for the structural integrity of the Golgi apparatus *via* the p53-dependent pro-survival pathway.	(152, 153)
**NEU3**	Neuraminidase 3	Exo-alpha-sialidase. Plays a role in the regulation of transmembrane signaling through the modulation of ganglioside content of the lipid bilayer and by direct interaction with signaling receptors, such as EGFR. Desialylates EGFR and activates downstream signaling in proliferating cells.	(154, 155)
**PBRM1**	Polybromo 1	Component of ATP-dependent chromatin remodeling complex involved in transcriptional regulation of select genes by alteration of DNA-nucleosome topology). Acts as a negative regulator of cell proliferation.	(156, 157)
**PSMD12**	Proteasome 26S Subunit, Non-ATPase 12	Component of the 26S proteasome, multiprotein complex involved in the ATP-dependent degradation of ubiquitinated proteins.	(158)
**RANBP2**	RAN Binding Protein 2	A component of the nuclear pore complex, plays a role in facilitation of protein import and export, sumoylation of protein cargoes, intracellular trafficking, and energy maintenance.	(159)
**SETX**	Senataxin	Probable RNA/DNA helicase involved in diverse aspects of RNA metabolism and genomic integrity. Involved in DNA double-strand breaks damage response generated by oxidative stress. In association with RRP45, targets the RNA exosome complex to sites of transcription-induced DNA damage. May be involved in telomeric stability through the regulation of telomere repeat-containing RNA (TERRA) transcription. Contributes to the mRNA splicing efficiency and splice site selection.	(160–163)
**SF3A2**	Splicing Factor 3a Subunit 2	Involved in pre-mRNA splicing as a component of pre-catalytic spliceosome ‘B’ complexes.	(164–166)
**SMC3**	Structural Maintenance Of Chromosomes 3	Central component of cohesin, a complex required for chromosome cohesion during the cell cycle. Cohesion is coupled to DNA replication and is involved in DNA repair.	(167, 168)
**SON**	SON DNA And RNA Binding Protein	DNA/RNA-binding protein that acts as mRNA splicing cofactor. Specifically promotes splicing of many cell-cycle and DNA-repair transcripts.	(169, 170)

We further probed the transcriptomic profiles of GSE5058 database by applying GSVA analysis (78) to calculate the enrichment score of those AUF-1-RNA targets characterized by an EF≥2 and FDR ≤ 0.05 (n=73). The global gene signature was significantly enriched in COPD patients compared to smokers with NLF (difference of ES (dES) = 0.20; *p-value*<0.05) (**Figure 7B**). Lastly, we examined the expression of AUF1-bound epithelial targets in scRNA-Seq datasets generated from primary airway epithelial cells from stable COPD patients and healthy control subjects differentiated at ALI (82). The dataset contains transcriptomic profiles identifying nine different cell clusters included in three main cell types: club/goblet, basal and ciliated cells identified according to specific gene expression markers. Overall, AUF-1 targets were significantly overexpressed in COPD epithelial cell transcriptome over that of healthy subjects (**Figure 7C**), but expression profiles vary among the cell type clusters, with higher enrichment in basal.1 cells and lower in club/goblet.1. from COPD donors compared to healthy controls. The AUF-1 expression signature was also not detected in cycling basal and lower, although not statistically different, in ciliated.1 and ciliated.2 cells from COPD donors compared to healthy controls (**Figure 7D**). Differential expression analysis between COPD and healthy subjects showed that AUF-1 targets are specifically divergent in club/goblet.4 subcluster, as well as in cycling basal cells and in ciliated.2 cells (**Figure 7E**). Interestingly, club/goblet.4 subcluster was characterized by high expression of interferon response genes (82).

**Figure 8:**
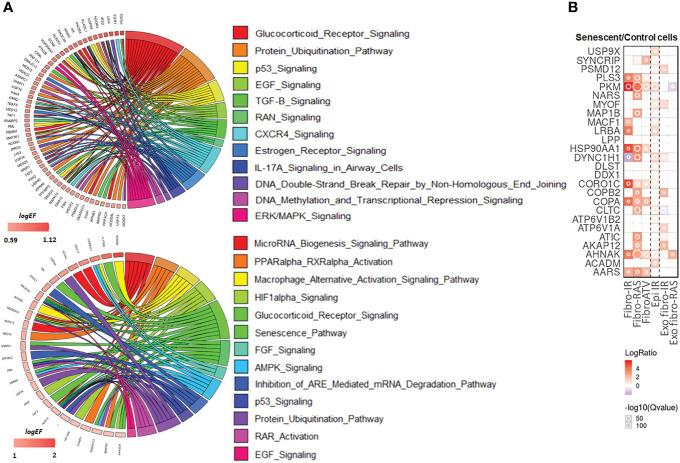
IPA analysis and representation in SASP proteome of AUF-1-bound transcript pool. **(A) Top**: Circos plot illustrating Gene log2EF values for canonical pathways identified for all RIP-Seq-derived AUF-1 targets (n=494) selected from the list of statistically over-represented (p-value ≤ 0.05) pathways (**Table 3**, **S3**), and (**bottom)** for those identified in the gene array database GSE5058 (n=150, **Table S4**). Coloured arks connect a gene to pathways. The thickness of the arks represents the number of differentially enriched genes belonging to that pathway. **(B)** Heatmap of Protein Secretomes-Ratio (Senescent/Control cells) of 26 AUF-1-associated transcripts, identified as soluble released protein or as exosomal cargo (Exo) in the SASP secretome database (www.saspatlas.com). * FDR-corrected q-value ≤ 0.05.

The list of 494 RIP-Seq-derived AUF-1 targets (**Table S4**) was subjected to Ingenuity Pathway Analysis (IPA) to map the main biological functions putatively affected by AUF-1. GO analysis demonstrated significant enrichment of epithelium-derived Canonical Pathways (CP) previously associated with COPD, or cigarette smoking and smoking-related lung disease (171, 172) such as intracellular and signalling molecules relevant in inflammation (SAPK, ERK/MAPK, CXCR4, EGF, TGFβ, FGF, GM-CSF, IL-9, IL17R, CCR3), cell cycle and DNA repair (p53, mitotic roles of polo-like kinase, DNA Double-Strand Break Repair by Non-Homologous End Joining), metabolism signalling (Aryl Hydrocarbon Receptor Signaling, PPARα/RXRα Activation, TR/RXR Activation) (**Figure 8A**, top circus plot; **Table 4** for selected pathways, full CP list in **Table S7**). Importantly, IPA performed over the 150 RIP-Seq-derived AUF-1 targets identified as DEG in COPD in the bronchiolar epithelium-derived transcriptomic GSE5058 database shared about a third of this profile but also acquired enrichment in important CP associated with cellular senescence and inflammation (senescence pathway, AMPK signaling, autophagy, macrophage alternative activation pathway) and with relevant and diverse posttranscriptional regulation (Inhibition of ARE-mediated mRNA degradation pathway, MicroRNA biogenesis signaling pathway, lnc HOTAIR regulatory pathway) (**Figure 8A**, bottom circus plot; **Table S8** for full CP list). Furthermore, AUF-1 targets were searched in the human SASP database of secreted proteins and exosomal cargo-associated SASP factors (www.SASPAtlas.com) originating from primary renal cortical epithelial cells and lung fibroblasts, in which senescence was induced by X ray-irradiation (IR) and inducible RAS overexpression (30). In this database, 26 AUF-1 targets were regulated in IR-induced senescent epithelial cells *vs* control, 10 of which (*ACADM, AHNAK, CLTC, DYNC1H1, LRB4, MACF1, MIOF, PKM, PSMD12, USP9X*) reached statistically significant increases (log_2_(SEN/CT) ≥ 0.58) (**Figure 8B**) and 7 (*AKAP12, ATP6V1A, CLTC, COPB2, DYNC1H1, MYOF, PSMD12*) were also found in exosome SASP of IR-induced senescent fibroblasts (log_2_(SEN/CT) ≥| 0.58|).

**Table 4:** Ingenuity Pathway Analysis (IPA)-derived selected canonical pathways of AUF-1 targets relevant to COPD.

**Ingenuity Canonical Pathways**	***p-*value**	**Ratio**	**Molecules**
**RANK Signaling in Osteoclasts**	8,31764E-05	9,62E-02	TRAF6,MAP3K9,MAPK14,PIK3C2A,PTK2B,MAP3K1,IRS2,PIK3R4,XIAP,PPP3CA
**Glucocorticoid Receptor Signaling**	0,000234	5,43E-02	PBRM1, PIK3C2A, SMAD3, MAP3K1, ARID2, PIK3R4, TRAF6, TAF1, AR, MAPK14, TGFB1, HSP90AA1, IRS2, NCOR1, NCOR2,SMARCC1, NRIP1, TAF2, PPP3CA
**PPARα/RXRα Activation**	0,000245	6,88E-02	CAND1,TRAF6,MAPK14,TGFB1,SMAD3,HSP90AA1,NCOA6,BMPR2,NR2C2,NCOR1,BCL3,NCOR2,MED12
**Protein Ubiquitination Pathway**	0,002344	5,17E-02	USP14,MED20,USP9X,BIRC6,DNAJC13,ANAPC1,XIAP,TRAF6,USP13,PSMD12,HSP90AA1,USP40,NEDD4L,USP34
**p53 Signaling**	0,003090	7,08E-02	MAPK14,PIK3C2A,PLAGL1,CCNK,IRS2,PIK3R4,PML,TP53BP2
**EGF Signaling**	0,003981	8,57E-02	MAPK14,PIK3C2A,ITPR2,MAP3K1,IRS2,PIK3R4
**TGF-β Signaling**	0,004677	7,29E-02	TRAF6,RUNX3,MAPK14,RNF111,TGFB1,SMAD3,BMPR2
**RAN Signaling**	0,005370	1,76E-01	KPNB1,RANBP2,KPNA6
**CXCR4 Signaling**	0,006456	5,46E-02	DOCK1,PXN,PAK4,PIK3C2A,ITPR2,GNA12,EGR1,PAK2,IRS2,PIK3R4
**Estrogen Receptor Signaling**	0,009772	5,84E-02	MED13,TAF1,MED20,NCOR1,NCOR2,NRIP1,MED12,TAF2
**Role of NFAT in Cardiac Hypertrophy**	0,011220	4,8E-02	MAPK14,HDAC2,PIK3C2A,CAMK1D,ITPR2,TGFB1,MAP3K1,IGF1R,IRS2,PIK3R4,PPP3CA
**SAPK/JNK Signaling**	0,012302	6,09E-02	MAP3K9,PIK3C2A,CRKL,GNA12,MAP3K1,IRS2,PIK3R4
**Role of Osteoblasts, Osteoclasts, Chondrocytes in Rheum Arthritis**	0,013803	4,66E-02	TRAF6,MAPK14,PIK3C2A,PTK2B,TGFB1,BMPR2,IRS2,TCF7L1,PIK3R4,XIAP,PPP3CA
**Osteoarthritis Pathway**	0,016982	4,72E-02	SIK3,FN1,GLIS2,GLI3,TGFB1,SMAD3,CTNNA1,BMPR2,RBPJ,TCF7L1
**TNFR1 Signaling**	0,022387	0,08	PAK4,PAK2,MAP3K1,XIAP
**B Cell Receptor Signaling**	0,027542	4,57E-02	MAP3K9,MAPK14,PIK3C2A,PTK2B,EGR1,MAP3K1,IRS2,PIK3R4,PPP3CA
**IL-17A Signaling in Airway Cells**	0,029512	6,25E-02	TRAF6,MAPK14,PIK3C2A,IRS2,PIK3R4
**CD40 Signaling**	0,030902	6,17E-02	TRAF6,MAPK14,PIK3C2A,IRS2,PIK3R4
**CCR3 Signaling in Eosinophils**	0,032359	0,05	PAK4,MAPK14,PIK3C2A,ITPR2,PAK2,IRS2,PIK3R4
**DNA Double-Strand Break Repair by Non-Homologous End Joining**	0,035481	1,43E-01	LIG4,LIG3
**DNA Methylation, Transcriptional Repression Signaling**	0,036307	8,82E-02	MECP2,HDAC2,DNMT1
**ERK/MAPK Signaling**	0,037153	4,33E-02	DOCK1,PXN,PAK4,PIK3C2A,PTK2B,CRKL,PAK2,IRS2
**Nitric Oxide Signaling in the Cardiovascular System**	0,038018	5,22E-02	PIK3C2A,ITPR2,HSP90AA1,IRS2,PIK3R4,PDE1C
**IL-23 Signaling Pathway**	0,040738	6,67E-02	RUNX1,PIK3C2A,IRS2,PIK3R4
**Choline Degradation I**	0,042657	0,5	ALDH7A1
**Cardiac Hypertrophy Signaling**	0,048977	3,94E-02	MAP3K9,MAPK14,PIK3C2A,TGFB1,GNA12,MAP3K1,IGF1R,IRS2,PIK3R4,PPP3CA

Full list in **Supplementary Table S4**.

### *In silico* validation of AUF-1 targeted transcripts

Full length p45^AUF-1^ protein sequence (containing all exons) was submitted to target search through CatRAPID algorithm, which estimates the binding propensity of protein-RNA pairs (62). This bioinformatic analysis listed 3,367 coding genes as putative AUF-1-binding targets. Of these, 123 genes were expressed in our Input dataset, with TPM cutoff ≥ 0.5 in a least one biological replicate and showed a DP≥0.75 computed by catRAPID tool. Of these 123 computationally derived AUF-1 targets, 70 (56.9%) were shared with the RIP-Seq experimental dataset (**Figure S6**, **Table S9**). Some transcripts of particular interest for COPD emerged, such as HDAC2, a deacetylase critically involved in epigenetic control of inflammatory responses whose expression and activity are repressed in COPD in several cell types (173, 174).

## Discussion

In COPD, the broncho-epithelium displays over-expressed inflammatory responses and features of accelerated aging (117, 175, 176). A putative role of AUF-1 in this disease setting was suggested by several phenotypic features of *Auf1* ^-/-^ mice, such as exaggerated LPS-induced cytokine responses, spontaneous accelerated cellular senescence, accelerated muscle wasting and altered B cell maturation (39, 43, 44). We then reported the selective loss of AUF-1 *ex-vivo* in the broncho-epithelium of patients with stable moderate COPD and *in vitro* in cytokine- and cigarette smoke-stimulated airway epithelial cells (48). Hence, we set up to investigate the role of AUF-1 in airway epithelial responses in COPD by identification of its targeted transcripts, evaluating the expression of this gene signature in diverse COPD epithelial transcriptomic studies and studying mechanisms and effects of its downregulation, triggered by inflammatory stimulation or gene silencing, in cultured human airway epithelial cells.

RIP-Seq analysis yielded 494 transcripts associated with AUF-1 in cytoplasmic lysates from resting BEAS-2B cells. Although a number of non-specific or indirect target associations needs to be expected when using RIP (177), we successfully validated all transcripts selected for biotin pulldown analysis. Furthermore, more than 50% of putative transcripts identified *in silico* as targetable by AUF-1 and expressed in the input sequencing were included in the RIP-Seq experimental epithelial dataset, increasing the confidence on the dataset as representative of AUF-1-targeted transcripts in unperturbed conditions.

Increased resolution of methods investigating RNP interfaces have expanded the knowledge on binding motifs by which RBPs coordinate multiple transcripts (177). AUF-1 has been primarily defined for its high-affinity binding to adenylate/uridylate-rich elements (ARE) (8, 113, 178, 179). Our RIP-Seq-derived AUF-1 target list were almost fully included among those identified by Yoon et al. (47) which described AUF-1 binding to GU-and U-rich regions located in the intronic regions and 3’UTR of targeted transcripts. The motif was identified in HEK293 cells through PAR-CLIP analysis, a high-resolution method for identification of RBP binding sequences, using AUF-1 isoform-specific overexpressing systems (47). However, our analysis has instead uncovered a predominantly GC-rich signature in the 3’UTR of targets associating to the endogenous cytoplasmic levels of AUF-1. We validated by biotin pulldown AUF-1 association with a synthetic GC-rich motif based on those generated by computational analysis of targets 3UTRs. This new finding needs to be further defined functionally by studies in isolated 3’UTR-reporter assays. In general, GC-rich elements are conserved in coding and non-coding regions of mammalian mRNAs, similar to AREs (180). They have been identified in transcripts associated with the RBPs nucleolin, PCBP1 [Poly(RC) Binding Protein 1] and UPF (181) and regulate target mRNA stability/decay and translational efficiency (182), being enriched in genes involved in metabolism and immune responses (183).

Our analysis of the effect of AUF-1 loss on the steady state and mRNA stability of selected validated targets was based on the hypothesis that the documented AUF1 loss – *ex vivo*, in COPD’s bronchiolar epithelium, or *in vitro* by cytomix – would be functionally reflected by the changes, brought by cell activation, in the AUF-1 targets identified by RIP-seq. When lowering AUF1 with siRNA, we would therefore expect to reproduce - or possibly enhance - the effects seen with cytomix stimulation; to this end, although correlative in nature, the results shown in **Figure 3B** do reproduce the cytomix effect on the newly identified AUF-1 mRNA targets, as it did on cytokines previously shown to be regulated by AUF-1 (48). The lack of changes in steady-state levels of the validated targets in unstimulated cells where AUF-1 was silenced may either indicate a marginal role of AUF-1 in this state or rather, that stimulus-induced posttranslational modifications are necessary for AUF-1 activation and specific functional outcomes (184). Moreover, the well-established mRNA decay-promoting function of AUF-1 is more consistently associated to ARE-mediated transcript binding (113), while a more heterogeneous and complex functional profile for AUF-1 has been described for the AUF-1 target pool enriched for the GU/U-rich elements (47). In our study, AUF-1 functional outcomes on mRNA target levels and decay rates were also heterogeneous and only partially dependent on the relative amounts of AUF-1 available: while as expected, the stability of the ARE-bearing IL-6 mRNA increased progressively along with decreased or near-complete loss of AUF-1 - brought by cytomix or siRNA, respectively - the newly identified AUF-1 targets displayed instead very different steady-state and decay patterns. Upon cytomix stimulation both GLIS2 and EGR1 (84, 85, 185, 186) showed little or no changes in mRNA steady state levels but increased their mRNA half-life; however, in cells with near-total loss of AUF-1 through silencing, the cytomix-induced stabilization of GLIS2 mRNA did not further increase (as it did for IL-6) while unexpectedly, EGR1 mRNA stabilization was even largely reversed. Furthermore, upon cytomix stimulation, some of the tested mRNA targets increased (FoxP4, TGFβ1, DDX17) or did not change (HDAC2) their steady state levels and showed highly stable mRNA at baseline. These decay rates were not modified by AUF-1 loss, suggesting a more downstream role in translational control or regulation by competing RBPs.

These results are likely being influenced by multiple factors that need to be further investigated: firstly, whether the enrichment of the GC-predominant motif in the AUF-1 targets’ 3’UTR conveys a specific function for this factor. Secondly, translation control by AUF-1 is complex: it may occur through competing binding of RBPs with translational repression functions, such as TIA and TIAR, associated with AUF-1 loss (187), or through direct AUF-1 binding to elF4G and poly(A) binding protein (43) or likely, also through association with other RBP or noncoding RNA upon specific activation signaling (179). To this end, changes in RBPs sharing binding affinity to AUF-1 motifs or engaging in protein-protein interaction with AUF-1, such as HuR (47) or TTP (188) may also occur with cytomix activation, as in we found no changes in their expression. These are likely key determinants that are still unprobed and need resolution with global proteomic approaches. Moreover, our expression and mRNA decay results are in line with the functional profile of AUF-1-associated transcripts identified by two studies, first by RIP (178) and subsequently by PAR-CLIP (47) which revealed a diversified functional repertoire, with subsets of targets canonically regulated through accelerated mRNA decay, other regulated instead through a positive effect on mRNA stability or translation and a significant group in which no changes was identified in depletion/overexpression experimental systems. To this end, it is important to underscore that AUF-1-mediated posttranscriptional regulation goes beyond regulation of mRNA stability and translation and entails also complex reciprocal regulation with miRNA synthesis and function and with long non-coding RNAs (179), and participation to early posttranscriptional events, as alternative splicing (189), potentially occurring in the context of inflammatory responses, yet still unprobed. Experimental approaches necessary to a mechanistic understanding of AUF-1 functions in lung disease models will need to consider this complexity.

Stress-induced epithelial cell senescence has been recently characterized in COPD, idiopathic pulmonary fibrosis (IPF) and lung cancer (117, 190). *In vitro* and *in vivo* models of CS challenge indicate that airway epithelial cells undergo accelerated senescence upon exposure (191) and we documented the loss of AUF-1 upon CS exposure in BEAS-2B cells (48). A relevant finding of this study supporting the role of AUF-1 in epithelial cell senescence is the persistent decrease of its protein levels, maintained 5 days after stimulation with cytomix in both BEAS-2B and HSAEC, associated with indices of increased lysosomal damage and cell cycle arrest, along with the expression of SASP-related cytokines. The novel finding of cytomix-triggered increase in β-galactosidase staining, of amplitude similar to the one induced by etoposide, suggests that the concurrent loss of AUF-1 is contributing to cytomix-induced cellular senescence, supporting the role of this RBP in inflammatory and accelerated senescence responses in COPD, as initially suggested by *Auf1* ^-/-^ mice phenotype and further supported by the presence of enhanced DNA damage in AUF-1 silenced cells (47). This evidence expands to AUF-1 targets in the SASP secretome: established targets and COPD determinants such as IL-1β (43), IL-6 (114) and, IL-8 (192) but also the newly identified AUF-1 targets that were found within the human SASP secretome atlas (30).

Taken together, these results reveal a putative role for epithelial AUF-1 expression in cell cycle-, DNA damage- and SASP-related responses. Loss of AUF-1 in chronic inflammatory contexts such as COPD may therefore critically contribute to complex ‘inflammaging’ responses. In support of such pathological role, loss of RBPs is a well described feature in cellular aging: the loss of the RBP HuR related to replicative senescence in cell lines and aged tissue has been long established, with multiple mechanisms involved (193–196). Recent studies are increasingly reporting the loss of several RBPs in *in vitro* and mouse models of cellular senescence, such as HuD (197), fragile X-related protein 1 (FXR1) (198), and cold shock domain containing E1 (CSDE1)/upstream of N-Ras (UNR) (199). The identification of a global RBP downregulation in two COPD bronchiolar epithelium transcriptomic databases (50) further connects these evidence to a role of AUF-1 in inflammaging through mechanisms yet to be uncovered, likely also related to its extracellular transfer in exosomes. Epithelial-derived exosomes and MV - which were detected, measured and then visualized by TEM in this study - contribute to lung inflammatory disease pathogenesis and the ensuing systemic component through multiple mechanisms (200–204). The novel observation of cytomix-induced detection of AUF-1 in EVs raises as well several hypotheses on its function. AUF-1 may be shuttled into EVs as a stimulus-specific process to preserve RNP-bound RNA targets and sustain their function, as shown for the RBPs Major Vault Protein (MVP) and others in several models (40, 41, 205, 206). Recent work by Xiao et al. showed that exosomes containing HuR derived from colon cancer cells were taken up by to BEAS-2B cells and triggered cell proliferation through inhibition of P21 expression, while cells receiving HuR-negative exosome exhibited growth arrest (206). Along the same lines, it can be envisioned that once EVs carrying AUF-1 and related targets are secreted in the airway fluid, they could contribute to spreading of inflammation and senescence to neighbouring epithelial cells and similarly affect function of macrophages or other cell types through delivery of AUF-1 bound transcripts and miRNAs (207–209).

Overall, the mechanisms underlying AUF-1 functions in regulating inflammatory and senescence-related transcripts do rely on features of target binding, but likely are critically determined by interactions with other RNA (miRNAs, lncRNA) and protein components of RNP complexes as well as post-translational RBP modifications (179), leaving much of mechanistic understanding yet to additional studies. Identifying AUF-1 function and partners in EVs, both as targeted mRNAs and associated RBPs, could shed more light on how AUF-1 may affect critical EV-related lung disease mechanisms.

Initial findings of AUF-1 loss of expression in stable COPD *vs* controls, along with *in vitro* evidence of its down-regulation by cytokines and CS, led us to consider that decreased AUF-1 levels co-existed with an epithelial gene expression profile strongly driven by inflammation, oxidative stress responses and accelerated aging. To this end we previously documented, along with the loss of AUF-1 expression, a significant representation of a manually curated list of AUF-1-regulated transcripts as DEGs in a transcriptomic database of broncho-epithelial cells from COPD patients *vs* control smokers (GEO ID: GSE5058) (48). In the current study, we searched for identification of the 494 RIP-Seq derived AUF-1-associated transcripts in human COPD, to recognize at translational level the impact of its regulation and support additional in-depth mechanistic studies. We provide multiple evidence of their significant representation in COPD-derived broncho-epithelium: first, we identified in the GSE5058 database 150 (~30%) of these genes as DEGs in COPD patients *vs* S and NS subjects; moreover, in this database GSVA analysis indicated the entire 494 AUF-1-associated transcript signature as significantly enriched in COPD patients compared to S subjects. Although this database was generated using the HG-U133 Plus 2.0 GeneChips covering a limited gene set (n=6500), it is highly valuable for the analysis of the more distal bronchiolar cells – in which AUF1 loss was specifically identified - in stable COPD patients, smoker and non-smoker controls. Secondly, 10% of the 494 RIP-Seq derived AUF-1-associated transcripts were also identified as DEG in RNA sequencing analysis of lung parenchyma from a cohort of stable COPD *vs* matched S subjects. The 24 RIP-Seq-derived AUF-1 targets shared by the two COPD datasets even more clearly are representative, for the first time in a human lung disease, of the complex gene regulatory function AUF-1 exerts, previously characterized by the preclinical models (43–45, 47): in fact a large part of these genes (*BPTF, CEBP2, CHD1, DHX36, PBRM1, SETX, SF3A2, SMC3, SON*) regulate transcription, genomic stability, telomere maintenance, translation and RNA metabolism with likely impact on cell cycle progression, apoptosis, DNA damage repair. Interestingly, a number of genes (*DDHD1, DOCK1, KIAA1109, KPNB1, LPP, MACF1, MYO6, RANBP*) are involved in intracellular trafficking, cell adhesion, cell motility, cytoskeletal rearrangement required for phagocytosis and with proteasomal degradation (*FBX011, PSMD12*). The predominant downregulation found for the RIP-Seq derived AUF-1-associated transcripts in both transcriptomic datasets (in particular for the 24 genes in common, only *SF3A2, DOCK1, LPP, NEU3* are upregulated) supports the hypothesis that in COPD disease, AUF1 loss may determine in airway epithelium a loss of protection of genomic integrity and altered gene regulation, concurring to accelerated cell senescence and inflammation. Finally, AUF-1 target analysis in scRNA-Seq datasets from COPD donors compared to healthy controls (82) brings additional complexity to its broncho-epithelial expression. Reduced expression in subsets of club.goblet and ciliated cells aligns with the finding of the majority of DEG/AUF-1 targets resulting downregulated in the other datasets analysed, while its enrichment in less mature basal.1 cells points to the potential relevance in repair responses. Further analysis of the detailed distribution of expression of the AUF-1 targets will be necessary to increase understanding of AUF1 regulation in this context.

Our current study and previous investigations (48) are the first providing evidence that the striking similarities between the accelerated senescence phenotype of AUF1^-/-^ mouse and cellular models and key features of COPD pathogenesis – premature aging, tissue senescence, inflammation, increased cancer risk – indicate that AUF-1 plays a role in this human lung disease. Our experimental design was shaped upon the initial finding of diminished expression of AUF-1 in broncho-epithelium both *ex vivo* and *in vitro* (48, 50). This is a challenging experimental system to interrogate *in vitro*, with several drawbacks for results’ interpretation. The AUF-1 targets were identified in unstimulated conditions with basal AUF-1 levels and may not represent fully those affected by AUF-1 regulation upon exposure to chronic pathogenic conditions. Moreover, the cytomix-induced migration of AUF-1 in EVs has restricted the use of over-expression models as a phenotype rescue strategy, extending the need for analysis of AUF-1 function in COPD to its extracellular component. To this end, stable AUF-1^-/-^ epithelial cell lines are an important tool currently in development to enable the improved understanding of the role of AUF-1 in intra- and extra-cellular compartments and implement a full phenotype rescue approach. More in-depth analysis of AUF-1 isoform-specific targets may also reveal further levels of regulatory complexity and specific functions.

Recent advances on the molecular underpinnings of protein–RNA interactions in disease pathogenesis and in nanotechnology are driving the development of small molecules that target the interplay between RBPs and RNA, which has been for long considered poorly druggable, driven by data on RBP pathogenic relevance in cancer (17, 210–215). So far, the understanding of the pathogenic role and potential for targeting RBPs in COPD is lacking, despite this condition being a major risk factor for the development of lung cancer. Identification of the global down-regulation of RBPs in epithelial COPD transcriptomic databases (50) and of TTP protein as a relevant player in a mouse CS-induced model of COPD (188) warrant further studies in this field. Discovery of disease-associated RBP profiles and their regulatory influence may identify novel molecules and mechanisms that may be useful as biomarkers for phenotypic traits and for other smoking-related diseases with increased lung cancer risk, as idiopathic lung fibrosis (117). Furthermore, specific RBP signatures may coordinately control pathogenic pathways or altered responses to treatment related to SASP, and therefore may be extended to COPD co-morbidities and other chronic diseases characterized by inflammaging such as heart disease, diabetes, and obesity. In these instances, the molecular resolution of the RBP-transcript interface could reveal elements targetable therapeutically.

## Data availability statement

The datasets presented in this study can be found in online repositories. The names of the repository/repositories and accession number(s) can be found at E-MTAB-12583 (Array Express).

## Ethics statement

The studies involving human participants were reviewed and approved by University Hospital of Messina Ethics Committee. The patients/participants provided their written informed consent to participate in this study.

## Author contributions

IS, LR, JD: RIP-Seq, Transcriptomic and proteomic data generation, data analysis, manuscript preparation. Participation to research design AN, MC, MV: Transcriptomic and proteomic data generation GG, DM, AS: acquisition AUF-1 RIP-Seq data and GO analysis EL: exosomal analysis PB, FC: TEM study design, data acquisition and interpretation PD, TS, LC: RNA-Seq data acquisition and analysis for COPD lung biopsy study SI, MJ, PW, AF, PH: in silico analysis of RIP-Seq-derived AUF-1 target dataset on scRNA-Seq data IA: in silico analysis of RIP-Seq-derived AUF-1 target dataset in COPD databases FN, GC: recruitment COPD study subjects, participation to study design. CS: Study design and coordination, manuscript preparation. All authors contributed to the article and approved the submitted version.

## Glossary

**Table:**

Actinomycin D	ActD
Adenylate/Uridylate-Rich Elements	ARE
Air-Liquid Interface	ALI
Antibody	Ab
AU-rich-element factor-1	AUF-1
Bovine Serum Albumin	BSA
Canonical Pathway	CP
Chemokine (C-C Motif) Ligand	CCL
Chemokine (C-X-C Motif) Ligand	CXCL
Chronic Obstructive Pulmonary Disease	COPD
Cigarette Smoke	CS
Cigarette Smoke Extract	CSE
Cold Shock Domain Containing E1	CSDE1
Cyclin-Dependent Kinase	CDK
DEAD-Box helicase 17	DDX17
difference of ES	dES
Differentially Expressed Genes	DEG
Dynamic Light Scattering	DLS
Early growth response 1	EGR1
Enrichment Factor	EF
Enrichment Score	ES
Extracellular Vesicles	EVs
False Discovery Rate	FDR
Forced Expiratory Volume in 1 s	FEV1
Forced Vital Capacity	FVC
Forkhead Box P4	FoxP4
Fragile X-Related Protein 1	FXR1
Gene Set Variation Analysis	GSVA
Genome Ontology	GO
GLIS Family Zinc Finger 2	GLIS2
Guanine-Cytosine	GC
Heteronuclear Ribonucleoprotein D	HNRNPD
Histone deacetylase 2	HDAC2
Human antigen R	HuR
Human Small-Airways Epithelium	HSAEC
Idiopathic Pulmonary Fibrosis	IPF
Immunoprecipitation	IP
Ingenuity Pathway Analysis	IPA
Interleukin	IL
Mucin 1	Cell Surface Associated, MUC1
Nanoparticle Tracking Analysis	NTA
Normal Lung Function	NLF
Posttranscriptional Gene Regulation	PTGR
Principal Component Analysis	PCA
Quantitative Real-Time PCR	qRT-PCR
Ribonucleoprotein complex	mRNPs
RNA Immunoprecipitation-Sequencing	RIP-Seq
RNA-Binding Proteins	RBPs
Senescence-Associated Secretory Phenotype	SASP
Senescence-Associated B-Galactosidase	SA-β-gal
Single cell RNA-Sequencing	scRNA-Seq
Standard Deviation	SD
Transcript Per Million	TPM
Transforming Growth Factor	TGF
Transmission Electron Microscopy	TEM
Tristetraprolin	TTP
Tumor Necrosis Factor	TNF
Untranslated Region	UTR
X ray-irradiation	IR

## References

[1] Iadevaia V Gerber AP. Combinatorial control of mRNA fates by RNA-binding proteins and non-coding RNAs. Biomolecules (2015) 5:2207–22. doi: 10.3390/biom5042207 PMC4693235 26404389

[2] Gehring NH Wahle E Fischer U. Deciphering the mRNP code: RNA-bound determinants of post-transcriptional gene regulation. Trends Biochem Sci (2017) 42:369–82. doi: 10.1016/j.tibs.2017.02.004 28268044

[3] Barreau C Paillard L Osborne HB. AU-rich elements and associated factors: are there unifying principles? Nucleic Acids Res (2005) 33:7138–50. doi: 10.1093/nar/gki1012 PMC1325018 16391004

[4] Glisovic T Bachorik JL Yong J Dreyfuss G. RNA-Binding proteins and post-transcriptional gene regulation. FEBS Lett (2008) 582:1977–86. doi: 10.1016/j.febslet.2008.03.004 PMC2858862 18342629

[5] Ray D Kazan H Cook KB Weirauch MT Najafabadi HS Li X. Hughes TR. A compendium RNA-binding motifs decoding Gene regulation Nat (2013) 499:172–7. doi: 10.1038/nature12311 PMC3929597 23846655

[6] Coppin L Leclerc J Vincent A Porchet N Pigny P. Messenger RNA life-cycle in cancer cells: emerging role of conventional and non-conventional RNA-binding proteins? Int J Mol Sci (2018) 19:650. doi: 10.3390/ijms19030650 29495341 PMC5877511

[7] Corley M Burns MC Yeo GW. How RNA-binding proteins interact with RNA: molecules and mechanisms. Mol Cell (2020) 78:9–29. doi: 10.1016/j.molcel.2020.03.011 32243832 PMC7202378

[8] White EJ Brewer G Wilson GM. Post-transcriptional control of gene expression by AUF1: mechanisms, physiological targets, and regulation. Biochim Biophys Acta (2013) 1829:680–8. doi: 10.1016/j.bbagrm.2012.12.002 PMC3664190 23246978

[9] Luo NA Qu YQ Yang GD Wang T Li RL Jia LT. Post-transcriptional up-regulation of PDGF-c by HuR in advanced and stressed breast cancer. Int J Mol Sci (2014) 15:20306–20. doi: 10.3390/ijms151120306 PMC4264168 25383675

[10] Wang H Ding N Guo J Xia J Ruan Y. Dysregulation of TTP and HuR plays an important role in cancers. Tumour Biol (2016) 37:14451–61. doi: 10.1007/s13277-016-5397-z 27644249

[11] Barton M Meyer MR. HuR-ry up: how hydrogen sulfide protects against atherosclerosis. Circulation (2019) 139:115–8. doi: 10.1161/circulationaha.118.036854 30592653

[12] Anderson P. Post-transcriptional control of cytokine production. Nat Immunol (2008) 9:353–9. doi: 10.1038/ni1584 18349815

[13] Anderson P. Post-transcriptional regulons coordinate the initiation and resolution of inflammation. Nat Rev Immunol (2010) 10:24–35. doi: 10.1038/nri2685 20029446

[14] Newman R McHugh J Turner M. RNA Binding proteins as regulators of immune cell biology. Clin Exp Immunol (2016) 183:37–49. doi: 10.1111/cei.12684 26201441 PMC4687516

[15] Stumpo DJ Lai WS Blackshear PJ. Inflammation: cytokines and RNA-based regulation. Wiley Interdiscip Rev RNA (2010) 1:60–80. doi: 10.1002/wrna.1 21956907 PMC3915420

[16] Turner M Díaz-Muñoz MD. RNA-Binding proteins control gene expression and cell fate in the immune system. Nat Immunol (2018) 19:120–9. doi: 10.1038/s41590-017-0028-4 29348497

[17] Muralidharan R Panneerselvam J Chen A Zhao YD Munshi A Ramesh R. HuR-targeted nanotherapy in combination with AMD3100 suppresses CXCR4 expression, cell growth, migration and invasion in lung cancer. Cancer Gene Ther (2015) 22:581–90. doi: 10.1038/cgt.2015.55 PMC4679684 26494555

[18] Hitti E Bakheet T Al-Souhibani N Moghrabi W Al-Yahya S Al-Ghamdi M. Systematic analysis of AU-rich element expression in cancer reveals common functional clusters regulated by key RNA-binding proteins. Cancer Res (2016) 76:4068–80. doi: 10.1158/0008-5472.can-15-3110 27197193

[19] Patial S Blackshear PJ. Tristetraprolin as a therapeutic target in inflammatory disease. Trends Pharmacol Sci (2016) 37:811–21. doi: 10.1016/j.tips.2016.07.002 PMC5030171 27503556

[20] Hong S. RNA Binding protein as an emerging therapeutic target for cancer prevention and treatment. J Cancer Prev (2017) 22:203–10. doi: 10.15430/jcp.2017.22.4.203 PMC5751837 29302577

[21] Ross EA Naylor AJ O’Neil JD Crowley T Ridley ML Crowe J. Treatment of inflammatory arthritis *via* targeting of tristetraprolin, a master regulator of pro-inflammatory gene expression. Ann Rheum Dis (2017) 76:612–9. doi: 10.1136/annrheumdis-2016-209424 PMC5446007 27597652

[22] Mohibi S Chen X Zhang J. Cancer the’RBP’eutics-RNA-binding proteins as therapeutic targets for cancer. Pharmacol Ther (2019) 203:107390. doi: 10.1016/j.pharmthera.2019.07.001 31302171 PMC6848768

[23] Soriano JB Kendrick PJ Paulson KR Gupta V Abrams EM Adedoyin RA. Prevalence and attributable health burden of chronic respiratory diseases, 1990-2017: a systematic analysis for the global burden of disease study 2017. Lancet Respir Med (2020) 8:585–96. doi: 10.1016/s2213-2600(20)30105-3 PMC7284317 32526187

[24] Global Initiative for Chronic Obstructive Lung Disease (GOLD). Global strategy for the diagnosis, management, and prevention of chronic obstructive pulmonary disease. GOLD (2021). www.goldcopd.org.

[25] Jones B Donovan C Liu G Gomez HM Chimankar V Harrison CL. Animal models of COPD: what do they tell us? Respirology (2017) 22:21–32. doi: 10.1111/resp.12908 27731525

[26] Dua K Malyla V Singhvi G Wadhwa R Krishna RV Shukla SD. Increasing complexity and interactions of oxidative stress in chronic respiratory diseases: an emerging need for novel drug delivery systems. Chem Biol Interact (2019) 299:168–78. doi: 10.1016/j.cbi.2018.12.009 30553721

[27] Kumar M Seeger W Voswinckel R. Senescence-associated secretory phenotype and its possible role in chronic obstructive pulmonary disease. Am J Respir Cell Mol Biol (2014) 51:323–33. doi: 10.1165/rcmb.2013-0382PS 25171460

[28] Kadota T Fujita Y Yoshioka Y Araya J Kuwano K Ochiya T. Emerging role of extracellular vesicles as a senescence-associated secretory phenotype: insights into the pathophysiology of lung diseases. Mol Aspects Med (2018) 60:92–103. doi: 10.1016/j.mam.2017.11.005 29146100

[29] Manandhar B Paudel KR Panth N Hansbro P Oliver BG Dua K. Applications of extracellular vesicles as a drug-delivery system for chronic respiratory diseases. Nanomed (Lond) (2022) 17:817–20. doi: 10.2217/nnm-2021-0384 35019729

[30] Basisty N Kale A Jeon OH Kuehnemann C Payne T Rao C. A proteomic atlas of senescence-associated secretomes for aging biomarker development. PloS Biol (2020) 18:e3000599. doi: 10.1371/journal.pbio.3000599 31945054 PMC6964821

[31] Abdelmohsen K Kuwano Y Kim HH Gorospe M. Posttranscriptional gene regulation by RNA-binding proteins during oxidative stress: implications for cellular senescence. Biol Chem (2008) 389:243–55. doi: 10.1515/bc.2008.022 PMC8481862 18177264

[32] Wang W. Regulatory RNA-binding proteins in senescence. Ageing Res Rev (2012) 11:485–90. doi: 10.1016/j.arr.2012.02.006 22414963

[33] Kim C Kang D Lee EK Lee JS. Long noncoding RNAs and RNA-binding proteins in oxidative stress, cellular senescence, and age-related diseases. Oxid Med Cell Longev (2017) 2017:2062384. doi: 10.1155/2017/2062384 28811863 PMC5547732

[34] Harley J Clarke BE Patani R. The interplay of RNA binding proteins, oxidative stress and mitochondrial dysfunction in ALS. Antioxid (Basel) (2021) 10. doi: 10.3390/antiox10040552 PMC8066094 33918215

[35] Stoecklin G Tenenbaum SA Mayo T Chittur SV George AD Baroni TE. Genome-wide analysis identifies interleukin-10 mRNA as target of tristetraprolin. J Biol Chem (2008) 283:11689–99. doi: 10.1074/jbc.M709657200 PMC2431067 18256032

[36] Hamilton T Novotny M Pavicic PJ Jr. Herjan T Hartupee J Sun D. Diversity in post-transcriptional control of neutrophil chemoattractant cytokine gene expression. Cytokine (2010) 52:116–22. doi: 10.1016/j.cyto.2010.04.003 PMC2919655 20430641

[37] Fan J Ishmael FT Fang X Myers A Cheadle C Huang SK. Chemokine transcripts as targets of the RNA-binding protein HuR in human airway epithelium. J Immunol (2011) 186:2482–94. doi: 10.4049/jimmunol.0903634 PMC3872785 21220697

[38] Ishmael FT Fang X Houser KR Pearce K Abdelmohsen K Zhan M. The human glucocorticoid receptor as an RNA-binding protein: global analysis of glucocorticoid receptor-associated transcripts and identification of a target RNA motif. J Immunol (2011) 186:1189–98. doi: 10.4049/jimmunol.1001794 PMC3017228 21148795

[39] Moore AE Chenette DM Larkin LC Schneider RJ. Physiological networks and disease functions of RNA-binding protein AUF1. Wiley Interdiscip Rev RNA (2014) 5:549–64. doi: 10.1002/wrna.1230 24687816

[40] Statello L Maugeri M Garre E Nawaz M Wahlgren J Papadimitriou A. Identification of RNA-binding proteins in exosomes capable of interacting with different types of RNA: RBP-facilitated transport of RNAs into exosomes. PloS One (2018) 13:e0195969. doi: 10.1371/journal.pone.0195969 29689087 PMC5918169

[41] O’Brien K Breyne K Ughetto S Laurent LC Breakefield XO. RNA Delivery by extracellular vesicles in mammalian cells and its applications. Nat Rev Mol Cell Biol (2020) 21:585–606. doi: 10.1038/s41580-020-0251-y 32457507 PMC7249041

[42] Sarkar B Xi Q He C Schneider RJ. Selective degradation of AU-rich mRNAs promoted by the p37 AUF1 protein isoform. Mol Cell Biol (2003) 23:6685–93. doi: 10.1128/mcb.23.18.6685-6693.2003 PMC193711 12944492

[43] Lu JY Sadri N Schneider RJ. Endotoxic shock in AUF1 knockout mice mediated by failure to degrade proinflammatory cytokine mRNAs. Genes Dev (2006) 20:3174–84. doi: 10.1101/gad.1467606 PMC1635151 17085481

[44] Sadri N Schneider RJ. Auf1/Hnrnpd-deficient mice develop pruritic inflammatory skin disease. J Invest Dermatol (2009) 129:657–70. doi: 10.1038/jid.2008.298 PMC4074411 18830269

[45] Pont AR Sadri N Hsiao SJ Smith S Schneider RJ. mRNA decay factor AUF1 maintains normal aging, telomere maintenance, and suppression of senescence by activation of telomerase transcription. Mol Cell (2012) 47:5–15. doi: 10.1016/j.molcel.2012.04.019 22633954 PMC3966316

[46] Wang W Martindale JL Yang X Chrest FJ Gorospe M. Increased stability of the p16 mRNA with replicative senescence. EMBO Rep (2005) 6:158–64. doi: 10.1038/sj.embor.7400346 PMC1299256 15678155

[47] Yoon JH De S Srikantan S Abdelmohsen K Grammatikakis I Kim J. PAR-CLIP analysis uncovers AUF1 impact on target RNA fate and genome integrity. Nat Commun (2014) 5:5248. doi: 10.1038/ncomms6248 25366541 PMC4291169

[48] Ricciardi L Col JD Casolari P Memoli D Conti V Vatrella A. Differential expression of RNA-binding proteins in bronchial epithelium of stable COPD patients. Int J Chron Obstruct Pulmon Dis (2018) 13:3173–90. doi: 10.2147/copd.s166284 PMC6190813 30349226

[49] Gerstberger S Hafner M Tuschl T. A census of human RNA-binding proteins. Nat Rev Genet (2014) 15:829–45. doi: 10.1038/nrg3813 PMC11148870 25365966

[50] Ricciardi L Giurato G Memoli D Pietrafesa M Dal Col J Salvato I. Posttranscriptional gene regulatory networks in chronic airway inflammatory diseases: in silico mapping of RNA-binding protein expression in airway epithelium. Front Immunol (2020) 11:579889. doi: 10.3389/fimmu.2020.579889 33178205 PMC7596416

[51] Casolaro V Fang X Tancowny B Fan J Wu F Srikantan S. Posttranscriptional regulation of IL-13 in T cells: role of the RNA-binding protein HuR. J Allergy Clin Immunol (2008) 121:853–9.e4. doi: 10.1016/j.jaci.2007.12.1166 18279945 PMC2666917

[52] Keene JD Komisarow JM Friedersdorf MB. RIP-chip: the isolation and identification of mRNAs, microRNAs and protein components of ribonucleoprotein complexes from cell extracts. Nat Protoc (2006) 1:302–7. doi: 10.1038/nprot.2006.47 17406249

[53] Gagliardi M Matarazzo MR. RIP: RNA immunoprecipitation. Methods Mol Biol (2016) 1480:73–86. doi: 10.1007/978-1-4939-6380-5_7 27659976

[54] Tarallo R Giurato G Bruno G Ravo M Rizzo F Salvati A. The nuclear receptor ERβ engages AGO2 in regulation of gene transcription, RNA splicing and RISC loading. Genome Biol (2017) 18:189. doi: 10.1186/s13059-017-1321-0 29017520 PMC5634881

[55] Andrews S. FastQC: a quality control tool for high throughput sequence data (2010). Available at: http://www.bioinformatics.babraham.ac.uk/projects/fastqc.

[56] Martin M. Cutadapt removes adapter sequences from high-throughput sequencing reads. EMBnet J (2011) 17:3. doi: 10.14806/ej.17.1.200

[57] Dobin A Davis CA Schlesinger F Drenkow J Zaleski C Jha S. STAR: ultrafast universal RNA-seq aligner. Bioinformatics (2013) 29:15–21. doi: 10.1093/bioinformatics/bts635 23104886 PMC3530905

[58] Liao Y Smyth GK Shi W. featureCounts: an efficient general purpose program for assigning sequence reads to genomic features. Bioinformatics (2014) 30:923–30. doi: 10.1093/bioinformatics/btt656 24227677

[59] Love MI Huber W Anders S. Moderated estimation of fold change and dispersion for RNA-seq data with DESeq2. Genome Biol (2014) 15:550. doi: 10.1186/s13059-014-0550-8 25516281 PMC4302049

[60] Li B Dewey CN. RSEM: accurate transcript quantification from RNA-seq data with or without a reference genome. BMC Bioinf (2011) 12:323. doi: 10.1186/1471-2105-12-323 PMC3163565 21816040

[61] Available at: https://www.r-project.org/.

[62] Agostini F Zanzoni A Klus P Marchese D Cirillo D Tartaglia GG. catRAPID omics: a web server for large-scale prediction of protein-RNA interactions. Bioinformatics (2013) 29:2928–30. doi: 10.1093/bioinformatics/btt495 PMC3810848 23975767

[63] Polishchuk M Paz I Kohen R Mesika R Yakhini Z Mandel-Gutfreund Y. A combined sequence and structure based method for discovering enriched motifs in RNA from *in vivo* binding data. Methods (2017) 118-119:73–81. doi: 10.1016/j.ymeth.2017.03.003 28274760

[64] Polishchuk M Paz I Yakhini Z Mandel-Gutfreund Y. SMARTIV: combined sequence and structure *de-novo* motif discovery for *in-vivo* RNA binding data. Nucleic Acids Res (2018) 46:W221–w228. doi: 10.1093/nar/gky453 29800452 PMC6030986

[65] Bailey TL Boden M Buske FA Frith M Grant CE Clementi L. MEME SUITE: tools for motif discovery and searching. Nucleic Acids Res (2009) 37:W202–8. doi: 10.1093/nar/gkp335 PMC2703892 19458158

[66] Panda AC Martindale JL Gorospe M. Affinity pulldown of biotinylated RNA for detection of protein-RNA complexes. Bio Protoc (2016) 6:e2062. doi: 10.21769/BioProtoc.2062 PMC5431594 28516119

[67] Puca AA Lopardo V Montella F Di Pietro P Cesselli D Rolle IG. The longevity-associated variant of BPIFB4 reduces senescence in glioma cells and in patients’ lymphocytes favoring chemotherapy efficacy. Cells (2022) 11:294. doi: 10.3390/cells11020294 35053408 PMC8774353

[68] Théry C Ostrowski M Segura E. Membrane vesicles as conveyors of immune responses. Nat Rev Immunol (2009) 9:581–93. doi: 10.1038/nri2567 19498381

[69] Zaborowski MP Balaj L Breakefield XO Lai CP. Extracellular vesicles: composition, biological relevance, and methods of study. Bioscience (2015) 65:783–97. doi: 10.1093/biosci/biv084 PMC4776721 26955082

[70] Palmieri V Lucchetti D Gatto I Maiorana A Marcantoni M Maulucci G. Dynamic light scattering for the characterization and counting of extracellular vesicles: a powerful noninvasive tool. J Nanopart Res (2014) 16:2583. doi: 10.1007/s11051-014-2583-z

[71] Kramer A Stathopoulos V Girolami M Radde N. MCMC_CLIB-an advanced MCMC sampling package for ODE models. Bioinformatics (2014) 30:2991–2. doi: 10.1093/bioinformatics/btu429 25005749

[72] Subramanian A Tamayo P Mootha VK Mukherjee S Ebert BL Gillette MA. Gene set enrichment analysis: a knowledge-based approach for interpreting genome-wide expression profiles. Proc Natl Acad Sci U.S.A. (2005) 102:15545–50. doi: 10.1073/pnas.0506580102 PMC1239896 16199517

[73] Bowerman KL Rehman SF Vaughan A Lachner N Budden KF Kim RY. Disease-associated gut microbiome and metabolome changes in patients with chronic obstructive pulmonary disease. Nat Commun (2020) 11:5886. doi: 10.1038/s41467-020-19701-0 33208745 PMC7676259

[74] Kirkham PA Caramori G Casolari P Papi AA Edwards M Shamji B. Oxidative stress-induced antibodies to carbonyl-modified protein correlate with severity of chronic obstructive pulmonary disease. Am J Respir Crit Care Med (2011) 184:796–802. doi: 10.1164/rccm.201010-1605OC 21965015 PMC3398415

[75] Marwick JA Caramori G Casolari P Mazzoni F Kirkham PA Adcock IM. A role for phosphoinositol 3-kinase delta in the impairment of glucocorticoid responsiveness in patients with chronic obstructive pulmonary disease. J Allergy Clin Immunol (2010) 125:1146–53. doi: 10.1016/j.jaci.2010.02.003 20381852

[76] Howe EA Sinha R Schlauch D Quackenbush J. RNA-Seq analysis in MeV. Bioinform (2011) 27:3209–10. doi: 10.1093/bioinformatics/btr490 PMC3208390 21976420

[77] Saeed AI Sharov V White J Li J Liang W Bhagabati N. TM4: a free, open-source system for microarray data management and analysis. Biotechniques (2003) 34:374–8. doi: 10.2144/03342mt01 12613259

[78] Hänzelmann S Castelo R Guinney J. GSVA: gene set variation analysis for microarray and RNA-seq data. BMC Bioinf (2013) 14:7. doi: 10.1186/1471-2105-14-7 PMC3618321 23323831

[79] Carolan BJ Heguy A Harvey BG Leopold PL Ferris B Crystal RG. Up-regulation of expression of the ubiquitin carboxyl-terminal hydrolase L1 gene in human airway epithelium of cigarette smokers. Cancer Res (2006) 66:10729–40. doi: 10.1158/0008-5472.can-06-2224 17108109

[80] Tiotiu A Zounemat Kermani N Badi Y Pavlidis S Hansbro PM Guo YK. Sputum macrophage diversity and activation in asthma: role of severity and inflammatory phenotype. Allergy (2021) 76:775–88. doi: 10.1111/all.14535 32740964

[81] Hekking PP Loza MJ Pavlidis S de Meulder B Lefaudeux D Baribaud F. Pathway discovery using transcriptomic profiles in adult-onset severe asthma. J Allergy Clin Immunol (2018) 141:1280–90. doi: 10.1016/j.jaci.2017.06.037 28756296

[82] Johansen MD Mahbub RM Idrees S Nguyen DH Miemczyk S Pathinayake P. Increased SARS-CoV-2 infection, protease, and inflammatory responses in chronic obstructive pulmonary disease primary bronchial epithelial cells defined with single-cell RNA sequencing. Am J Respir Crit Care Med (2022) 206:712–29. doi: 10.1164/rccm.202108-1901OC PMC9799113 35549656

[83] Available at: https://www.genecards.org/cgi-bin/carddisp.pl?gene=PRR36,PG-GPPPAAa.

[84] Attanasio M Uhlenhaut NH Sousa VH O’Toole JF Otto E Anlag K. Loss of GLIS2 causes nephronophthisis in humans and mice by increased apoptosis and fibrosis. Nat Genet (2007) 39:1018–24. doi: 10.1038/ng2072 17618285

[85] Jetten AM. GLIS1-3 transcription factors: critical roles in the regulation of multiple physiological processes and diseases. Cell Mol Life Sci (2018) 75:3473–94. doi: 10.1007/s00018-018-2841-9 PMC6123274 29779043

[86] Available at: https://www.genecards.org/cgi-bin/carddisp.pl?gene=ZNF385A,ZAGZAPZAAAa.

[87] Murphy M Chatterjee SS Jain S Katari M DasGupta R. TCF7L1 modulates colorectal cancer growth by inhibiting expression of the tumor-suppressor gene EPHB3. Sci Rep (2016) 6:28299. doi: 10.1038/srep28299 27333864 PMC4917863

[88] Shan J Shen J Wu M Zhou H Feng J Yao C. Tcf7l1 acts as a suppressor for the self-renewal of liver cancer stem cells and is regulated by IGF/MEK/ERK signaling independent of β-catenin. Stem Cells (2019) 37:1389–400. doi: 10.1002/stem.3063 31322782

[89] Sierra RA Hoverter NP Ramirez RN Vuong LM Mortazavi A Merrill BJ. TCF7L1 suppresses primitive streak gene expression to support human embryonic stem cell pluripotency. Development (2018) 145:dev161075. doi: 10.1242/dev.161075 29361574 PMC5869011

[90] Kogure A Shiratori I Wang J Lanier LL Arase H. PANP is a novel O-glycosylated PILRα ligand expressed in neural tissues. Biochem Biophys Res Commun (2011) 405:428–33. doi: 10.1016/j.bbrc.2011.01.047 PMC4089865 21241660

[91] Available at: https://www.genecards.org/cgi-bin/carddisp.pl?gene=MBD6,MGMPMAAa.

[92] Milara J Díaz-Platas L Contreras S Ribera P Roger I Ballester B. MUC1 deficiency mediates corticosteroid resistance in chronic obstructive pulmonary disease. Respir Res (2018) 19:226. doi: 10.1186/s12931-018-0927-4 30458870 PMC6247701

[93] Nath S Mukherjee P. MUC1: a multifaceted oncoprotein with a key role in cancer progression. Trends Mol Med (2014) 20:332–42. doi: 10.1016/j.molmed.2014.02.007 PMC5500204 24667139

[94] Co M Anderson AG Konopka G. FOXP transcription factors in vertebrate brain development, function, and disorders. Wiley Interdiscip Rev Dev Biol (2020) 9:e375. doi: 10.1002/wdev.375 31999079 PMC8286808

[95] Kim JH Hwang J Jung JH Lee HJ Lee DY Kim SH. Molecular networks of FOXP family: dual biologic functions, interplay with other molecules and clinical implications in cancer progression. Mol Cancer (2019) 18:180. doi: 10.1186/s12943-019-1110-3 31815635 PMC6900861

[96] Burchfield JS Li Q Wang HY Wang RF. JMJD3 as an epigenetic regulator in development and disease. Int J Biochem Cell Biol (2015) 67:148–57. doi: 10.1016/j.biocel.2015.07.006 PMC4564304 26193001

[97] Wijayatunge R Liu F Shpargel KB Wayne NJ Chan U Boua JV. The histone demethylase Kdm6b regulates a mature gene expression program in differentiating cerebellar granule neurons. Mol Cell Neurosci (2018) 87:4–17. doi: 10.1016/j.mcn.2017.11.005 29254825 PMC5828961

[98] Available at: https://www.genecards.org/cgi-bin/carddisp.pl?gene=FBRSL1,FGFPFAAa.

[99] Available at: https://www.genecards.org/cgi-bin/carddisp.pl?gene=C1orf226&keywords=C1orf226.

[100] Südhof TC. Neurotransmitter release: the last millisecond in the life of a synaptic vesicle. Neuron (2013) 80:675–90. doi: 10.1016/j.neuron.2013.10.022 PMC3866025 24183019

[101] Parra-Damas A Rubió-Ferrarons L Shen J Saura CA. CRTC1 mediates preferential transcription at neuronal activity-regulated CRE/TATA promoters. Sci Rep (2017) 7:18004. doi: 10.1038/s41598-017-18215-y 29269871 PMC5740062

[102] Kaehler C Isensee J Nonhoff U Terrey M Hucho T Lehrach H. Ataxin-2-like is a regulator of stress granules and processing bodies. PloS One (2012) 7:e50134. doi: 10.1371/journal.pone.0050134 23209657 PMC3507954

[103] Li YY Wu C Shah SS Chen SM Wangpaichitr M Kuo MT. Degradation of AMPK-α1 sensitizes BRAF inhibitor-resistant melanoma cells to arginine deprivation. Mol Oncol (2017) 11:1806–25. doi: 10.1002/1878-0261.12151 PMC5709618 29094484

[104] Rong Z Wang A Li Z Ren Y Cheng L Li Y. IL-17RD (Sef or IL-17RLM) interacts with IL-17 receptor and mediates IL-17 signaling. Cell Res (2009) 19:208–15. doi: 10.1038/cr.2008.320 PMC4603938 19079364

[105] Mellett M Atzei P Horgan A Hams E Floss T Wurst W. Orphan receptor IL-17RD tunes IL-17A signalling and is required for neutrophilia. Nat Commun (2012) 3:1119. doi: 10.1038/ncomms2127 23047677

[106] . Available at: https://www.genecards.org/cgi-bin/carddisp.pl?gene=KIAA1522&keywords=KIAA1522.

[107] Allen CE Mak CH Wu LC. The kappa b transcriptional enhancer motif and signal sequences of V(D)J recombination are targets for the zinc finger protein HIVEP3/KRC: a site selection amplification binding study. BMC Immunol (2002) 3:10. doi: 10.1186/1471-2172-3-10 12193271 PMC122077

[108] Jones DC Wein MN Oukka M Hofstaetter JG Glimcher MJ Glimcher LH. Regulation of adult bone mass by the zinc finger adapter protein schnurri-3. Science (2006) 312:1223–7. doi: 10.1126/science.1126313 16728642

[109] Noren Hooten N Martin-Montalvo A Dluzen DF Zhang Y Bernier M Zonderman AB. Metformin-mediated increase in DICER1 regulates microRNA expression and cellular senescence. Aging Cell (2016) 15:572–81. doi: 10.1111/acel.12469 PMC4854919 26990999

[110] Kedar VP Zucconi BE Wilson GM Blackshear PJ. Direct binding of specific AUF1 isoforms to tandem zinc finger domains of tristetraprolin (TTP) family proteins. J Biol Chem (2012) 287:5459–71. doi: 10.1074/jbc.M111.312652 PMC3325588 22203679

[111] Wagner BJ DeMaria CT Sun Y Wilson GM Brewer G. Structure and genomic organization of the human AUF1 gene: alternative pre-mRNA splicing generates four protein isoforms. Genomics (1998) 48:195–202. doi: 10.1006/geno.1997.5142 9521873

[112] Zucconi BE Wilson GM. Assembly of functional ribonucleoprotein complexes by AU-rich element RNA-binding protein 1 (AUF1) requires base-dependent and -independent RNA contacts. J Biol Chem (2013) 288:28034–48. doi: 10.1074/jbc.M113.489559 PMC3784716 23940053

[113] Wilson GM Lu J Sutphen K Sun Y Huynh Y Brewer G. Regulation of a + U-rich element-directed mRNA turnover involving reversible phosphorylation of AUF1. J Biol Chem (2003) 278:33029–38. doi: 10.1074/jbc.M305772200 12819195

[114] Paschoud S Dogar AM Kuntz C Grisoni-Neupert B Richman L Kühn LC. Destabilization of interleukin-6 mRNA requires a putative RNA stem-loop structure, an AU-rich element, and the RNA-binding protein AUF1. Mol Cell Biol (2006) 26:8228–41. doi: 10.1128/mcb.01155-06 PMC1636780 16954375

[115] Lal A Mazan-Mamczarz K Kawai T Yang X Martindale JL Gorospe M. Concurrent versus individual binding of HuR and AUF1 to common labile target mRNAs. EMBO J (2004) 23:3092–102. doi: 10.1038/sj.emboj.7600305 PMC514922 15257295

[116] Tamamori-Adachi M Koga A Susa T Fujii H Tsuchiya M Okinaga H. DNA Damage response induced by etoposide promotes steroidogenesis *via* GADD45A in cultured adrenal cells. Sci Rep (2018) 8:9636. doi: 10.1038/s41598-018-27938-5 29941883 PMC6018231

[117] Barnes PJ Baker J Donnelly LE. Cellular senescence as a mechanism and target in chronic lung diseases. Am J Respir Crit Care Med (2019) 200:556–64. doi: 10.1164/rccm.201810-1975TR 30860857

[118] Laroia G Cuesta R Brewer G Schneider RJ. Control of mRNA decay by heat shock-ubiquitin-proteasome pathway. Science (1999) 284:499–502. doi: 10.1126/science.284.5413.499 10205060

[119] Laroia G Sarkar B Schneider RJ. Ubiquitin-dependent mechanism regulates rapid turnover of AU-rich cytokine mRNAs. Proc Natl Acad Sci U.S.A. (2002) 99:1842–6. doi: 10.1073/pnas.042575699 PMC122281 11842200

[120] Ito T Sun L Bevan MA Crooks RM. Comparison of nanoparticle size and electrophoretic mobility measurements using a carbon-nanotube-based coulter counter, dynamic light scattering, transmission electron microscopy, and phase analysis light scattering. Langmuir (2004) 20:6940–5. doi: 10.1021/la049524t 15274607

[121] Souza TGF Ciminelli VST Mohallem NDS. A comparison of TEM and DLS methods to characterize size distribution of ceramic nanoparticles. J Phys: Conf Ser (2016) 2016:733 012039. doi: 10.1088/1742-6596/733/1/012039

[122] Machado RD Southgate L Eichstaedt CA Aldred MA Austin ED Best DH. Pulmonary arterial hypertension: a current perspective on established and emerging molecular genetic defects. Hum Mutat (2015) 36:1113–27. doi: 10.1002/humu.22904 PMC4822159 26387786

[123] Kim CW Song H Kumar S Nam D Kwon HS Chang KH. Anti-inflammatory and antiatherogenic role of BMP receptor II in endothelial cells. Arterioscler Thromb Vasc Biol (2013) 33:1350–9. doi: 10.1161/atvbaha.112.300287 PMC3758923 23559633

[124] Bossé Y. Updates on the COPD gene list. Int J Chron Obstruct Pulmon Dis (2012) 7:607–31. doi: 10.2147/copd.s35294 PMC3459654 23055711

[125] Wang J Zhang C Zhang Z Zheng Z Sun D Yang Q. A functional variant rs6435156C > T in BMPR2 is associated with increased risk of chronic obstructive pulmonary disease (COPD) in southern Chinese population. EBioMedicine (2016) 5:167–74. doi: 10.1016/j.ebiom.2016.02.004 PMC4816816 27077124

[126] Llinàs L Peinado VI Ramon Goñi J Rabinovich R Pizarro S Rodriguez-Roisin R. Similar gene expression profiles in smokers and patients with moderate COPD. Pulm Pharmacol Ther (2011) 24:32–41. doi: 10.1016/j.pupt.2010.10.010 20970515

[127] Barak O Lazzaro MA Lane WS Speicher DW Picketts DJ Shiekhattar R. Isolation of human NURF: a regulator of engrailed gene expression. EMBO J (2003) 22:6089–100. doi: 10.1093/emboj/cdg582 PMC275440 14609955

[128] Oppikofer M Bai T Gan Y Haley B Liu P Sandoval W. Expansion of the ISWI chromatin remodeler family with new active complexes. EMBO Rep (2017) 18:1697–706. doi: 10.15252/embr.201744011 PMC5623870 28801535

[129] Imbriano C Bolognese F Gurtner A Piaggio G Mantovani R. HSP-CBF is an NF-y-dependent coactivator of the heat shock promoters CCAAT boxes. J Biol Chem (2001) 276:26332–9. doi: 10.1074/jbc.M101553200 11306579

[130] Castello A Fischer B Eichelbaum K Horos R Beckmann BM Strein C. Insights into RNA biology from an atlas of mammalian mRNA-binding proteins. Cell (2012) 149:1393–406. doi: 10.1016/j.cell.2012.04.031 22658674

[131] Pilarowski GO Vernon HJ Applegate CD Boukas L Cho MT Gurnett CA. Missense variants in the chromatin remodeler CHD1 are associated with neurodevelopmental disability. J Med Genet (2018) 55:561–6. doi: 10.1136/jmedgenet-2017-104759 PMC5834353 28866611

[132] Tai HH Geisterfer M Bell JC Moniwa M Davie JR Boucher L. CHD1 associates with NCoR and histone deacetylase as well as with RNA splicing proteins. Biochem Biophys Res Commun (2003) 308:170–6. doi: 10.1016/s0006-291x(03)01354-8 12890497

[133] Zhou J Li J Serafim RB Ketchum S Ferreira CG Liu JC. Human CHD1 is required for early DNA-damage signaling and is uniquely regulated by its n terminus. Nucleic Acids Res (2018) 46:3891–905. doi: 10.1093/nar/gky128 PMC5934646 29529298

[134] Sims RJ 3rd Millhouse S Chen CF Lewis BA Erdjument-Bromage H Tempst P. Recognition of trimethylated histone H3 lysine 4 facilitates the recruitment of transcription postinitiation factors and pre-mRNA splicing. Mol Cell (2007) 28:665–76. doi: 10.1016/j.molcel.2007.11.010 PMC2276655 18042460

[135] Iinuma T Shiga A Nakamoto K O’Brien MB Aridor M Arimitsu N. Mammalian Sec16/p250 plays a role in membrane traffic from the endoplasmic reticulum. J Biol Chem (2007) 282:17632–9. doi: 10.1074/jbc.M611237200 17428803

[136] Booy EP Meier M Okun N Novakowski SK Xiong S Stetefeld J. The RNA helicase RHAU (DHX36) unwinds a G4-quadruplex in human telomerase RNA and promotes the formation of the P1 helix template boundary. Nucleic Acids Res (2012) 40:4110–24. doi: 10.1093/nar/gkr1306 PMC3351167 22238380

[137] Giri B Smaldino PJ Thys RG Creacy SD Routh ED Hantgan RR. G4 resolvase 1 tightly binds and unwinds unimolecular G4-DNA. Nucleic Acids Res (2011) 39:7161–78. doi: 10.1093/nar/gkr234 PMC3167620 21586581

[138] Vaughn JP Creacy SD Routh ED Joyner-Butt C Jenkins GS Pauli S. The DEXH protein product of the DHX36 gene is the major source of tetramolecular quadruplex G4-DNA resolving activity in HeLa cell lysates. J Biol Chem (2005) 280:38117–20. doi: 10.1074/jbc.C500348200 16150737

[139] Newman M Sfaxi R Saha A Monchaud D Teulade-Fichou MP Vagner S. The G-Quadruplex-Specific RNA helicase DHX36 regulates p53 pre-mRNA 3'-end processing following UV-induced DNA damage. J Mol Biol (2017) 429:3121–31. doi: 10.1016/j.jmb.2016.11.033 27940037

[140] Huang W Smaldino PJ Zhang Q Miller LD Cao P Stadelman K. Yin yang 1 contains G-quadruplex structures in its promoter and 5’-UTR and its expression is modulated by G4 resolvase 1. Nucleic Acids Res (2012) 40:1033–49. doi: 10.1093/nar/gkr849 PMC3273823 21993297

[141] Iwamoto F Stadler M Chalupníková K Oakeley E Nagamine Y. Transcription-dependent nucleolar cap localization and possible nuclear function of DExH RNA helicase RHAU. Exp Cell Res (2008) 314:1378–91. doi: 10.1016/j.yexcr.2008.01.006 18279852

[142] Tran H Schilling M Wirbelauer C Hess D Nagamine Y. Facilitation of mRNA deadenylation and decay by the exosome-bound, DExH protein RHAU. Mol Cell (2004) 13:101–11. doi: 10.1016/s1097-2765(03)00481-7 14731398

[143] Antcliff A McCullough LD Tsvetkov AS. G-Quadruplexes and the DNA/RNA helicase DHX36 in health, disease, and aging. Aging (Albany NY) (2021) 13:25578–87. doi: 10.18632/aging.203738 PMC8714159 34862880

[144] Sanders MA Ampasala D Basson MD. DOCK5 and DOCK1 regulate caco-2 intestinal epithelial cell spreading and migration on collagen IV. J Biol Chem (2009) 284:27–35. doi: 10.1074/jbc.M808010200 19004829 PMC2610524

[145] Rossi M Duan S Jeong YT Horn M Saraf A Florens L. Regulation of the CRL4(Cdt2) ubiquitin ligase and cell-cycle exit by the SCF(Fbxo11) ubiquitin ligase. Mol Cell (2013) 49:1159–66. doi: 10.1016/j.molcel.2013.02.004 PMC3624904 23478441

[146] Kane MS Diamonstein CJ Hauser N Deeken JF Niederhuber JE Vilboux T. Endosomal trafficking defects in patient cells with KIAA1109 biallelic variants. Genes Dis (2019) 6:56–67. doi: 10.1016/j.gendis.2018.12.004 30906834 PMC6411657

[147] Jeng EE Bhadkamkar V Ibe NU Gause H Jiang L Chan J. Systematic identification of host cell regulators of legionella pneumophila pathogenesis using a genome-wide CRISPR screen. Cell Host Microbe (2019) 26:551–563.e6. doi: 10.1016/j.chom.2019.08.017 31540829 PMC6800164

[148] Jäkel S Görlich D. Importin beta, transportin, RanBP5 and RanBP7 mediate nuclear import of ribosomal proteins in mammalian cells. EMBO J (1998) 17:4491–502. doi: 10.1093/emboj/17.15.4491 PMC1170780 9687515

[149] Petit MM Fradelizi J Golsteyn RM Ayoubi TA Menichi B Louvard D. LPP, an actin cytoskeleton protein related to zyxin, harbors a nuclear export signal and transcriptional activation capacity. Mol Biol Cell (2000) 11:117–29. doi: 10.1091/mbc.11.1.117 PMC14761 10637295

[150] Kakinuma T Ichikawa H Tsukada Y Nakamura T Toh BH. Interaction between p230 and MACF1 is associated with transport of a glycosyl phosphatidyl inositol-anchored protein from the golgi to the cell periphery. Exp Cell Res (2004) 298:388–98. doi: 10.1016/j.yexcr.2004.04.047 15265687

[151] Zaoui K Benseddik K Daou P Salaün D Badache A. ErbB2 receptor controls microtubule capture by recruiting ACF7 to the plasma membrane of migrating cells. Proc Natl Acad Sci U.S.A. (2010) 107:18517–22. doi: 10.1073/pnas.1000975107 PMC2972954 20937854

[152] Jung EJ Liu G Zhou W Chen X. Myosin VI is a mediator of the p53-dependent cell survival pathway. Mol Cell Biol (2006) 26:2175–86. doi: 10.1128/mcb.26.6.2175-2186.2006 PMC1430268 16507995

[153] Wells AL Lin AW Chen LQ Safer D Cain SM Hasson T. Myosin VI is an actin-based motor that moves backwards. Nature (1999) 401:505–8. doi: 10.1038/46835 10519557

[154] Mozzi A Forcella M Riva A Difrancesco C Molinari F Martin V. NEU3 activity enhances EGFR activation without affecting EGFR expression and acts on its sialylation levels. Glycobiology (2015) 25:855–68. doi: 10.1093/glycob/cwv026 25922362

[155] Wada T Hata K Yamaguchi K Shiozaki K Koseki K Moriya S. A crucial role of plasma membrane-associated sialidase in the survival of human cancer cells. Oncogene (2007) 26:2483–90. doi: 10.1038/sj.onc.1210341 17334392

[156] Kadoch C Crabtree GR. Mammalian SWI/SNF chromatin remodeling complexes and cancer: mechanistic insights gained from human genomics. Sci Adv (2015) 1:e1500447. doi: 10.1126/sciadv.1500447 26601204 PMC4640607

[157] Varela I Tarpey P Raine K Huang D Ong CK Stephens P. Exome sequencing identifies frequent mutation of the SWI/SNF complex gene PBRM1 in renal carcinoma. Nature (2011) 469:539–42. doi: 10.1038/nature09639 PMC3030920 21248752

[158] Kanayama HO Tamura T Ugai S Kagawa S Tanahashi N Yoshimura T. Demonstration that a human 26S proteolytic complex consists of a proteasome and multiple associated protein components and hydrolyzes ATP and ubiquitin-ligated proteins by closely linked mechanisms. Eur J Biochem (1992) 206:567–78. doi: 10.1111/j.1432-1033.1992.tb16961.x 1317798

[159] Neilson DE Adams MD Orr CM Schelling DK Eiben RM Kerr DS. Infection-triggered familial or recurrent cases of acute necrotizing encephalopathy caused by mutations in a component of the nuclear pore, RANBP2. Am J Hum Genet (2009) 84:44–51. doi: 10.1016/j.ajhg.2008.12.009 19118815 PMC2668029

[160] De Amicis A Piane M Ferrari F Fanciulli M Delia D Chessa L. Role of senataxin in DNA damage and telomeric stability. DNA Repair (Amst) (2011) 10:199–209. doi: 10.1016/j.dnarep.2010.10.012 21112256

[161] Richard P Feng S Manley JL. A SUMO-dependent interaction between senataxin and the exosome, disrupted in the neurodegenerative disease AOA2, targets the exosome to sites of transcription-induced DNA damage. Genes Dev (2013) 27:2227–32. doi: 10.1101/gad.224923.113 PMC3814643 24105744

[162] Suraweera A Becherel OJ Chen P Rundle N Woods R Nakamura J. Senataxin, defective in ataxia oculomotor apraxia type 2, is involved in the defense against oxidative DNA damage. J Cell Biol (2007) 177:969–79. doi: 10.1083/jcb.200701042 PMC2064358 17562789

[163] Suraweera A Lim Y Woods R Birrell GW Nasim T Becherel OJ. Functional role for senataxin, defective in ataxia oculomotor apraxia type 2, in transcriptional regulation. Hum Mol Genet (2009) 18:3384–96. doi: 10.1093/hmg/ddp278 19515850

[164] Haselbach D Komarov I Agafonov DE Hartmuth K Graf B Dybkov O. Structure and conformational dynamics of the human spliceosomal b(act) complex. Cell (2018) 172:454–64.e11. doi: 10.1016/j.cell.2018.01.010 29361316

[165] Zhan X Yan C Zhang X Lei J Shi Y. Structures of the human pre-catalytic spliceosome and its precursor spliceosome. Cell Res (2018) 28:1129–40. doi: 10.1038/s41422-018-0094-7 PMC6274647 30315277

[166] Zhang X Yan C Zhan X Li L Lei J Shi Y. Structure of the human activated spliceosome in three conformational states. Cell Res (2018) 28:307–22. doi: 10.1038/cr.2018.14 PMC5835773 29360106

[167] Sumara I Vorlaufer E Gieffers C Peters BH Peters JM. Characterization of vertebrate cohesin complexes and their regulation in prophase. J Cell Biol (2000) 151:749–62. doi: 10.1083/jcb.151.4.749 PMC2169443 11076961

[168] Terret ME Sherwood R Rahman S Qin J Jallepalli PV. Cohesin acetylation speeds the replication fork. Nature (2009) 462:231–4. doi: 10.1038/nature08550 PMC2777716 19907496

[169] Ahn EY DeKelver RC Lo MC Nguyen TA Matsuura S Boyapati A. SON controls cell-cycle progression by coordinated regulation of RNA splicing. Mol Cell (2011) 42:185–98. doi: 10.1016/j.molcel.2011.03.014 PMC3137374 21504830

[170] Huen MS Sy SM Leung KM Ching YP Tipoe GL Man C. SON is a spliceosome-associated factor required for mitotic progression. Cell Cycle (2010) 9:2679–85. doi: 10.4161/cc.9.13.12151 PMC3040851 20581448

[171] Gindele JA Kiechle T Benediktus K Birk G Brendel M Heinemann F. Intermittent exposure to whole cigarette smoke alters the differentiation of primary small airway epithelial cells in the air-liquid interface culture. Sci Rep (2020) 10:6257. doi: 10.1038/s41598-020-63345-5 32277131 PMC7148343

[172] Bodas M Moore AR Subramaniyan B Georgescu C Wren JD Freeman WM. Cigarette smoke activates NOTCH3 to promote goblet cell differentiation in human airway epithelial cells. Am J Respir Cell Mol Biol (2021) 64:426–40. doi: 10.1165/rcmb.2020-0302OC PMC8008804 33444514

[173] Ito K Ito M Elliott WM Cosio B Caramori G Kon OM. Decreased histone deacetylase activity in chronic obstructive pulmonary disease. N Engl J Med (2005) 352:1967–76. doi: 10.1056/NEJMoa041892 15888697

[174] To M Swallow EB Akashi K Haruki K Natanek SA Polkey MI. Reduced HDAC2 in skeletal muscle of COPD patients. Respir Res (2017) 18:99. doi: 10.1186/s12931-017-0588-8 28526090 PMC5438490

[175] Gao W Li L Wang Y Zhang S Adcock IM Barnes PJ. Bronchial epithelial cells: the key effector cells in the pathogenesis of chronic obstructive pulmonary disease? Respirology (2015) 20:722–9. doi: 10.1111/resp.12542 25868842

[176] Hsu AC Dua K Starkey MR Haw TJ Nair PM Nichol K. MicroRNA-125a and -b inhibit A20 and MAVS to promote inflammation and impair antiviral response in COPD. JCI Insight (2017) 2:e90443. doi: 10.1172/jci.insight.90443 28405612 PMC5374076

[177] Marchese D de Groot NS Lorenzo Gotor N Livi CM Tartaglia GG. Advances in the characterization of RNA-binding proteins. Wiley Interdiscip Rev RNA (2016) 7:793–810. doi: 10.1002/wrna.1378 27503141 PMC5113702

[178] Mazan-Mamczarz K Kuwano Y Zhan M White EJ Martindale JL Lal A. Identification of a signature motif in target mRNAs of RNA-binding protein AUF1. Nucleic Acids Res (2009) 37:204–14. doi: 10.1093/nar/gkn929 PMC2615618 19033365

[179] White EJ Matsangos AE Wilson GM. AUF1 regulation of coding and noncoding RNA. Wiley Interdiscip Rev RNA (2017) 8(2):10.1002/wrna.1393. doi: 10.1002/wrna.1393 PMC5315606 27620010

[180] Louis IV SCMC-AR. Sequence elements. In: Uchiumi F, editor. Gene expression and regulation in, IntechOpen MC-TFGAL (2018).

[181] Saha S Chakraborty A Bandyopadhyay SS. Stabilization of oncostatin-m mRNA by binding of nucleolin to a GC-rich element in its 3’UTR. J Cell Biochem (2016) 117:988–99. doi: 10.1002/jcb.25384 26399567

[182] Chakraborty A Mukherjee S Saha S De S Sengupta Bandyopadhyay S Biochem J. Phorbol-12-myristate-13-acetate-mediated stabilization of leukemia inhibitory factor (lif) mRNA: involvement of nucleolin and PCBP1. Biochem. J. (2017) 474:2349–63. doi: 10.1042/bcj20170051 28512205

[183] Litterman AJ Kageyama R Le Tonqueze O Zhao W Gagnon JD Goodarzi H. A massively parallel 3’ UTR reporter assay reveals relationships between nucleotide content, sequence conservation, and mRNA destabilization. Genome Res (2019) 29:896–906. doi: 10.1101/gr.242552.118 31152051 PMC6581050

[184] Yu H Sun Y Haycraft C Palanisamy V Kirkwood KL. MKP-1 regulates cytokine mRNA stability through selectively modulation subcellular translocation of AUF1. Cytokine (2011) 56:245–55. doi: 10.1016/j.cyto.2011.06.006 PMC3185122 21733716

[185] Li CJ Ning W Matthay MA Feghali-Bostwick CA Choi AM. MAPK pathway mediates EGR-1-HSP70-dependent cigarette smoke-induced chemokine production. Am J Physiol Lung Cell Mol Physiol (2007) 292:L1297–303. doi: 10.1152/ajplung.00194.2006 17494953

[186] Ning W Li CJ Kaminski N Feghali-Bostwick CA Alber SM Di YP. Comprehensive gene expression profiles reveal pathways related to the pathogenesis of chronic obstructive pulmonary disease. Proc Natl Acad Sci U.S.A. (2004) 101:14895–900. doi: 10.1073/pnas.0401168101 PMC522001 15469929

[187] Liao B Hu Y Brewer G. Competitive binding of AUF1 and TIAR to MYC mRNA controls its translation. Nat Struct Mol Biol (2007) 14:511–8. doi: 10.1038/nsmb1249 17486099

[188] Nair PM Starkey MR Haw TJ Liu G Collison AM Mattes J. Enhancing tristetraprolin activity reduces the severity of cigarette smoke-induced experimental chronic obstructive pulmonary disease. Clin Transl Immunol (2019) 8:e01084. doi: 10.1002/cti2.1084 PMC6946917 31921419

[189] Fragkouli A Koukouraki P Vlachos IS Paraskevopoulou MD Hatzigeorgiou AG Doxakis E. Neuronal ELAVL proteins utilize AUF-1 as a co-partner to induce neuron-specific alternative splicing of APP. Sci Rep (2017) 7:44507. doi: 10.1038/srep44507 28291226 PMC5349543

[190] Hansel C Jendrossek V Klein D. Cellular senescence in the lung: the central role of senescent epithelial cells. Int J Mol Sci (2020) 21:3279. doi: 10.3390/ijms21093279 32384619 PMC7247355

[191] Tsuji T Aoshiba K Nagai A. Cigarette smoke induces senescence in alveolar epithelial cells. Am J Respir Cell Mol Biol (2004) 31:643–9. doi: 10.1165/rcmb.2003-0290OC 15333326

[192] Chowdhury S Dijkhuis A Steiert S Lutter R. IL-17 attenuates degradation of ARE-mRNAs by changing the cooperation between AU-binding proteins and microRNA16. PloS Genet (2013) 9:e1003747. doi: 10.1371/journal.pgen.1003747 24086143 PMC3784493

[193] Wang W Yang X Cristofalo VJ Holbrook NJ Gorospe M. Loss of HuR is linked to reduced expression of proliferative genes during replicative senescence. Mol Cell Biol (2001) 21:5889–98. doi: 10.1128/mcb.21.17.5889-5898.2001 PMC87308 11486028

[194] Kawagishi H Hashimoto M Nakamura H Tsugawa T Watanabe A Kontoyiannis DL. HuR maintains a replicative life span by repressing the ARF tumor suppressor. Mol Cell Biol (2013) 33:1886–900. doi: 10.1128/mcb.01277-12 PMC3647966 23508105

[195] Lee JH Jung M Hong J Kim MK Chung IK. Loss of RNA-binding protein HuR facilitates cellular senescence through posttranscriptional regulation of TIN2 mRNA. Nucleic Acids Res (2018) 46:4271–85. doi: 10.1093/nar/gky223 PMC5934620 29584879

[196] Yi J Chang N Liu X Guo G Xue L Tong T. Reduced nuclear export of HuR mRNA by HuR is linked to the loss of HuR in replicative senescence. Nucleic Acids Res (2010) 38:1547–58. doi: 10.1093/nar/gkp1114 PMC2836555 20007147

[197] Ryu S Jung M Kim C Kang H Han S Cha S. Loss of RNA binding protein HuD facilitates the production of the senescence-associated secretory phenotype. Cell Death Dis (2022) 13:329. doi: 10.1038/s41419-022-04792-y 35411051 PMC9001635

[198] Majumder M House R Palanisamy N Qie S Day TA Neskey D. RNA-Binding protein FXR1 regulates p21 and TERC RNA to bypass p53-mediated cellular senescence in OSCC. PloS Genet (2016) 12:e1006306. doi: 10.1371/journal.pgen.1006306 27606879 PMC5015924

[199] Avolio R Inglés-Ferrándiz M Ciocia A Coll O Bonnin S Guitart T. Coordinated post-transcriptional control of oncogene-induced senescence by UNR/CSDE1. Cell Rep (2022) 38:110211. doi: 10.1016/j.celrep.2021.110211 35021076

[200] Tan DBA Armitage J Teo TH Ong NE Shin H Moodley YP. Elevated levels of circulating exosome in COPD patients are associated with systemic inflammation. Respir Med (2017) 132:261–4. doi: 10.1016/j.rmed.2017.04.014 28476471

[201] Wahlund CJE Eklund A Grunewald J Gabrielsson S. Pulmonary extracellular vesicles as mediators of local and systemic inflammation. Front Cell Dev Biol (2017) 5:39. doi: 10.3389/fcell.2017.00039 28491866 PMC5405144

[202] Gupta R Radicioni G Abdelwahab S Dang H Carpenter J Chua M. Intercellular communication between airway epithelial cells is mediated by exosome-like vesicles. Am J Respir Cell Mol Biol (2019) 60:209–20. doi: 10.1165/rcmb.2018-0156OC PMC6376407 30230353

[203] Purghè B Manfredi M Ragnoli B Baldanzi G Malerba M. Exosomes in chronic respiratory diseases. BioMed Pharmacother (2021) 144:112270. doi: 10.1016/j.biopha.2021.112270 34678722

[204] Trappe A Donnelly SC McNally P Coppinger JA. Role of extracellular vesicles in chronic lung disease. Thorax (2021) 76:1047–56. doi: 10.1136/thoraxjnl-2020-216370 PMC8461402 33712504

[205] Lässer C Shelke GV Yeri A Kim DK Crescitelli R Raimondo S. Two distinct extracellular RNA signatures released by a single cell type identified by microarray and next-generation sequencing. RNA Biol (2017) 14:58–72. doi: 10.1080/15476286.2016.1249092 27791479 PMC5270547

[206] Xiao H Ye X Vishwakarma V Preet R Dixon DA. CRC-Derived exosomes containing the RNA binding protein HuR promote lung cell proliferation by stabilizing c-myc mRNA. Cancer Biol Ther (2022) 23:139–49. doi: 10.1080/15384047.2022.2034455 PMC8824215 35130122

[207] Fujita Y Araya J Ito S Kobayashi K Kosaka N Yoshioka Y. Suppression of autophagy by extracellular vesicles promotes myofibroblast differentiation in COPD pathogenesis. J Extracell Vesicles (2015) 4:28388. doi: 10.3402/jev.v4.28388 26563733 PMC4643181

[208] Moon HG Cao Y Yang J Lee JH Choi HS Jin Y. Lung epithelial cell-derived extracellular vesicles activate macrophage-mediated inflammatory responses *via* ROCK1 pathway. Cell Death Dis (2015) 6:e2016. doi: 10.1038/cddis.2015.282 26658190 PMC4720875

[209] Yu Y Zhou Y Di C Zhao C Chen J Su W. Increased airway epithelial cell-derived exosomes activate macrophage-mediated allergic inflammation *via* CD100 shedding. J Cell Mol Med (2021) 25:8850–62. doi: 10.1111/jcmm.16843 PMC8435458 34414666

[210] Li P Wang Y Wang X Liu L Chen L. Identification of susceptible genes for chronic obstructive pulmonary disease with lung adenocarcinoma by weighted gene Co-expression network analysis. Onco Targets Ther (2021) 14:3625–34. doi: 10.2147/ott.s303544 PMC8187107 34113128

[211] D’Agostino VG Lal P Mantelli B Tiedje C Zucal C Thongon N. Dihydrotanshinone-I interferes with the RNA-binding activity of HuR affecting its post-transcriptional function. Sci Rep (2015) 5:16478. doi: 10.1038/srep16478 26553968 PMC4639722

[212] Della Volpe S Nasti R Queirolo M Unver MY Jumde VK Dömling A. Novel compounds targeting the RNA-binding protein HuR. structure-based design, synthesis, and interaction studies. ACS Med Chem Lett (2019) 10:615–20. doi: 10.1021/acsmedchemlett.8b00600 PMC6466816 30996806

[213] Wu P. Inhibition of RNA-binding proteins with small molecules. Nat Rev Chem (2020) 4:1–18. doi: 10.1038/s41570-020-0201-4 37127961

[214] Schultz CW Preet R Dhir T Dixon DA Brody JR. Understanding and targeting the disease-related RNA binding protein human antigen r (HuR). Wiley Interdiscip Rev RNA (2020) 11:e1581. doi: 10.1002/wrna.1581 31970930 PMC7482136

[215] Julio AR Backus KM. New approaches to target RNA binding proteins. Curr Opin Chem Biol (2021) 62:13–23. doi: 10.1016/j.cbpa.2020.12.006 33535093 PMC8823266

