# Supplementary material for: Expression of targets of the RNA-binding protein AUF-1 in human airway epithelium indicates its role in cellular senescence and inflammation
Source: Front Immunol. 2023 Jul 7;14:1192028. doi: 10.3389/fimmu.2023.1192028 (PMC10360199; doi:10.3389/fimmu.2023.1192028)
Supplement: Supplementary file 1 [file DataSheet_1.docx]

***Supplementary Material***

**Expression of targets of the RNA-binding protein AUF-1 in human airway epithelium indicates its role in cellular senescence and inflammation**

Ilaria Salvato^1, 2†^, Luca Ricciardi ^1,2†^, Jessica Dal Col^1^, Annunziata Nigro^1^, Giorgio Giurato^1^, Domenico Memoli^1^, Assunta Sellitto^1^, Erwin Pavel Lamparelli^1^, Maria Assunta Crescenzi^1^, Monica Vitale^1^, Alessandro Vatrella^1^, Francesco Nucera^2^, Paola Brun^3^, Federico Caicci^4^, Paola Dama^5^, Thomas Stiff ^5^, Leandro Castellano^5^, Sobia Idrees^6^, Matt D Johansen^6^, Alen Faiz^6^, Peter A Wark^7^, Philip M Hansbro^6,7^, Ian M. Adcock^8^, Gaetano Caramori^2^, Cristiana Stellato^1^.

^1^Department of Medicine, Surgery and Dentistry *'Scuola Medica Salernitana'*, University of Salerno, Salerno, Italy; ^2^ Respiratory Medicine Unit, Department of Biomedical Sciences, Dentistry and Morphological and Functional Imaging (BIOMORF), University of Messina, Messina, Italy; ^3^Department of Molecular Medicine, University of Padua, Padua, Italy, ^4^Department of Biology, University of Padua, Padua, Italy,  ^5^Department of Biochemistry and Biomedicine, School of Life Sciences, University of Sussex, UK; ^6^Centre for Inflammation, Centenary Institute and University of Technology Sydney, Faculty of Science, School of Life Sciences, Sydney NSW 2007, Australia, ^7^Immune Health, Hunter Medical Research Institute and The University of Newcastle, Newcastle, NSW 2208, Australia, ^8^National Heart and Lung Institute, Imperial College London and the NIHR Imperial Biomedical Research Centre, London, UK.

† These authors contributed equally to this work and share first authorship

1. Supplementary Tables and Figures

1.1 Supplementary Tables S1 – S9

1.2 Supplementary Figures S1- S6

1.3 Supplementary Figure Legends

**Table S1.** Study Reagents.

| Method | Reagent | Company |
| --- | --- | --- |
| Cell culture and treatments | DMEM | Euroclone |
|  | Ham’s F12 | Euroclone |
|  | FBS | Euroclone |
|  | L-glutamine | Lonza |
|  | Penicillin-streptomycin | Lonza |
|  | Fungizone | Euroclone |
|  | rHuIL-1β | GoldBio |
|  | TNFα | GoldBio |
|  | IFN-γ | GoldBio |
|  | FuGENE HD | Promega |
|  | AUF-1siRNA | Tema Ricerca |
|  | Scrambled control siRNA | Tema Ricerca |
|  | Actinomycin D | Sigma |
|  | MG-132 | Sigma |
|  | Trypsin/EDTA | Lonza |
|  | Trypan Blue | Euroclone |
| RIP-seq assay | RNase out | Thermo Fisher Scientific |
|  | Vanadyl-Ribonucleoside Complex | Thermo Fisher Scientific |
|  | Anti-AUF-1 Ab | Atlas Antibodies |
|  | IgG isotype | Thermo Fisher Scientific |
|  | Dynabeads | Thermo Fisher Scientific |
| Biotin pulldown assay | MAXIscript™ T7 Kits | Invitrogen |
|  | Biotin-11-cytidine-5’-triphosphate | Enzolife |
|  | Dynabeads | Invitrogen |
| Protein extraction and immunoblot | Tris/HCl pH 7.5 | Lonza |
|  | NaCl | PanReac Applichem |
|  | EDTA | Euroclone |
|  | EGTA | Sigma |
|  | NaF | Sigma |
|  | β-glycerolphosphate | Sigma |
|  | Na_3_VO_4_ | Cell Signaling Technology |
|  | PMSF | Sigma |
|  | Triton X-100 | Euroclone |
|  | Pierce ECL Western Blotting Substrate | Thermo Fisher Scientific |
|  | anti-AUF-1 | Atlas |
|  | anti- HuR | Santa Cruz |
|  | anti-TTP | Abcam |
|  | anti-p53 | Santa Cruz |
|  | anti-phospho-Rb | Cell Signaling Technology |
|  | anti-p21 | Cell Signaling Technology |
|  | anti-phospho-p53 | Cell Signaling Technology |
|  | anti- β-actin | Cell Signaling Technology |
|  | anti-β-tubulin | Cell Signaling Technology |
|  | anti-CD9 | Invitrogen |
|  | anti-CD63 | Invitrogen |
| RNA extraction, cDNA synthesis and qRT-PCR | TriFast | Euroclone |
|  | LunaScript® RT SuperMix Kits | New England Biolabs |
|  | FluoCycle II SYBR Master Mix | Euroclone |
| Cellular senescence assays | Etoposide | SelleckChem |
|  | Cell Meter™ Cellular Senescence Activity Assay Kits | AAT Bioquest |
| Analysis of SASP cytokines | LEGENDplexTM | Biolegend |
|  | Glutaraldehyde | Sigma |
|  | Epoxy resin | Sigma |
| Immunogold | PBS | Euroclone |
|  | BSA | Euroclone |
|  | IgG Gold II secondary anti-rabbit antibody coupled to gold particles | Sigma |
| Chemicals | HEPES | Euroclone |
|  | KCl | Carlo Erba |
|  | MgCl_2_ | Euroclone |
|  | NP40 | Sigma |
|  | DTT | GE Healthcare |
|  | Protease inhibitors | Cell Signaling Technology |

**Table S2.** Primers used for qPCR and biotin pulldown.

| Primer | | Sequence (5’-3’) |
| --- | --- | --- |
| DDX17 | **Forward** | GGATGTCTGCATGGAAAGTG |
|  | **Reverse** | TCAGATCATCACAGCGTCTC |
| EGR1 | **Forward** | CTTCAACCCTCAGGCGGACA |
|  | **Reverse** | GGAAAAGCGGCCAGTATAGGT |
| FOXP4 | **Forward** | CCAGGATGTTCGCCTATTTCCG |
|  | **Reverse** | TTTGGCGGTCTCCGCTTCTGAT |
| GAPDH | **Forward** | TGCACCACCAACTGCTTAGC |
|  | **Reverse** | GGCATGGACTGTGGTCATGAG |
| GLIS2 | **Forward** | GGAGAACCTGAAGATCCACAACC |
|  | **Reverse** | GCGTGTGCTTAAAGCGGTCACT |
| HDAC2 | **Forward** | GCTATTCCAGAAGATGCTGTTC |
|  | **Reverse** | GTTGCTGAGCTGTTCTGATTTG |
| IL-6 | **Forward** | GTAGCCGCCCCACACAGA |
|  | **Reverse** | CATGTCTCCTTTCTCAGGGCTG |
| MUC1 | **Forward** | CTTTCTTCCTGCTGCTGCTCCT |
|  | **Reverse** | AGCCGAAGTCTCCTTTTCTCCA |
| TGF-β1 | **Forward** | TACCTGAACCCGTGTTGCTCTC |
|  | **Reverse** | GTTGCTGAGGTATCGCCAGGAA |
| CyclinD1-3’UTR | **Forward (T7)** | GTGAATTGTAATACGACTCACTATAGGGGCTGTTTCACAATACCTCATGC |
|  | **Reverse** | TGTGAGCTGGCTTCATTGAG |
| DDX17-3’UTR A | **Forward (T7)** | GTGAATTGTAATACGACTCACTATAGGGTCACGTAAATGAAACCACTCAAG |
|  | **Reverse** | TTCACAGATGGTCCCCAGTT |
| DDX17-3’UTR B | **Forward (T7)** | GTGAATTGTAATACGACTCACTATAGGGCATCTCAGCATCTGGGTGGAA |
|  | **Reverse** | GAACCCCTCAGAAACCATCC |
| DDX17-3’UTR C | **Forward (T7)** | GTGAATTGTAATACGACTCACTATAGGGGCCTGAATGCTTGCTCATCTG |
|  | **Reverse** | ACAAGATGATGGTATCAAAAGGACA |
| EGR1-3’UTR | **Forward (T7)** | GTGAATTGTAATACGACTCACTATAGGGAAGGGAAAGGGGAAAGAAAGG |
|  | **Reverse** | TACACCACATATCCCATGGGCA |
| FoxP4-3’UTR A | **Forward (T7)** | GTGAATTGTAATACGACTCACTATAGGGAAGACAGGGACCTGGAGGAG |
|  | **Reverse** | GGCGACAAAGGAAAAAGCCA |
| FoxP4-3’UTR B | **Forward (T7)** | GTGAATTGTAATACGACTCACTATAGGGCCGGAAGCAAAAACCAAAACT |
|  | **Reverse** | TGTTTAAGCGTGGAAGGGACA |
| FoxP4-3’UTR C | **Forward (T7)** | GTGAATTGTAATACGACTCACTATAGGGAGTGGATGGAATGAGCAGCC |
|  | **Reverse** | CCCCTGCTCCCCAAATACAC |
| FoxP4-3’UTR D | **Forward (T7)** | GTGAATTGTAATACGACTCACTATAGGGTTCTGGACACTCCCTCCTTGA |
|  | **Reverse** | CCATTGTCCACAGATTACATGCAG |
| GLIS2-3’UTR A | **Forward (T7)** | GTGAATTGTAATACGACTCACTATAGGGTGGTGAACTGAGCCCATCCT |
|  | **Reverse** | GGAAGCAACGTGTCATGGGT |
| GLIS2-3’UTR B | **Forward (T7)** | GTGAATTGTAATACGACTCACTATAGGGAGTGCTGTGTTGGGAGCTTT |
|  | **Reverse** | TTCCTCTCCCATGGAGGAAT |
| HDAC2-3’UTR A | **Forward (T7)** | GTGAATTGTAATACGACTCACTATAGGGCCTGAATTTGACAGTCTCACCA |
|  | **Reverse** | GCACATCTTAGTAGCAGGAGT |
| HDAC2-3’UTR B | **Forward (T7)** | GTGAATTGTAATACGACTCACTATAGGGCACTCAGAGCTTACACTCAGAG |
|  | **Reverse** | CAGTTGTTGTCCCAATTCCAC |
| HDAC2-3’UTR C | **Forward (T7)** | GTGGAATTGGGACAACAACTG |
|  | **Reverse** | GTGAATTGTAATACGACTCACTATAGGGCTGAGGCACAGAGGTTAGTAAT |
| HDAC2-3’UTR D | **Forward (T7)** | GTGAATTGTAATACGACTCACTATAGGGGCCTCAGTTGCTTCATCATTT |
|  | **Reverse** | CACTGGCTGCAAAATGGGTC |
| HDAC2-3’UTR E | **Forward (T7)** | GTGAATTGTAATACGACTCACTATAGGGGACCCATTTTGCAGCCAGTG |
|  | **Reverse** | ACGCATCTCAAGGCCAGAAA |
| HDAC2-3’UTR F | **Forward (T7)** | GTGAATTGTAATACGACTCACTATAGGGTTTCTGGCCTTGAGATGCGT |
|  | **Reverse** | GCCAGTATCCTTGGGGGA |
| IL-6-3’UTR | **Forward (T7)** | GTGAATTGTAATACGACTCACTATAGGGGCTCTTCGGCAAATGTAGCA |
|  | **Reverse** | GTTAGCCATTTATTTGAGGTAAGCC |
| MUC1-3’UTR isoform 1 | **Forward (T7)** | GTGAATTGTAATACGACTCACTATAGGGCCAACTTGTAGGGGCACGTC |
|  | **Reverse** | GCAGTGGGAGACCACGTTTT |
| MUC1-3’UTR isoform 2 | **Forward (T7)** | GTGAATTGTAATACGACTCACTATAGGGCTATGTGCCCCCTAGCAGTA |
|  | **Reverse** | CAGTGGGAGACCACGTTTTATT |
| PDL1-3’UTR | **Forward (T7)** | GTGAATTGTAATACGACTCACTATAGGGTGTGCATGGAGAGGAAGACC |
|  | **Reverse** | TGGCTCCCAGAATTACCAAG |
| TGF-β1-3’UTR | **Forward (T7)** | GTGAATTGTAATACGACTCACTATAGGGTGTCCAACATGATCGTGCG |
|  | **Reverse** | TTGACCTCCCAGGATCAAGTGA |
| GC-rich Motif | **Forward (T7)** | GTGAATTGTAATACGACTCACTATAGGGCCTGTAATCTCAGCCTCCTGGGAGGCTGAGA |
|  | **Reverse** | TCTCAGCCTCCCAGGAGGCTGAGATTACAGGCCCTATAGTGAGTCGTATTACAATTCAC |

**Table S3.** Characteristics of peripheral lung tissue donors for generation of RNA sequencing database.

| Groups | n. | Age (years) | M/F | Ex/current smokers | Pack-years | Chronic bronchitis | FEV_1_ (% predicted) | FEV_1_/FVC% |
| --- | --- | --- | --- | --- | --- | --- | --- | --- |
| COPD patients | 7 | 71 ±4 | 6/1 | 5/2 | 57±32 | 1 | 74±20# | 60±9* |
| Smokers with normal lung function | 5 | 70 ±8 | 6/1 | 5/2 | 49±39 | 2 | 96±14 | 76±4 |

Abbreviations: COPD, chronic obstructive pulmonary disease; M, male; F, female; FEV_1_, forced expiratory volume in one second; FVC, forced vital capacity. For COPD and control smoker subjects, FEV_1_ % predicted and FEV_1_/FVC% are post-bronchodilator values. Data expressed as mean±SD. Statistical analysis: t test * p<0.0001, # p<0.05 COPD patients *vs* control smokers with normal lung function.

**Table S4. Full gene list of AUF-1-associated transcripts (n=494) identified by RIP-Seq analysis in unstimulated cells from the BEAS-2B line.** EF, Enriched Factor; FDR, False Discovery Rate. **Provided as separate file.**

**Table S5.**  RIP-Seq-derived AUF-1 targets (yellow columns, with EF and FDR) identified as DEG in the GSE5058 bronchiolar epithelium database (blue columns, FC ≥1.5) (Figure 8A depicts targets with FC ≥2).

| GSE5058 | | | | | | | | RIP-Seq | |
| --- | --- | --- | --- | --- | --- | --- | --- | --- | --- |
| Gene Symbol | **ID_REF_Gene Symbol** | **FC_S_vs_NS** | **FC_ COPDvs_NS** | **FC_ COPD_vs_S** | **FDR_S_vs_NS** | **FDR_ COPD_vs_NS** | **FDR_ COPD vs S** | **EF AUF1vs Input** | **FDR**  **AUF1_vsInput** |
| AAK1 | 205434_s_at_AAK1 | -1.17 | 2.03 | 2.37 | 0.469897 | 0.021086 | 0.032392 | 1.56 | 0.030407 |
| AASDH | 235435_at_AASDH | -1.00 | -3.38 | -3.38 | 0.440533 | 0.001294 | 0.039932 | 1.85 | 0.042012 |
| ABCD3 | 1554878_a_at_ABCD3 | 1.26 | -3.38 | -4.25 | 0.306527 | 0.003054 | 0.03357 | 1.69 | 0.013297 |
| ACADM | 202502_at_ACADM | -1.37 | -2.26 | -1.65 | 0.049184 | 0.002335 | 0.045288 | 1.75 | 0.010333 |
| ADAMTSL4 | 226071_at_ADAMTSL4 | 1.30 | 4.58 | 3.52 | 0.302344 | 0.006052 | 0.028377 | 1.91 | 0.015213 |
| ALDH7A1 | 213591_at_ALDH7A1 | -1.15 | 2.21 | 2.53 | 0.434129 | 0.002874 | 0.01685 | 1.64 | 0.014021 |
| ANAPC1 | 231973_s_at_ANAPC1 | -1.09 | -1.95 | -1.78 | 0.335864 | 0.001451 | 0.037583 | 1.69 | 0.011066 |
| ANKFY1 | 219868_s_at_ANKFY1 | 1.13 | -1.61 | -1.81 | 0.430412 | 0.020778 | 0.045572 | 1.85 | 0.00301 |
| ANKRD17 | 225852_at_ANKRD17 | 1.16 | -2.19 | -2.54 | 0.426806 | 0.006507 | 0.015434 | 1.55 | 0.049895 |
| ANKRD17 | 212211_at_ANKRD17 | -1.06 | -1.61 | -1.51 | 0.488767 | 0.000153 | 0.023213 | 1.55 | 0.049895 |
| APOOL | 213289_at_APOOL | 1.04 | -2.08 | -2.16 | 0.395676 | 0.00708 | 0.021562 | 1.66 | 0.030843 |
| AR | 226192_at_AR | 1.18 | 1.98 | 1.69 | 0.41147 | 0.006911 | 0.046728 | 1.86 | 0.009351 |
| ARF3 | 200734_s_at_ARF3 | -1.24 | 1.72 | 2.14 | 0.420739 | 0.030535 | 0.028699 | 1.53 | 0.024611 |
| ARHGEF7 | 235412_at_ARHGEF7 | 2.29 | -3.52 | -8.05 | 0.411111 | 0.069752 | 0.048745 | 1.55 | 0.045818 |
| ARHGEF7 | 242999_at_ARHGEF7 | 1.70 | -3.28 | -5.59 | 0.192607 | 0.107358 | 0.029696 | 1.55 | 0.045818 |
| ATG2B | 226684_at_ATG2B | 1.14 | -1.80 | -2.05 | 0.440552 | 0.011771 | 0.031026 | 1.75 | 0.003324 |
| ATP6V1A | 201971_s_at_ATP6V1A | 1.41 | -1.56 | -2.20 | 0.130562 | 0.040144 | 0.02084 | 1.62 | 0.042774 |
| BMPR2 | 238516_at_BMPR2 | 1.32 | -1.34 | -1.76 | 0.293807 | 0.04427 | 0.019536 | 1.52 | 0.044307 |
| BPTF | 209271_at_BPTF | 1.02 | -2.08 | -2.11 | 0.46733 | 0.029573 | 0.023045 | 1.51 | 0.03306 |
| BPTF | 207186_s_at_BPTF | 1.21 | -1.39 | -1.69 | 0.470249 | 0.094316 | 0.037789 | 1.51 | 0.03306 |
| BRD4 | 226052_at_BRD4 | -1.19 | 1.41 | 1.67 | 0.290003 | 0.005893 | 0.015718 | 1.96 | 0.001236 |
| CAND1 | 208839_s_at_CAND1 | 1.01 | -1.55 | -1.57 | 0.460691 | 0.0138 | 0.034811 | 1.54 | 0.049007 |
| CCDC82 | 223300_s_at_CCDC82 | 1.25 | -3.73 | -4.67 | 0.290011 | 0.011508 | 0.022287 | 1.64 | 0.043851 |
| CEBPZ | 203341_at_CEBPZ | 1.01 | -1.83 | -1.85 | 0.49135 | 0.03367 | 0.038277 | 1.87 | 0.006399 |
| CHD1 | 204258_at_CHD1 | 1.07 | -2.28 | -2.45 | 0.425096 | 0.013281 | 0.015718 | 1.72 | 0.022644 |
| CLASP1 | 240757_at_CLASP1 | 1.04 | -3.84 | -3.99 | 0.349083 | 0.122587 | 0.044096 | 1.59 | 0.023914 |
| CNOT1 | 1554052_at_CNOT1 | 1.15 | -2.07 | -2.39 | 0.422792 | 0.022938 | 0.046065 | 1.82 | 0.000347 |
| COL5A2 | 221729_at_COL5A2 | -1.09 | -2.17 | -1.99 | 0.41755 | 0.029534 | 0.03225 | 1.69 | 0.026243 |
| CPNE8 | 241706_at_CPNE8 | 1.32 | -1.40 | -1.84 | 0.32449 | 0.014355 | 0.027077 | 2.11 | 0.019199 |
| CREB3L1 | 213059_at_CREB3L1 | 1.01 | 2.20 | 2.18 | 0.341422 | 0.002041 | 0.02538 | 2.18 | 0.038525 |
| DDHD1 | 225971_at_DDHD1 | -1.12 | -1.96 | -1.74 | 0.287203 | 0.014641 | 0.044526 | 1.66 | 0.018784 |
| DDX17 | 230180_at_DDX17 | 1.17 | -13.61 | -15.92 | 0.383776 | 0.000387 | 0.047352 | 2.13 | 6.31E-06 |
| DDX3X | 212515_s_at_DDX3X | 1.23 | -2.09 | -2.58 | 0.403669 | 0.041865 | 0.037017 | 1.75 | 0.018266 |
| DHX36 | 223139_s_at_DHX36 | -1.07 | -5.31 | -4.96 | 0.407713 | 0.000859 | 0.011513 | 1.94 | 0.009784 |
| DHX36 | 223138_s_at_DHX36 | 1.08 | -2.58 | -2.79 | 0.483939 | 0.001143 | 0.021094 | 1.94 | 0.009784 |
| DHX36 | 223140_s_at_DHX36 | 1.00 | -1.72 | -1.72 | 0.48386 | 0.033389 | 0.033995 | 1.94 | 0.009784 |
| DHX40 | 222574_s_at_DHX40 | 1.18 | -1.84 | -2.17 | 0.294556 | 0.008248 | 0.025778 | 1.74 | 0.019459 |
| DOCK1 | 241708_at_DOCK1 | 1.42 | 3.14 | 2.21 | 0.248063 | 0.000427 | 0.021124 | 1.5 | 0.032698 |
| DOCK4 | 1558691_a_at_DOCK4 | 1.85 | -2.16 | -3.99 | 0.108956 | 0.108664 | 0.02247 | 1.77 | 0.017798 |
| EIF5B | 201024_x_at_EIF5B | 1.02 | -2.58 | -2.62 | 0.439861 | 0.013002 | 0.029949 | 1.91 | 0.013755 |
| FAM222B | 218464_s_at_FAM222B | 1.02 | 1.63 | 1.60 | 0.48386 | 0.020449 | 0.048974 | 1.72 | 0.033972 |
| FBXO11 | 222119_s_at_FBXO11 | -1.03 | -1.66 | -1.61 | 0.444602 | 0.018523 | 0.045116 | 1.73 | 0.017493 |
| FBXO30 | 242007_at_FBXO30 | 1.38 | -1.86 | -2.57 | 0.215344 | 0.057107 | 0.031788 | 1.85 | 0.004943 |
| FBXO38 | 219608_s_at_FBXO38 | 1.27 | -2.32 | -2.95 | 0.489286 | 0.011969 | 0.023259 | 1.68 | 0.046632 |
| FIGN | 238964_at_FIGN | 1.13 | 3.42 | 3.03 | 0.430784 | 0.002166 | 0.02247 | 1.83 | 0.005163 |
| G3BP2 | 208840_s_at_G3BP2 | 1.13 | -1.46 | -1.65 | 0.247302 | 0.065063 | 0.020908 | 1.78 | 0.002332 |
| GLCCI1 | 1560316_s_at_GLCCI1 | 1.14 | -2.26 | -2.57 | 0.417569 | 0.031549 | 0.044182 | 1.68 | 0.021442 |
| GLCCI1 | 227525_at_GLCCI1 | -1.12 | -1.79 | -1.60 | 0.442408 | 0.033697 | 0.046518 | 1.68 | 0.021442 |
| GLCCI1 | 225700_at_GLCCI1 | -1.05 | -1.57 | -1.50 | 0.476734 | 0.007117 | 0.044138 | 1.68 | 0.021442 |
| GLG1 | 207966_s_at_GLG1 | -1.01 | -1.97 | -1.94 | 0.366656 | 0.022271 | 0.027228 | 1.52 | 0.049989 |
| GLG1 | 214730_s_at_GLG1 | -1.11 | -1.84 | -1.65 | 0.293864 | 0.00855 | 0.031352 | 1.52 | 0.049989 |
| GLIS2 | 223378_at_GLIS2 | -1.46 | 1.22 | 1.78 | 0.089289 | 0.342734 | 0.020717 | 4.95 | 0.00745 |
| GPATCH8 | 212487_at_GPATCH8 | -1.52 | -6.80 | -4.49 | 0.138867 | 1.9E-05 | 0.038277 | 1.7 | 0.020406 |
| HIF1AN | 59999_at_HIF1AN | -1.05 | 1.74 | 1.82 | 0.387658 | 0.017402 | 0.019348 | 1.54 | 0.021424 |
| HNRNPM | 200072_s_at_HNRNPM | -1.01 | -1.58 | -1.56 | 0.368719 | 0.002015 | 0.030826 | 1.84 | 0.010043 |
| HSP90AA1 | 211968_s_at_HSP90AA1 | 1.12 | -1.76 | -1.98 | 0.38646 | 0.000162 | 0.015233 | 1.75 | 0.023752 |
| IBA57 | 231983_at_IBA57 | -1.33 | 2.46 | 3.28 | 0.249237 | 0.052393 | 0.015333 | 1.82 | 0.040616 |
| IGF1R | 203628_at_IGF1R | 1.24 | -2.34 | -2.91 | 0.307018 | 0.061091 | 0.026001 | 1.61 | 0.016693 |
| IGF1R | 243357_at_IGF1R | 1.35 | -1.42 | -1.92 | 0.171683 | 0.011102 | 0.009447 | 1.61 | 0.016693 |
| IL17RD | 229263_at_IL17RD | 1.51 | 3.64 | 2.40 | 0.482597 | 0.04187 | 0.049587 | 2.58 | 0.026678 |
| KDM6B | 41387_r_at_KDM6B | -1.11 | 1.36 | 1.52 | 0.40152 | 0.033248 | 0.047935 | 3.2 | 0.000139 |
| KDM6B | 41386_i_at_KDM6B | -1.39 | 1.13 | 1.58 | 0.106326 | 0.293015 | 0.02345 | 3.2 | 0.000139 |
| KIAA1109 | 216294_s_at_KIAA1109 | 1.33 | -2.61 | -3.48 | 0.20908 | 0.032229 | 0.038093 | 1.69 | 0.013803 |
| KLHL24 | 221986_s_at_KLHL24 | 1.23 | -2.50 | -3.07 | 0.415935 | 0.001575 | 0.046051 | 1.74 | 0.013386 |
| KLHL24 | 221985_at_KLHL24 | 1.23 | -1.31 | -1.61 | 0.280874 | 0.013276 | 0.024899 | 1.74 | 0.013386 |
| KLHL24 | 242088_at_KLHL24 | 1.29 | -1.21 | -1.56 | 0.050709 | 0.241907 | 0.022161 | 1.74 | 0.013386 |
| KLHL28 | 228328_at_KLHL28 | 1.08 | -2.28 | -2.47 | 0.494939 | 0.022483 | 0.032057 | 1.86 | 0.024761 |
| KMT2A | 212076_at_KMT2A | 1.51 | -1.38 | -2.08 | 0.366656 | 0.049826 | 0.023025 | 1.53 | 0.035849 |
| KMT2A | 212079_s_at_KMT2A | -1.00 | -1.64 | -1.64 | 0.370953 | 0.058962 | 0.027791 | 1.53 | 0.035849 |
| KPNA6 | 212103_at_KPNA6 | 1.47 | 2.53 | 1.72 | 0.049928 | 0.000452 | 0.036945 | 1.58 | 0.043402 |
| KPNB1 | 208974_x_at_KPNB1 | -1.15 | -2.28 | -1.98 | 0.497784 | 0.000462 | 0.026658 | 1.53 | 0.037058 |
| LIG4 | 206235_at_LIG4 | 1.34 | -3.38 | -4.53 | 0.368653 | 0.007339 | 0.021218 | 2.04 | 0.012687 |
| LPP | 214902_x_at_LPP | 1.15 | 3.72 | 3.24 | 0.394337 | 0.006533 | 0.030139 | 1.6 | 0.013544 |
| MACF1 | 208633_s_at_MACF1 | 1.23 | -1.98 | -2.43 | 0.271967 | 0.006087 | 0.024916 | 1.61 | 0.014615 |
| MACF1 | 215222_x_at_MACF1 | 1.13 | -2.05 | -2.33 | 0.306527 | 0.009828 | 0.040816 | 1.61 | 0.014615 |
| MAP1B | 214577_at_MAP1B | 1.70 | -1.78 | -3.02 | 0.150117 | 0.025256 | 0.037591 | 1.58 | 0.025353 |
| MAPK14 | 211561_x_at_MAPK14 | -1.05 | -1.85 | -1.76 | 0.491903 | 0.014739 | 0.023587 | 1.58 | 0.030753 |
| MAST4 | 210958_s_at_MAST4 | -1.13 | 1.73 | 1.95 | 0.48405 | 0.020565 | 0.029187 | 1.95 | 0.04438 |
| MAST4 | 40016_g_at_MAST4 | -1.11 | 1.81 | 2.00 | 0.36285 | 0.034997 | 0.026658 | 1.95 | 0.04438 |
| MAT2A | 200769_s_at_MAT2A | -1.04 | -2.85 | -2.75 | 0.469037 | 0.027406 | 0.045077 | 1.61 | 0.038683 |
| MED12 | 211342_x_at_MED12 | -1.46 | 1.14 | 1.67 | 0.13876 | 0.453603 | 0.028377 | 1.71 | 0.00733 |
| MED13 | 201987_at_MED13 | -1.00 | -1.80 | -1.80 | 0.455338 | 0.022127 | 0.025977 | 1.66 | 0.004775 |
| MIB1 | 224726_at_MIB1 | 1.17 | -2.50 | -2.93 | 0.382171 | 0.038152 | 0.049578 | 1.71 | 0.003195 |
| MIB1 | 224720_at_MIB1 | 1.03 | -2.13 | -2.19 | 0.392678 | 0.036959 | 0.031553 | 1.71 | 0.003195 |
| MIB1 | 224725_at_MIB1 | 1.21 | -1.32 | -1.60 | 0.25042 | 0.132352 | 0.048724 | 1.71 | 0.003195 |
| MICAL2 | 212473_s_at_MICAL2 | -1.33 | 1.23 | 1.64 | 0.197862 | 0.188408 | 0.021758 | 1.52 | 0.023687 |
| MICAL2 | 212472_at_MICAL2 | -1.04 | 1.70 | 1.76 | 0.451317 | 0.012835 | 0.02617 | 1.52 | 0.023687 |
| MLXIP | 202519_at_MLXIP | 1.05 | 1.85 | 1.77 | 0.389575 | 0.000469 | 0.015718 | 2.15 | 0.002861 |
| MNT | 204206_at_MNT | -1.08 | 2.02 | 2.19 | 0.417361 | 0.000195 | 0.012342 | 2.45 | 0.000654 |
| MPHOSPH8 | 225041_at_MPHOSPH8 | 1.02 | -2.32 | -2.37 | 0.497609 | 0.001795 | 0.021585 | 1.63 | 0.042752 |
| MPP5 | 219321_at_MPP5 | 1.25 | -1.61 | -2.02 | 0.272737 | 0.052324 | 0.044082 | 1.62 | 0.019258 |
| MYNN | 237510_at_MYNN | 1.18 | 1.88 | 1.60 | 0.330711 | 0.004663 | 0.031434 | 1.63 | 0.048625 |
| MYO6 | 203215_s_at_MYO6 | 1.29 | -4.49 | -5.79 | 0.475466 | 0.001185 | 0.034392 | 1.68 | 0.045818 |
| MYO6 | 210480_s_at_MYO6 | 1.07 | -2.34 | -2.52 | 0.467418 | 0.002177 | 0.042972 | 1.68 | 0.045818 |
| NATD1 | 226657_at_NATD1 | -1.17 | 1.29 | 1.51 | 0.21341 | 0.193051 | 0.033539 | 1.92 | 0.007202 |
| NAV2 | 222599_s_at_NAV2 | 1.76 | 4.36 | 2.48 | 0.03297 | 0.00012 | 0.035452 | 1.6 | 0.027014 |
| NEK9 | 214738_s_at_NEK9 | 1.37 | -4.27 | -5.83 | 0.462558 | 0.002099 | 0.039716 | 1.75 | 0.015949 |
| NEU3 | 235474_at_NEU3 | -1.05 | 1.55 | 1.62 | 0.44297 | 0.000808 | 0.02084 | 1.58 | 0.041545 |
| NFATC2IP | 217527_s_at_NFATC2IP | -1.11 | 1.57 | 1.75 | 0.249452 | 0.018538 | 0.025802 | 2.06 | 4.41E-05 |
| NFIX | 227400_at_NFIX | -1.59 | 1.40 | 2.24 | 0.212993 | 0.339383 | 0.039185 | 2.19 | 0.000846 |
| NHLRC2 | 219353_at_NHLRC2 | -1.15 | -2.05 | -1.78 | 0.377217 | 0.011489 | 0.034655 | 1.71 | 0.013803 |
| NIPBL | 207108_s_at_NIPBL | 1.26 | -3.22 | -4.06 | 0.306829 | 0.009912 | 0.01765 | 1.59 | 0.029242 |
| NIPBL | 242352_at_NIPBL | 1.09 | -2.76 | -3.00 | 0.354387 | 0.008901 | 0.030065 | 1.59 | 0.029242 |
| NIPBL | 213918_s_at_NIPBL | 1.18 | -1.90 | -2.24 | 0.411864 | 0.017114 | 0.042183 | 1.59 | 0.029242 |
| NPEPPS | 201454_s_at_NPEPPS | 1.08 | -1.81 | -1.96 | 0.448679 | 0.000846 | 0.023737 | 1.72 | 0.012968 |
| NUP214 | 202155_s_at_NUP214 | -1.07 | 1.65 | 1.77 | 0.495541 | 0.017702 | 0.046899 | 1.72 | 0.032662 |
| PAFAH1B1 | 211547_s_at_PAFAH1B1 | 1.11 | -2.43 | -2.69 | 0.349264 | 0.011298 | 0.03472 | 1.57 | 0.011198 |
| PAFAH1B1 | 200813_s_at_PAFAH1B1 | 1.06 | -1.97 | -2.08 | 0.390348 | 0.00272 | 0.021585 | 1.57 | 0.011198 |
| PALLD | 200906_s_at_PALLD | 1.12 | -1.74 | -1.95 | 0.330068 | 0.017215 | 0.027344 | 1.63 | 0.00675 |
| PALLD | 200907_s_at_PALLD | 1.23 | -1.31 | -1.61 | 0.192379 | 0.023643 | 0.022581 | 1.63 | 0.00675 |
| PAPSS1 | 209043_at_PAPSS1 | -1.28 | -2.05 | -1.61 | 0.022568 | 0.000345 | 0.014553 | 1.67 | 0.048926 |
| PBRM1 | 224152_s_at_PBRM1 | 1.52 | -4.58 | -6.94 | 0.258331 | 0.013385 | 0.021562 | 1.63 | 0.014761 |
| PDP1 | 218273_s_at_PDP1 | 1.23 | -2.45 | -3.01 | 0.316797 | 0.014357 | 0.03987 | 1.61 | 0.026678 |
| PDPR | 224902_at_PDPR | -1.14 | 1.54 | 1.75 | 0.299116 | 0.036624 | 0.021292 | 1.5 | 0.045776 |
| PEG10 | 212092_at_PEG10 | -1.45 | -5.32 | -3.66 | 0.192581 | 0.002426 | 0.036816 | 1.58 | 0.017043 |
| PHIP | 244811_at_PHIP | 1.17 | -3.10 | -3.64 | 0.447478 | 0.007906 | 0.025977 | 1.65 | 0.037967 |
| PHIP | 213074_at_PHIP | 1.05 | -1.77 | -1.85 | 0.447478 | 0.073817 | 0.040042 | 1.65 | 0.037967 |
| PIK3C2A | 1553694_a_at_PIK3C2A | 1.33 | -2.72 | -3.62 | 0.261245 | 0.009931 | 0.028677 | 1.81 | 0.005266 |
| PIK3C2A | 226094_at_PIK3C2A | 1.22 | -1.79 | -2.18 | 0.221601 | 0.069455 | 0.02617 | 1.81 | 0.005266 |
| PJA2 | 201133_s_at_PJA2 | 1.39 | -1.43 | -1.99 | 0.173598 | 0.047273 | 0.021292 | 1.57 | 0.04553 |
| PKM | 213700_s_at_PKM | -1.15 | 2.02 | 2.32 | 0.414636 | 0.01697 | 0.023045 | 1.6 | 0.028566 |
| PML | 211012_s_at_PML | -1.96 | 1.41 | 2.75 | 0.054697 | 0.193539 | 0.015959 | 1.64 | 0.023736 |
| POLR3A | 231763_at_POLR3A | -1.03 | 2.15 | 2.22 | 0.429949 | 0.003455 | 0.025678 | 1.74 | 0.017697 |
| PPP3CA | 202457_s_at_PPP3CA | 1.16 | -1.46 | -1.68 | 0.286445 | 0.050372 | 0.026952 | 1.52 | 0.029034 |
| PPP3CA | 202429_s_at_PPP3CA | 1.09 | -1.44 | -1.57 | 0.390447 | 0.081938 | 0.037011 | 1.52 | 0.029034 |
| PSMD12 | 202353_s_at_PSMD12 | -1.15 | -2.60 | -2.27 | 0.22525 | 0.009828 | 0.04135 | 1.55 | 0.035743 |
| PTPN14 | 244533_at_PTPN14 | 1.14 | 1.75 | 1.53 | 0.445141 | 0.029175 | 0.037843 | 1.72 | 0.001692 |
| PTPN14 | 242321_at_PTPN14 | 1.04 | 2.53 | 2.44 | 0.359104 | 0.003942 | 0.022577 | 1.72 | 0.001692 |
| PUM2 | 201493_s_at_PUM2 | -1.04 | -1.75 | -1.68 | 0.34902 | 0.004249 | 0.039947 | 1.51 | 0.031876 |
| PUM2 | 216221_s_at_PUM2 | -1.05 | -1.73 | -1.65 | 0.191929 | 0.002799 | 0.021477 | 1.51 | 0.031876 |
| QSER1 | 229982_at_QSER1 | 1.05 | -3.51 | -3.68 | 0.411361 | 0.000748 | 0.046065 | 1.6 | 0.032528 |
| RAB14 | 200927_s_at_RAB14 | 1.01 | -1.55 | -1.55 | 0.440977 | 0.036261 | 0.044546 | 2 | 0.000195 |
| RANBP2 | 201711_x_at_RANBP2 | 1.57 | -2.64 | -4.15 | 0.248063 | 0.001892 | 0.033242 | 1.64 | 0.019229 |
| RANBP2 | 201712_s_at_RANBP2 | 1.11 | -1.93 | -2.15 | 0.391851 | 0.022487 | 0.022129 | 1.64 | 0.019229 |
| RANBP2 | 201713_s_at_RANBP2 | 1.17 | -1.60 | -1.88 | 0.354566 | 0.073275 | 0.044033 | 1.64 | 0.019229 |
| RAP1GAP2 | 213280_at_RAP1GAP2 | 1.02 | 1.65 | 1.62 | 0.403616 | 0.048223 | 0.042426 | 1.67 | 0.028754 |
| RBM15 | 1555760_a_at_RBM15 | -1.09 | -1.71 | -1.57 | 0.406012 | 0.010336 | 0.029522 | 1.65 | 0.026375 |
| RECQL | 210568_s_at_RECQL | 1.57 | -1.03 | -1.62 | 0.015847 | 0.493413 | 0.028047 | 1.82 | 0.030015 |
| RFX7 | 222630_at_RFX7 | -1.09 | -1.68 | -1.54 | 0.456712 | 0.024857 | 0.043991 | 1.58 | 0.022644 |
| RIN3 | 60471_at_RIN3 | 1.12 | 2.09 | 1.87 | 0.185975 | 0.000402 | 0.044842 | 2.42 | 0.005946 |
| RPAP3 | 1557984_s_at_RPAP3 | 1.23 | -2.98 | -3.67 | 0.47592 | 0.009286 | 0.027228 | 1.82 | 0.020991 |
| RPAP3 | 218842_at_RPAP3 | -1.00 | -1.92 | -1.92 | 0.485727 | 0.033656 | 0.026425 | 1.82 | 0.020991 |
| SART3 | 209127_s_at_SART3 | 1.38 | -3.75 | -5.18 | 0.448871 | 0.003597 | 0.028107 | 1.89 | 0.004504 |
| SBNO1 | 229528_at_SBNO1 | 1.63 | 2.81 | 1.73 | 0.024532 | 0.000106 | 0.020341 | 1.63 | 0.013508 |
| SCAF11 | 209376_x_at_SCAF11 | 1.13 | -12.70 | -14.40 | 0.409218 | 3.86E-05 | 0.026344 | 1.64 | 0.031544 |
| SCAF11 | 213850_s_at_SCAF11 | 1.26 | -4.84 | -6.11 | 0.394189 | 0.0001 | 0.03215 | 1.64 | 0.031544 |
| SEC23IP | 216392_s_at_SEC23IP | 1.26 | -1.98 | -2.49 | 0.242411 | 0.002837 | 0.015747 | 1.78 | 0.037567 |
| SEC24D | 215641_at_SEC24D | 1.64 | 2.74 | 1.67 | 0.035803 | 0.000836 | 0.02617 | 1.83 | 0.01381 |
| SEMA3A | 206805_at_SEMA3A | 1.21 | -1.35 | -1.63 | 0.136496 | 0.13797 | 0.023561 | 1.63 | 0.026078 |
| SETX | 201965_s_at_SETX | 1.30 | -1.56 | -2.02 | 0.204946 | 0.027311 | 0.030127 | 1.64 | 0.018609 |
| SETX | 201964_at_SETX | 1.10 | -1.68 | -1.85 | 0.30036 | 0.007616 | 0.019536 | 1.64 | 0.018609 |
| SF3A2 | 209381_x_at_SF3A2 | -1.15 | 2.05 | 2.35 | 0.461391 | 0.01213 | 0.022581 | 2.07 | 0.004451 |
| SMARCC1 | 201072_s_at_SMARCC1 | 1.98 | -3.68 | -7.28 | 0.217396 | 0.010509 | 0.038469 | 1.53 | 0.043732 |
| SMARCC1 | 201073_s_at_SMARCC1 | 1.14 | -1.87 | -2.13 | 0.410596 | 0.024456 | 0.015434 | 1.53 | 0.043732 |
| SMC3 | 209257_s_at_SMC3 | 1.73 | -15.76 | -27.21 | 0.284797 | 0.003181 | 0.038615 | 1.98 | 0.006401 |
| SON | 201085_s_at_SON | -1.03 | -2.56 | -2.49 | 0.499061 | 0.003234 | 0.044315 | 1.98 | 0.000719 |
| SPATA13 | 1557470_at_SPATA13 | 1.21 | -6.54 | -7.93 | 0.47783 | 0.04039 | 0.041628 | 1.62 | 0.049615 |
| SRP54 | 203605_at_SRP54 | 1.19 | -1.28 | -1.53 | 0.237159 | 0.031881 | 0.017681 | 1.65 | 0.049409 |
| SRRM2 | 208610_s_at_SRRM2 | -1.20 | -4.28 | -3.57 | 0.29185 | 0.005391 | 0.019718 | 1.96 | 8.29E-05 |
| STK35 | 225649_s_at_STK35 | -1.13 | 1.36 | 1.54 | 0.397367 | 0.035871 | 0.022129 | 1.71 | 0.008742 |
| SYNCRIP | 209024_s_at_SYNCRIP | 1.68 | -2.16 | -3.64 | 0.139935 | 0.001091 | 0.019507 | 1.57 | 0.048277 |
| SYNPO | 202796_at_SYNPO | -1.11 | 1.55 | 1.71 | 0.340283 | 0.007842 | 0.018358 | 2.43 | 8.11E-07 |
| TAF2 | 1554721_a_at_TAF2 | 1.10 | -1.79 | -1.97 | 0.378109 | 0.026022 | 0.042491 | 1.59 | 0.047451 |
| TBL1X | 213400_s_at_TBL1X | -1.40 | 1.25 | 1.76 | 0.131101 | 0.073089 | 0.023916 | 1.65 | 0.026678 |
| TBL1X | 213401_s_at_TBL1X | 1.09 | 1.97 | 1.80 | 0.302503 | 0.004359 | 0.031888 | 1.65 | 0.026678 |
| TFCP2 | 207627_s_at_TFCP2 | 1.22 | -1.83 | -2.23 | 0.208759 | 0.002788 | 0.013395 | 1.55 | 0.046532 |
| TNKS2 | 222563_s_at_TNKS2 | 1.33 | -3.34 | -4.44 | 0.219443 | 0.015904 | 0.036618 | 1.53 | 0.028566 |
| TNKS2 | 222562_s_at_TNKS2 | 1.18 | -2.83 | -3.33 | 0.388121 | 0.000512 | 0.029335 | 1.53 | 0.028566 |
| TNRC6B | 229036_at_TNRC6B | 1.26 | -2.93 | -3.70 | 0.3011 | 0.001458 | 0.021562 | 1.55 | 0.026788 |
| TNRC6B | 228998_at_TNRC6B | -1.05 | -2.53 | -2.40 | 0.475551 | 0.012412 | 0.045977 | 1.55 | 0.026788 |
| TNRC6B | 213254_at_TNRC6B | 1.22 | -1.29 | -1.57 | 0.205661 | 0.094073 | 0.032707 | 1.55 | 0.026788 |
| TOP1 | 208900_s_at_TOP1 | 1.24 | -3.31 | -4.11 | 0.409758 | 0.001103 | 0.038938 | 1.96 | 0.007066 |
| TOPORS | 204071_s_at_TOPORS | -1.09 | -2.57 | -2.35 | 0.436596 | 0.018467 | 0.026884 | 1.78 | 0.014124 |
| TRPS1 | 224218_s_at_TRPS1 | 1.37 | -1.47 | -2.00 | 0.14553 | 0.059401 | 0.015233 | 1.77 | 0.02826 |
| TSC22D2 | 240557_at_TSC22D2 | 1.37 | -1.75 | -2.39 | 0.34754 | 0.181367 | 0.048724 | 1.61 | 0.020438 |
| TSC22D4 | 1554501_at_TSC22D4 | -1.18 | 1.69 | 2.00 | 0.328236 | 0.053432 | 0.027344 | 1.79 | 0.024377 |
| TUG1 | 212337_at_TUG1 | 1.06 | -2.26 | -2.39 | 0.478324 | 0.00011 | 0.022844 | 1.68 | 0.010064 |
| TULP3 | 221964_at_TULP3 | -1.27 | 1.18 | 1.50 | 0.222392 | 0.100996 | 0.026097 | 1.65 | 0.036366 |
| UBR5 | 208884_s_at_UBR5 | 1.09 | -1.49 | -1.63 | 0.472783 | 0.002485 | 0.02538 | 2.09 | 1.57E-05 |
| USP34 | 242647_at_USP34 | 1.73 | -6.80 | -11.77 | 0.288597 | 0.039172 | 0.033316 | 1.61 | 0.009784 |
| USP34 | 215013_s_at_USP34 | 1.16 | -3.11 | -3.59 | 0.468635 | 0.03508 | 0.049295 | 1.61 | 0.009784 |
| USP34 | 212065_s_at_USP34 | 1.26 | -2.83 | -3.57 | 0.320041 | 0.00106 | 0.039308 | 1.61 | 0.009784 |
| USP34 | 212066_s_at_USP34 | 1.09 | -1.41 | -1.53 | 0.359183 | 0.051259 | 0.048112 | 1.61 | 0.009784 |
| USP9X | 201100_s_at_USP9X | 1.24 | 1.87 | 1.51 | 0.077146 | 2.04E-05 | 0.012372 | 1.9 | 0.000813 |
| WDFY3 | 212606_at_WDFY3 | 1.17 | -1.52 | -1.77 | 0.429509 | 0.02524 | 0.046227 | 1.78 | 0.001371 |
| WNK1 | 211992_at_WNK1 | 1.21 | -1.43 | -1.73 | 0.264998 | 0.048711 | 0.030785 | 1.68 | 0.003319 |
| YLPM1 | 214659_x_at_YLPM1 | 1.07 | -1.53 | -1.64 | 0.487691 | 0.040143 | 0.040036 | 2.03 | 0.0003 |
| YTHDC2 | 1568680_s_at_YTHDC2 | 1.49 | -2.24 | -3.34 | 0.20568 | 0.001661 | 0.045361 | 1.77 | 0.045855 |
| ZBTB10 | 219312_s_at_ZBTB10 | -1.18 | -2.69 | -2.28 | 0.401266 | 0.011927 | 0.027027 | 1.71 | 0.016877 |
| ZFP36L2 | 201369_s_at_ZFP36L2 | -1.09 | 2.08 | 2.27 | 0.313641 | 0.037188 | 0.027057 | 1.65 | 0.042908 |
| ZMIZ2 | 54970_at_ZMIZ2 | -1.23 | 1.39 | 1.71 | 0.163166 | 0.019464 | 0.011579 | 2 | 0.003344 |
| ZNF148 | 203319_s_at_ZNF148 | 1.24 | -2.69 | -3.34 | 0.35783 | 0.012998 | 0.027824 | 1.67 | 0.006623 |
| ZNF462 | 244007_at_ZNF462 | -1.26 | 1.27 | 1.59 | 0.276726 | 0.058012 | 0.024916 | 1.66 | 0.006012 |
| ZNF609 | 212620_at_ZNF609 | -1.10 | -1.92 | -1.74 | 0.47134 | 0.049823 | 0.045391 | 1.53 | 0.025731 |

**Table S6.**  RIP-Seq-derived AUF-1 targets identified as DEG in RNAseq database of lung biopsies from COPD vs matched smoker controls (see also Fig. 8A).

| hgnc_symbol | ensembl_gene_id | Entrez gene_id | baseMean | log_2_FoldChange | lfcSE | stat | pvalue | padj |
| --- | --- | --- | --- | --- | --- | --- | --- | --- |
| PTK2B | ENSG00000120899 | 2185 | 396.0882 | 1.52 | 0.261798 | 5.797319 | 6.74E-09 | 7.62E-06 |
| SF3A2 | ENSG00000104897 | 8175 | 73.79135 | 1.28 | 0.262028 | 4.9039 | 9.40E-07 | 0.000227 |
| IRS2 | ENSG00000185950 | 8660 | 452.0256 | 1.08 | 0.214528 | 5.05681 | 4.26E-07 | 0.000136 |
| PER1 | ENSG00000179094 | 5187 | 408.6825 | 0.91 | 0.295455 | 3.09459 | 0.001971 | 0.034843 |
| GRINA | ENSG00000178719 | 2907 | 305.7394 | 0.86 | 0.275986 | 3.114607 | 0.001842 | 0.033724 |
| CRYBG1 | ENSG00000112297 | 202 | 149.2555 | 0.75 | 0.223963 | 3.367845 | 0.000758 | 0.018993 |
| CPSF7 | ENSG00000149532 | 79869 | 144.137 | 0.75 | 0.184255 | 4.07881 | 4.53E-05 | 0.002941 |
| CORO1C | ENSG00000110880 | 23603 | 182.7091 | 0.73 | 0.252345 | 2.91104 | 0.003602 | 0.048691 |
| SELENON | ENSG00000162430 | 57190 | 104.0103 | 0.73 | 0.231076 | 3.162768 | 0.001563 | 0.030312 |
| TRIM13 | ENSG00000204977 | 10206 | 100.0442 | 0.60 | 0.197568 | 3.046343 | 0.002316 | 0.038002 |
| SF1 | ENSG00000168066 | 7536 | 356.7719 | 0.50 | 0.16285 | 3.083675 | 0.002045 | 0.03569 |
| PBRM1 | ENSG00000163939 | 55193 | 207.7532 | -0.45 | 0.146572 | -3.04898 | 0.002296 | 0.037977 |
| SON | ENSG00000159140 | 6651 | 767.3564 | -0.57 | 0.155555 | -3.65121 | 0.000261 | 0.009887 |
| KPNB1 | ENSG00000108424 | 3837 | 380.6281 | -0.58 | 0.190597 | -3.0594 | 0.002218 | 0.037449 |
| BMPR2 | ENSG00000204217 | 659 | 354.92 | -0.62 | 0.207531 | -2.98079 | 0.002875 | 0.042681 |
| MYO6 | ENSG00000196586 | 4646 | 240.1765 | -0.66 | 0.1734 | -3.78205 | 0.000156 | 0.00681 |
| TRIP12 | ENSG00000153827 | 9320 | 247.407 | -0.66 | 0.217495 | -3.0333 | 0.002419 | 0.039 |
| DHX36 | ENSG00000174953 | 170506 | 160.1471 | -0.68 | 0.233418 | -2.92636 | 0.003429 | 0.047147 |
| SMC3 | ENSG00000108055 | 9126 | 135.904 | -0.70 | 0.215952 | -3.25658 | 0.001128 | 0.024646 |
| SMC1A | ENSG00000072501 | 8243 | 218.3076 | -0.71 | 0.168738 | -4.22021 | 2.44E-05 | 0.001903 |
| FBXO11 | ENSG00000138081 | 80204 | 90.73085 | -0.78 | 0.215722 | -3.63061 | 0.000283 | 0.010434 |
| ASXL2 | ENSG00000143970 | 55252 | 65.1955 | -0.81 | 0.225346 | -3.58398 | 0.000338 | 0.011887 |
| RANBP2 | ENSG00000153201 | 5903 | 120.36 | -0.82 | 0.22954 | -3.57238 | 0.000354 | 0.012144 |
| EIF4G3 | ENSG00000075151 | 8672 | 115.454 | -0.82 | 0.251236 | -3.26873 | 0.00108 | 0.024059 |
| CEBPZ | ENSG00000115816 | 10153 | 97.73255 | -0.83 | 0.213576 | -3.86863 | 0.000109 | 0.005592 |
| UBR3 | ENSG00000144357 | 130507 | 45.26861 | -0.84 | 0.272804 | -3.09168 | 0.00199 | 0.034957 |
| SETX | ENSG00000107290 | 23064 | 265.1972 | -0.86 | 0.166474 | -5.13655 | 2.80E-07 | 0.000102 |
| CHD1 | ENSG00000153922 | 1105 | 212.3894 | -0.86 | 0.290132 | -2.96632 | 0.003014 | 0.04378 |
| BPTF | ENSG00000171634 | 2186 | 531.0232 | -0.86 | 0.206082 | -4.17669 | 2.96E-05 | 0.002206 |
| PSMD12 | ENSG00000197170 | 5718 | 124.4738 | -0.86 | 0.2513 | -3.43894 | 0.000584 | 0.016298 |
| LPP | ENSG00000145012 | 4026 | 412.0042 | -0.87 | 0.204457 | -4.24004 | 2.23E-05 | 0.0018 |
| KIAA1109 | ENSG00000138688 | 84162 | 240.6623 | -0.89 | 0.24761 | -3.57435 | 0.000351 | 0.012087 |
| DDHD1 | ENSG00000100523 | 80821 | 88.86231 | -0.90 | 0.250807 | -3.57719 | 0.000347 | 0.01206 |
| MACF1 | ENSG00000127603 | 23499 | 2201.619 | -0.93 | 0.236038 | -3.92415 | 8.70E-05 | 0.004613 |
| DNMT1 | ENSG00000130816 | 1786 | 139.8132 | -0.93 | 0.184844 | -5.02291 | 5.09E-07 | 0.000154 |
| FRMD4B | ENSG00000114541 | 23150 | 174.164 | -0.94 | 0.232992 | -4.0224 | 5.76E-05 | 0.003506 |
| ICE1 | ENSG00000164151 | 23379 | 88.46696 | -0.94 | 0.211827 | -4.4537 | 8.44E-06 | 0.000955 |
| PDS5B | ENSG00000083642 | 23047 | 106.4105 | -0.95 | 0.192756 | -4.91084 | 9.07E-07 | 0.000224 |
| MTR | ENSG00000116984 | 4548 | 117.4341 | -0.99 | 0.203255 | -4.87876 | 1.07E-06 | 0.000253 |
| TET3 | ENSG00000187605 | 200424 | 22.10637 | -1.00 | 0.320757 | -3.1131 | 0.001851 | 0.033845 |
| DMXL1 | ENSG00000172869 | 1657 | 68.78459 | -1.02 | 0.291393 | -3.4864 | 0.00049 | 0.014716 |
| NEU3 | ENSG00000162139 | 10825 | 28.47665 | -1.03 | 0.339304 | -3.04736 | 0.002309 | 0.037987 |
| TRIM5 | ENSG00000132256 | 85363 | 35.78818 | -1.04 | 0.352397 | -2.94466 | 0.003233 | 0.045375 |
| ERC1 | ENSG00000082805 | 23085 | 124.4265 | -1.04 | 0.193759 | -5.36804 | 7.96E-08 | 4.58E-05 |
| LEMD3 | ENSG00000174106 | 23592 | 50.46957 | -1.07 | 0.260014 | -4.11646 | 3.85E-05 | 0.002597 |
| MKI67 | ENSG00000148773 | 4288 | 21.00531 | -1.14 | 0.387295 | -2.95523 | 0.003124 | 0.04447 |
| DNAJC13 | ENSG00000138246 | 23317 | 60.04671 | -1.19 | 0.224584 | -5.31574 | 1.06E-07 | 5.83E-05 |
| CRYBG3 | ENSG00000080200 | 131544 | 81.82177 | -1.32 | 0.225379 | -5.87238 | 4.30E-09 | 7.42E-06 |
| EGR1 | ENSG00000120738 | 1958 | 2022.615 | -1.34 | 0.332313 | -4.02393 | 5.72E-05 | 0.003506 |
| DOCK7 | ENSG00000116641 | 85440 | 17.04528 | -1.41 | 0.397187 | -3.54986 | 0.000385 | 0.012546 |
| SAMD9L | ENSG00000177409 | 219285 | 98.68918 | -1.42 | 0.401584 | -3.53722 | 0.000404 | 0.012961 |
| DOCK1 | ENSG00000150760 | 1793 | 77.0781 | -1.46 | 0.260014 | -5.60119 | 2.13E-08 | 1.71E-05 |

**Table S7.** Complete list of canonical pathways of all RIP-Seq-derived AUF-1 targets (n=494, Table S4), obtained from Ingenuity Pathway Analysis (IPA).

| Ingenuity Canonical Pathways | *p*-value | Ratio | Molecules |
| --- | --- | --- | --- |
| TR/RXR Activation | 6,0256E-05 | 0,1 | RAB3B,PIK3C2A,NCOA6,IRS2,THRA,BCL3,NCOR1,NCOR2,PIK3R4,DIO2 |
| Paxillin Signaling | 7,76247E-05 | 8,87E-02 | DOCK1,PXN,PAK4,MAPK14,PIK3C2A,PTK2B,ARHGEF7,PAK2,IRS2,PIK3R4,GIT2 |
| RANK Signaling in Osteoclasts | 8,31764E-05 | 9,62E-02 | TRAF6,MAP3K9,MAPK14,PIK3C2A,PTK2B,MAP3K1,IRS2,PIK3R4,XIAP,PPP3CA |
| Glucocorticoid Receptor Signaling | 0,000234423 | 5,43E-02 | PBRM1,PIK3C2A,SMAD3,MAP3K1,ARID2,PIK3R4,TRAF6,TAF1,AR,MAPK14,TGFB1,HSP90AA1,IRS2,NCOR1,NCOR2,SMARCC1,NRIP1,TAF2,PPP3CA |
| PPARα/RXRα Activation | 0,000245471 | 6,88E-02 | CAND1,TRAF6,MAPK14,TGFB1,SMAD3,HSP90AA1,NCOA6,BMPR2,NR2C2,NCOR1,BCL3,NCOR2,MED12 |
| FAK Signaling | 0,000630957 | 8,18E-02 | DOCK1,PXN,PAK4,PIK3C2A,ARHGEF7,PAK2,IRS2,PIK3R4,GIT2 |
| Folate Transformations I | 0,000758578 | 3,33E-01 | MTHFR,MTR,MTHFD1 |
| Germ Cell-Sertoli Cell Junction Signaling | 0,000812831 | 6,38E-02 | MAP3K9,PXN,PAK4,EPN1,MAPK14,PIK3C2A,TGFB1,PAK2,MAP3K1,CTNNA1,IRS2,PIK3R4 |
| RAR Activation | 0,000891251 | 6,32E-02 | PBRM1,MAPK14,TGFB1,SMAD3,MAP3K1,SORBS3,ARID2,NCOR1,NCOR2,SMARCC1,NRIP1,PML |
| Inosine-5'-phosphate Biosynthesis II | 0,001380384 | 6,67E-01 | PAICS,ATIC |
| Protein Ubiquitination Pathway | 0,002344229 | 5,17E-02 | USP14,MED20,USP9X,BIRC6,DNAJC13,ANAPC1,XIAP,TRAF6,USP13,PSMD12,HSP90AA1,USP40,NEDD4L,USP34 |
| Renin-Angiotensin Signaling | 0,002398833 | 6,77E-02 | PAK4,MAPK14,PIK3C2A,PTK2B,ITPR2,PAK2,MAP3K1,IRS2,PIK3R4 |
| Mitotic Roles of Polo-Like Kinase | 0,002951209 | 9,09E-02 | SMC3,TGFB1,WEE1,HSP90AA1,ANAPC1,SMC1A |
| PAK Signaling | 0,002951209 | 7,14E-02 | PXN,PAK4,PTK2B,PIK3C2A,ARHGEF7,PAK2,IRS2,PIK3R4 |
| p53 Signaling | 0,003090295 | 7,08E-02 | MAPK14,PIK3C2A,PLAGL1,CCNK,IRS2,PIK3R4,PML,TP53BP2 |
| EGF Signaling | 0,003981072 | 8,57E-02 | MAPK14,PIK3C2A,ITPR2,MAP3K1,IRS2,PIK3R4 |
| 2-ketoglutarate Dehydrogenase Complex | 0,004466836 | 0,4 | DLST,DLD |
| Lysine Degradation II | 0,004466836 | 0,4 | AASDH,ALDH7A1 |
| Lysine Degradation V | 0,004466836 | 0,4 | AASDH,ALDH7A1 |
| TGF-β Signaling | 0,004677351 | 7,29E-02 | TRAF6,RUNX3,MAPK14,RNF111,TGFB1,SMAD3,BMPR2 |
| RAN Signaling | 0,005370318 | 1,76E-01 | KPNB1,RANBP2,KPNA6 |
| HGF Signaling | 0,005495409 | 6,45E-02 | DOCK1,MAP3K9,PXN,PIK3C2A,CRKL,MAP3K1,IRS2,PIK3R4 |
| Clathrin-mediated Endocytosis Signaling | 0,005623413 | 5,29E-02 | MYO6,EPN1,PIK3C2A,USP9X,CLTC,AAK1,IRS2,PIK3R4,AP1G1,MYO1E,PPP3CA |
| Rac Signaling | 0,006309573 | 6,3E-02 | PAK4,PTK2B,PIK3C2A,MAP3K1,PAK2,PIKFYVE,IRS2,PIK3R4 |
| CXCR4 Signaling | 0,006456542 | 5,46E-02 | DOCK1,PXN,PAK4,PIK3C2A,ITPR2,GNA12,EGR1,PAK2,IRS2,PIK3R4 |
| Estrogen Receptor Signaling | 0,009772372 | 5,84E-02 | MED13,TAF1,MED20,NCOR1,NCOR2,NRIP1,MED12,TAF2 |
| Role of NFAT in Cardiac Hypertrophy | 0,011220185 | 4,8E-02 | MAPK14,HDAC2,PIK3C2A,CAMK1D,ITPR2,TGFB1,MAP3K1,IGF1R,IRS2,PIK3R4,PPP3CA |
| Integrin Signaling | 0,011220185 | 4,8E-02 | DOCK1,PXN,PAK4,PIK3C2A,ARF3,ARHGEF7,CRKL,PAK2,PIKFYVE,IRS2,PIK3R4 |
| Synaptogenesis Signaling Pathway | 0,012022644 | 4,28E-02 | MARCKS,STXBP1,MAPK14,PIK3C2A,STX1B,CRKL,ARHGEF7,MAP1B,NECTIN1,IRS2,PAFAH1B1,PIK3R4,SYT16,GRINA |
| SAPK/JNK Signaling | 0,012302688 | 6,09E-02 | MAP3K9,PIK3C2A,CRKL,GNA12,MAP3K1,IRS2,PIK3R4 |
| GNRH Signaling | 0,012882496 | 5,2E-02 | MAP3K9,PXN,PAK4,MAPK14,PTK2B,ITPR2,EGR1,PAK2,MAP3K1 |
| Chronic Myeloid Leukemia Signaling | 0,012882496 | 6,03E-02 | PIK3C2A,HDAC2,TGFB1,SMAD3,CRKL,IRS2,PIK3R4 |
| Actin Cytoskeleton Signaling | 0,012882496 | 4,7E-02 | DOCK1,PXN,PAK4,FN1,PIK3C2A,ARHGEF7,CRKL,GNA12,PAK2,IRS2,PIK3R4 |
| Mouse Embryonic Stem Cell Pluripotency | 0,013489629 | 5,98E-02 | MAPK14,PIK3C2A,BMPR2,IRS2,TCF7L1,PIK3R4,XIAP |
| Role of Osteoblasts, Osteoclasts and Chondrocytes in Rheumatoid Arthritis | 0,013803843 | 4,66E-02 | TRAF6,MAPK14,PIK3C2A,PTK2B,TGFB1,BMPR2,IRS2,TCF7L1,PIK3R4,XIAP,PPP3CA |
| TCA Cycle II (Eukaryotic) | 0,014454398 | 1,25E-01 | SUCLA2,DLST,DLD |
| FGF Signaling | 0,014454398 | 6,52E-02 | MAPK14,PIK3C2A,CRKL,MAP3K1,IRS2,PIK3R4 |
| Leucine Degradation I | 0,015135612 | 2,22E-01 | ACADM,MCCC2 |
| Renal Cell Carcinoma Signaling | 0,015135612 | 6,45E-02 | PAK4,PIK3C2A,TGFB1,PAK2,IRS2,PIK3R4 |
| Reelin Signaling in Neurons | 0,016218101 | 6,38E-02 | MAP3K9,PIK3C2A,CRKL,IRS2,PAFAH1B1,PIK3R4 |
| Osteoarthritis Pathway | 0,016982437 | 4,72E-02 | SIK3,FN1,GLIS2,GLI3,TGFB1,SMAD3,CTNNA1,BMPR2,RBPJ,TCF7L1 |
| IL-9 Signaling | 0,018197009 | 8,51E-02 | PIK3C2A,IRS2,BCL3,PIK3R4 |
| Hereditary Breast Cancer Signaling | 0,019498446 | 5,16E-02 | PBRM1,HDAC2,PIK3C2A,WEE1,IRS2,ARID2,SMARCC1,PIK3R4 |
| PDGF Signaling | 0,021877616 | 5,94E-02 | PIK3C2A,ABL2,CRKL,MAP3K1,IRS2,PIK3R4 |
| Purine Nucleotides De Novo Biosynthesis II | 0,022387211 | 1,82E-01 | PAICS,ATIC |
| TNFR1 Signaling | 0,022387211 | 0,08 | PAK4,PAK2,MAP3K1,XIAP |
| Acute Myeloid Leukemia Signaling | 0,023988329 | 5,83E-02 | RUNX1,PIK3C2A,IRS2,TCF7L1,PML,PIK3R4 |
| Signaling by Rho Family GTPases | 0,025118864 | 4,25E-02 | MAP3K9,PAK4,PIK3C2A,PTK2B,ARHGEF7,GNA12,PAK2,PIKFYVE,CDC42EP1,IRS2,PIK3R4 |
| Assembly of RNA Polymerase I Complex | 0,02630268 | 1,67E-01 | POLR1A,POLR1B |
| Role of Tissue Factor in Cancer | 0,026915348 | 5,19E-02 | MAPK14,PTK2B,PIK3C2A,GNA12,EGR1,IRS2,PIK3R4 |
| B Cell Receptor Signaling | 0,027542287 | 4,57E-02 | MAP3K9,MAPK14,PIK3C2A,PTK2B,EGR1,MAP3K1,IRS2,PIK3R4,PPP3CA |
| Molecular Mechanisms of Cancer | 0,028840315 | 3,73E-02 | PAK4,PIK3C2A,GNA12,ARHGEF7,SMAD3,CTNNA1,BMPR2,PIK3R4,XIAP,MAPK14,NF1,TGFB1,PAK2,RBPJ,IRS2 |
| IL-17A Signaling in Airway Cells | 0,029512092 | 6,25E-02 | TRAF6,MAPK14,PIK3C2A,IRS2,PIK3R4 |
| CD40 Signaling | 0,030902954 | 6,17E-02 | TRAF6,MAPK14,PIK3C2A,IRS2,PIK3R4 |
| ErbB Signaling | 0,030902954 | 5,5E-02 | PAK4,MAPK14,PIK3C2A,PAK2,IRS2,PIK3R4 |
| CCR3 Signaling in Eosinophils | 0,032359366 | 0,05 | PAK4,MAPK14,PIK3C2A,ITPR2,PAK2,IRS2,PIK3R4 |
| Circadian Rhythm Signaling | 0,033884416 | 9,09E-02 | PER1,BHLHE40,GRINA |
| GM-CSF Signaling | 0,035481339 | 5,95E-02 | RUNX1,PIK3C2A,IRS2,PIK3R4,PPP3CA |
| Aryl Hydrocarbon Receptor Signaling | 0,035481339 | 4,9E-02 | NCOA7,NFIX,TGFB1,HSP90AA1,NCOR2,NRIP1,ALDH7A1 |
| DNA Double-Strand Break Repair by Non-Homologous End Joining | 0,035481339 | 1,43E-01 | LIG4,LIG3 |
| Amyotrophic Lateral Sclerosis Signaling | 0,035481339 | 5,31E-02 | PIK3C2A,IRS2,PIK3R4,XIAP,PPP3CA,GRINA |
| DNA Methylation and Transcriptional Repression Signaling | 0,036307805 | 8,82E-02 | MECP2,HDAC2,DNMT1 |
| ERK/MAPK Signaling | 0,037153523 | 4,33E-02 | DOCK1,PXN,PAK4,PIK3C2A,PTK2B,CRKL,PAK2,IRS2,PIK3R4 |
| Nitric Oxide Signaling in the Cardiovascular System | 0,03801894 | 5,22E-02 | PIK3C2A,ITPR2,HSP90AA1,IRS2,PIK3R4,PDE1C |
| Gα12/13 Signaling | 0,038904514 | 4,79E-02 | PXN,PTK2B,PIK3C2A,GNA12,MAP3K1,IRS2,PIK3R4 |
| Human Embryonic Stem Cell Pluripotency | 0,040738028 | 4,76E-02 | PIK3C2A,TGFB1,SMAD3,BMPR2,IRS2,TCF7L1,PIK3R4 |
| IL-23 Signaling Pathway | 0,040738028 | 6,67E-02 | RUNX1,PIK3C2A,IRS2,PIK3R4 |
| Neuropathic Pain Signaling In Dorsal Horn Neurons | 0,040738028 | 5,13E-02 | CAMK1D,PIK3C2A,ITPR2,IRS2,PIK3R4,GRINA |
| Choline Degradation I | 0,042657952 | 0,5 | ALDH7A1 |
| Sulfate Activation for Sulfonation | 0,042657952 | 0,5 | PAPSS1 |
| Angiopoietin Signaling | 0,043651583 | 5,62E-02 | PAK4,PIK3C2A,PAK2,IRS2,PIK3R4 |
| Estrogen-Dependent Breast Cancer Signaling | 0,044668359 | 5,56E-02 | PIK3C2A,IGF1R,IRS2,PIK3R4,HSD17B4 |
| Superpathway of Methionine Degradation | 0,044668359 | 8,11E-02 | DLD,MAT2A,MTR |
| Cardiac Hypertrophy Signaling | 0,048977882 | 3,94E-02 | MAP3K9,MAPK14,PIK3C2A,TGFB1,GNA12,MAP3K1,IGF1R,IRS2,PIK3R4,PPP3CA |

**Table S8.** Canonical pathways of RIP-Seq-derived AUF-1 targets identified as DEG in the GSE5058 database (Table S5) from Ingenuity Pathway Analysis (IPA).

| Ingenuity Canonical Pathways | -log(p-value) | Ratio | Molecules |
| --- | --- | --- | --- |
| RAN Signaling | 3,82E00 | 1,76E-01 | KPNA6,KPNB1,RANBP2 |
| Ribonucleotide Reductase Signaling Pathway | 3,16E00 | 3,53E-02 | CREB3L1,MAPK14,PBRM1,PIK3C2A,SMARCC1,TNKS2 |
| MicroRNA Biogenesis Signaling Pathway | 2,94E00 | 3,21E-02 | DDX17,HSP90AA1,MAPK14,NUP214,PIK3C2A,SF3A2 |
| Prostate Cancer Signaling | 2,25E00 | 3,51E-02 | AR,CREB3L1,HSP90AA1,PIK3C2A |
| PPARα/RXRα Activation | 2,12E00 | 2,56E-02 | BMPR2,CAND1,HSP90AA1,MAPK14,MED12 |
| Mitotic Roles of Polo-Like Kinase | 2,07E00 | 4,48E-02 | ANAPC1,HSP90AA1,SMC3 |
| Macrophage Alternative Activation Signaling Pathway | 2,06E00 | 2,48E-02 | ACADM,CREB3L1,KDM6B,MAPK14,PIK3C2A |
| CLEAR Signaling Pathway | 2,04E00 | 2,11E-02 | ATP6V1A,BMPR2,CREB3L1,MAPK14,PML,PPP3CA |
| Clathrin-mediated Endocytosis Signaling | 2,01E00 | 2,4E-02 | AAK1,MYO6,PIK3C2A,PPP3CA,USP9X |
| HIF1α Signaling | 2,01E00 | 2,4E-02 | HIF1AN,HSP90AA1,PIK3C2A,PKM,PPP3CA |
| Glucocorticoid Receptor Signaling | 1,97E00 | 1,55E-02 | AR,HSP90AA1,IL17RD,MAPK14,PBRM1,PIK3C2A,PPP3CA,SMARCC1,TAF2 |
| Senescence Pathway | 1,95E00 | 2,01E-02 | ANAPC1,BMPR2,MAPK14,PIK3C2A,PML,PPP3CA |
| Hypoxia Signaling in the Cardiovascular System | 1,92E00 | 3,95E-02 | CREB3L1,HIF1AN,HSP90AA1 |
| FLT3 Signaling in Hematopoietic Progenitor Cells | 1,84E00 | 3,66E-02 | CREB3L1,MAPK14,PIK3C2A |
| Role of p14/p19ARF in Tumor Suppression | 1,82E00 | 6,67E-02 | PIK3C2A,SF3A2 |
| BAG2 Signaling Pathway | 1,81E00 | 3,57E-02 | HSP90AA1,MAPK14,PSMD12 |
| FGF Signaling | 1,78E00 | 3,49E-02 | CREB3L1,MAPK14,PIK3C2A |
| AMPK Signaling | 1,75E00 | 2,07E-02 | CREB3L1,MAPK14,PBRM1,PIK3C2A,SMARCC1 |
| S-adenosyl-L-methionine Biosynthesis | 1,73E00 | 3,33E-01 | MAT2A |
| Inhibition of ARE-Mediated mRNA Degradation Pathway | 1,73E00 | 2,47E-02 | CNOT1,MAPK14,PSMD12,ZFP36L2 |
| HOTAIR Regulatory Pathway | 1,72E00 | 2,45E-02 | AR,BRD4,KMT2A,PIK3C2A |
| RANK Signaling in Osteoclasts | 1,71E00 | 3,3E-02 | MAPK14,PIK3C2A,PPP3CA |
| p53 Signaling | 1,63E00 | 3,06E-02 | MAPK14,PIK3C2A,PML |
| UVA-Induced MAPK Signaling | 1,63E00 | 3,06E-02 | MAPK14,PIK3C2A,TNKS2 |
| Sumoylation Pathway | 1,58E00 | 2,91E-02 | AR,PML,RANBP2 |
| Mouse Embryonic Stem Cell Pluripotency | 1,56E00 | 2,88E-02 | BMPR2,MAPK14,PIK3C2A |
| Protein Ubiquitination Pathway | 1,55E00 | 1,83E-02 | ANAPC1,HSP90AA1,PSMD12,USP34,USP9X |
| Paxillin Signaling | 1,53E00 | 2,8E-02 | DOCK1,MAPK14,PIK3C2A |
| RAR Activation | 1,4E00 | 1,95E-02 | MAPK14,PBRM1,PML,SMARCC1 |
| ID1 Signaling Pathway | 1,43E00 | 1,99E-02 | BMPR2,MAPK14,PIK3C2A,ZNF148 |
| Coronavirus Pathogenesis Pathway | 1,41E00 | 1,96E-02 | AR,ATP6V1A,KPNB1,MAPK14 |
| UVB-Induced MAPK Signaling | 1,38E00 | 3,85E-02 | MAPK14,PIK3C2A |
| Endocannabinoid Developing Neuron Pathway | 1,35E00 | 2,36E-02 | CREB3L1,MAPK14,PIK3C2A |
| Role Of Osteoclasts In Rheumatoid Arthritis Signaling Pathway | 1,35E00 | 1,62E-02 | COL5A2,CREB3L1,MAPK14,PIK3C2A,PPP3CA |
| Autophagy | 1,33E00 | 1,85E-02 | ATG2B,CREB3L1,PIK3C2A,PPP3CA |
| EGF Signaling | 1,32E00 | 3,57E-02 | MAPK14,PIK3C2A |
| Synaptogenesis Signaling Pathway | 1,32E00 | 1,59E-02 | CREB3L1,MAP1B,MAPK14,PAFAH1B1,PIK3C2A |
| Role of IL-17A in Arthritis | 1,31E00 | 3,51E-02 | MAPK14,PIK3C2A |
| Neuroinflammation Signaling Pathway | 1,31E00 | 1,58E-02 | BMPR2,CREB3L1,MAPK14,PIK3C2A,PPP3CA |
| Hepatic Fibrosis Signaling Pathway | 1,31E00 | 1,42E-02 | BMPR2,BRD4,CREB3L1,GLIS2,MAPK14,PIK3C2A |

**Table S9.** AUF-1 targets identified by RIP-Sequencing and by catRAPID analysis (**Fig. S6**).

| Transcript_name | Gene_id | Z.score | Discriminative_Power | Interaction_Strength | EF_AUF1 | FDR |
| --- | --- | --- | --- | --- | --- | --- |
| ALDH7A1-006 | ENSG00000164904.15 | 3.75 | 1 | 1 | 1.64 | 0.014021 |
| PSMD12-004 | ENSG00000197170.9 | 3.45 | 1 | 1 | 1.55 | 0.035743 |
| RBMXL1-002 | ENSG00000213516.9 | 4.57 | 1 | 1 | 1.73 | 0.012451 |
| DOCK9-002 | ENSG00000088387.17 | 1.73 | 0.99 | 0.99 | 1.65 | 0.009985 |
| FAM208B-008 | ENSG00000108021.19 | 1.53 | 0.99 | 0.99 | 1.54 | 0.02591 |
| SCAF11-005 | ENSG00000139218.17 | 1.5 | 0.99 | 0.97 | 1.64 | 0.031544 |
| ALDH7A1-013 | ENSG00000164904.15 | 1.15 | 0.98 | 0.97 | 1.64 | 0.014021 |
| COL5A2-002 | ENSG00000204262.11 | 1.28 | 0.98 | 0.99 | 1.69 | 0.026243 |
| EIF4A3-009 | ENSG00000141543.9 | 1.26 | 0.98 | 0.99 | 1.64 | 0.034914 |
| MPLKIP-001 | ENSG00000168303.6 | 1.46 | 0.98 | 0.99 | 1.74 | 0.024103 |
| PRDM11-004 | ENSG00000019485.12 | 1.43 | 0.98 | 0.99 | 1.69 | 0.03023 |
| SMNDC1-002 | ENSG00000119953.12 | 1.19 | 0.98 | 0.98 | 1.73 | 0.014471 |
| ANAPC1-008 | ENSG00000153107.11 | 1 | 0.97 | 0.98 | 1.69 | 0.011066 |
| FAM208B-006 | ENSG00000108021.19 | 1.12 | 0.97 | 0.99 | 1.54 | 0.02591 |
| HDAC2-014 | ENSG00000196591.11 | 1.1 | 0.97 | 0.99 | 1.7 | 0.017493 |
| NEK9-006 | ENSG00000119638.12 | 0.94 | 0.97 | 0.97 | 1.75 | 0.015949 |
| PEG10-004 | ENSG00000242265.5 | 1.06 | 0.97 | 0.98 | 1.58 | 0.017043 |
| PREP-002 | ENSG00000085377.13 | 1.07 | 0.97 | 0.97 | 1.84 | 0.005009 |
| RAP2C-003 | ENSG00000123728.9 | 0.96 | 0.97 | 0.98 | 1.97 | 0.001002 |
| SF1-011 | ENSG00000168066.20 | 0.97 | 0.97 | 0.96 | 1.54 | 0.020991 |
| SORBS3-024 | ENSG00000120896.13 | 0.95 | 0.97 | 0.97 | 2.29 | 0.000841 |
| SORBS3-013 | ENSG00000120896.13 | 0.92 | 0.97 | 0.98 | 2.29 | 0.000841 |
| UBXN7-002 | ENSG00000163960.11 | 1.08 | 0.97 | 0.97 | 1.6 | 0.009406 |
| CORO1C-018 | ENSG00000110880.10 | 0.85 | 0.96 | 0.96 | 1.73 | 0.003582 |
| KPNB1-006 | ENSG00000108424.9 | 0.79 | 0.96 | 0.96 | 1.53 | 0.037058 |
| LPIN2-002 | ENSG00000101577.9 | 0.85 | 0.96 | 0.96 | 1.53 | 0.035849 |
| MKI67-004 | ENSG00000148773.12 | 0.8 | 0.96 | 0.95 | 1.57 | 0.021889 |
| NCOR2-009 | ENSG00000196498.13 | 0.82 | 0.96 | 0.96 | 1.94 | 0.01749 |
| XIAP-001 | ENSG00000101966.12 | 0.84 | 0.96 | 0.97 | 1.53 | 0.038949 |
| ZNF587-004 | ENSG00000198466.11 | 0.85 | 0.96 | 0.98 | 1.56 | 0.035882 |
| DCAF7-004 | ENSG00000136485.14 | 0.76 | 0.95 | 0.95 | 1.64 | 0.010168 |
| LMNB1-004 | ENSG00000113368.11 | 0.76 | 0.95 | 0.96 | 1.81 | 0.00929 |
| MYOF-001 | ENSG00000138119.16 | 0.76 | 0.95 | 0.92 | 1.95 | 0.001451 |
| PAPSS1-006 | ENSG00000138801.8 | 0.76 | 0.95 | 0.92 | 1.67 | 0.048926 |
| MUC1-018 | ENSG00000185499.16 | 0.67 | 0.94 | 0.93 | 3.37 | 1.81E-06 |
| PBRM1-013 | ENSG00000163939.18 | 0.66 | 0.94 | 0.93 | 1.63 | 0.014761 |
| SF3B3-014 | ENSG00000189091.12 | 0.68 | 0.94 | 0.93 | 1.74 | 0.027771 |
| ARF3-012 | ENSG00000134287.9 | 0.64 | 0.93 | 0.95 | 1.53 | 0.024611 |
| SF1-012 | ENSG00000168066.20 | 0.61 | 0.92 | 0.93 | 1.54 | 0.020991 |
| VPS41-010 | ENSG00000006715.15 | 0.6 | 0.92 | 0.92 | 1.92 | 0.002647 |
| APBB2-018 | ENSG00000163697.16 | 0.55 | 0.91 | 0.87 | 1.54 | 0.020718 |
| ATIC-016 | ENSG00000138363.14 | 0.52 | 0.91 | 0.91 | 1.82 | 0.011266 |
| CORO1C-017 | ENSG00000110880.10 | 0.53 | 0.91 | 0.92 | 1.73 | 0.003582 |
| LMNB1-006 | ENSG00000113368.11 | 0.53 | 0.91 | 0.91 | 1.81 | 0.00929 |
| LPP-004 | ENSG00000145012.12 | 0.56 | 0.91 | 0.88 | 1.6 | 0.013544 |
| MACF1-025 | ENSG00000127603.23 | 0.56 | 0.91 | 0.9 | 1.61 | 0.014615 |
| RREB1-011 | ENSG00000124782.19 | 0.54 | 0.91 | 0.87 | 1.57 | 0.042637 |
| SRRM2-028 | ENSG00000167978.16 | 0.56 | 0.91 | 0.95 | 1.96 | 8.29E-05 |
| VPS37C-003 | ENSG00000167987.10 | 0.54 | 0.91 | 0.91 | 1.99 | 0.01312 |
| DDX17-007 | ENSG00000100201.19 | 0.48 | 0.89 | 0.87 | 2.13 | 6.31E-06 |
| AHNAK-001 | ENSG00000124942.13 | 0.45 | 0.88 | 0.84 | 1.65 | 0.004293 |
| NIPBL-003 | ENSG00000164190.16 | 0.47 | 0.88 | 0.89 | 1.59 | 0.029242 |
| SF3B3-018 | ENSG00000189091.12 | 0.47 | 0.88 | 0.9 | 1.74 | 0.027771 |
| ATXN1L-003 | ENSG00000224470.7 | 0.44 | 0.87 | 0.95 | 1.56 | 0.039925 |
| CORO1C-014 | ENSG00000110880.10 | 0.42 | 0.85 | 0.8 | 1.73 | 0.003582 |
| HLCS-004 | ENSG00000159267.14 | 0.41 | 0.85 | 0.85 | 1.54 | 0.045645 |
| TMED7-004 | ENSG00000134970.13 | 0.41 | 0.85 | 0.89 | 2 | 0.001526 |
| TNRC6B-004 | ENSG00000100354.20 | 0.41 | 0.85 | 0.94 | 1.55 | 0.026788 |
| GRINA-011 | ENSG00000178719.16 | 0.39 | 0.84 | 0.79 | 1.67 | 0.014701 |
| GRINA-009 | ENSG00000178719.16 | 0.39 | 0.84 | 0.87 | 1.67 | 0.014701 |
| PACS1-006 | ENSG00000175115.11 | 0.39 | 0.84 | 0.87 | 1.72 | 0.0035 |
| SMC1A-002 | ENSG00000072501.17 | 0.39 | 0.84 | 0.79 | 2.09 | 0.000554 |
| COPB2-005 | ENSG00000184432.9 | 0.34 | 0.83 | 0.77 | 1.87 | 0.018715 |
| KDM6B-005 | ENSG00000132510.10 | 0.34 | 0.83 | 0.97 | 3.2 | 0.000139 |
| POGZ-018 | ENSG00000143442.21 | 0.34 | 0.83 | 0.86 | 1.8 | 0.001353 |
| ZFHX4-008 | ENSG00000091656.15 | 0.35 | 0.83 | 0.8 | 1.92 | 0.000304 |
| ATP6V1A-005 | ENSG00000114573.9 | 0.32 | 0.81 | 0.82 | 1.62 | 0.042774 |
| CNOT1-009 | ENSG00000125107.16 | 0.33 | 0.81 | 0.85 | 1.82 | 0.000347 |
| DDX3X-003 | ENSG00000215301.9 | 0.32 | 0.81 | 0.84 | 1.75 | 0.018266 |
| SDCBP-013 | ENSG00000137575.11 | 0.31 | 0.81 | 0.84 | 1.66 | 0.011202 |
| SUCLA2-010 | ENSG00000136143.14 | 0.32 | 0.81 | 0.77 | 1.78 | 0.015724 |
| WNK1-016 | ENSG00000060237.16 | 0.32 | 0.81 | 0.79 | 1.68 | 0.003319 |
| ZNF462-010 | ENSG00000148143.12 | 0.34 | 0.81 | 0.87 | 1.66 | 0.006012 |
| CNOT1-023 | ENSG00000125107.16 | 0.29 | 0.8 | 0.83 | 1.82 | 0.000347 |
| DOCK9-012 | ENSG00000088387.17 | 0.3 | 0.8 | 0.75 | 1.65 | 0.009985 |
| PRR14L-007 | ENSG00000183530.13 | 0.29 | 0.8 | 0.77 | 1.57 | 0.01896 |
| TRIP12-009 | ENSG00000153827.13 | 0.31 | 0.8 | 0.79 | 1.76 | 0.00196 |
| MED20-007 | ENSG00000124641.14 | 0.26 | 0.79 | 0.82 | 1.6 | 0.032051 |
| FRMD6-012 | ENSG00000139926.15 | 0.24 | 0.77 | 0.82 | 1.55 | 0.019485 |
| PBRM1-018 | ENSG00000163939.18 | 0.24 | 0.77 | 0.83 | 1.63 | 0.014761 |
| SRRM2-029 | ENSG00000167978.16 | 0.23 | 0.77 | 0.77 | 1.96 | 8.29E-05 |
| MLXIP-006 | ENSG00000175727.13 | 0.21 | 0.76 | 0.83 | 2.15 | 0.002861 |
| NUP214-006 | ENSG00000126883.16 | 0.23 | 0.76 | 0.8 | 1.72 | 0.032662 |
| SF3B3-008 | ENSG00000189091.12 | 0.22 | 0.76 | 0.78 | 1.74 | 0.027771 |
| SORBS3-006 | ENSG00000120896.13 | 0.23 | 0.76 | 0.8 | 2.29 | 0.000841 |
