# Supplementary material for: Expression of targets of the RNA-binding protein AUF-1 in human airway epithelium indicates its role in cellular senescence and inflammation
Source: Front Immunol. 2023 Jul 7;14:1192028. doi: 10.3389/fimmu.2023.1192028 (PMC10360199; doi:10.3389/fimmu.2023.1192028)
Supplement: Supplementary file 2 [file DataSheet_2.docx]

**Table S4.** Full gene list of AUF-1-associated transcripts (n=494) identified by RIP-Seq analysis in unstimulated cells from the BEAS-2B line. EF, Enriched Factor; FDR, False Discovery Rate.

| GENE_SYMBOL | EF | FDR |
| --- | --- | --- |
| PRR36 | 5,73 | 0,020203 |
| GLIS2 | 4,95 | 0,00745 |
| ZNF385A | 4,4 | 5,15E-05 |
| TCF7L1 | 4,14 | 0,003217 |
| PIANP | 3,52 | 0,000871 |
| MBD6 | 3,42 | 2,49E-09 |
| MUC1 | 3,37 | 1,81E-06 |
| FOXP4 | 3,27 | 7,46E-05 |
| KDM6B | 3,2 | 0,000139 |
| FBRSL1 | 3,09 | 0,000336 |
| C1orf226 | 2,86 | 0,039358 |
| AP001972.5 | 2,84 | 0,032884 |
| STX1B | 2,83 | 0,012534 |
| CRTC1 | 2,73 | 0,001314 |
| AL513165.1 | 2,66 | 0,034496 |
| ATXN2L | 2,6 | 1,37E-08 |
| RNF44 | 2,6 | 0,000146 |
| IL17RD | 2,58 | 0,026678 |
| KIAA1522 | 2,58 | 1,23E-08 |
| HIVEP3 | 2,47 | 0,000702 |
| RNF165 | 2,46 | 0,026659 |
| MNT | 2,45 | 0,000654 |
| BICRA | 2,43 | 0,011555 |
| SYNPO | 2,43 | 8,11E-07 |
| RIN3 | 2,42 | 0,005946 |
| ZBTB7A | 2,42 | 0,001117 |
| AL158212.3 | 2,38 | 0,020718 |
| ZFHX2 | 2,37 | 0,04323 |
| AL365181.3 | 2,3 | 0,004542 |
| ZNF697 | 2,3 | 0,043455 |
| SORBS3 | 2,29 | 0,000841 |
| AKAP12 | 2,27 | 0,000177 |
| LINC01963 | 2,25 | 0,011995 |
| ZNF555 | 2,25 | 0,002913 |
| PPP1R13L | 2,24 | 0,000906 |
| NFIX | 2,19 | 0,000846 |
| CREB3L1 | 2,18 | 0,038525 |
| TGFB1 | 2,16 | 0,003603 |
| EGR1 | 2,15 | 0,00016 |
| FRMD4B | 2,15 | 0,047394 |
| MLXIP | 2,15 | 0,002861 |
| PER1 | 2,15 | 0,00825 |
| AC037459.3 | 2,14 | 0,032596 |
| CRNKL1 | 2,14 | 0,005231 |
| DDX17 | 2,13 | 6,31E-06 |
| RERE | 2,13 | 2,14E-05 |


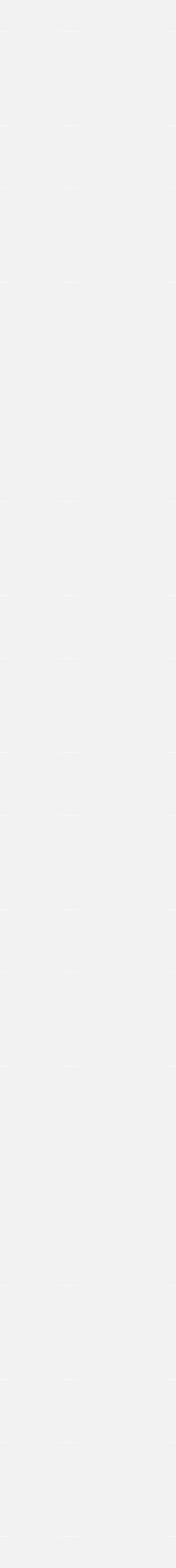


| AC012513.3 | 2,12 | 0,037906 |
| --- | --- | --- |
| CPNE8 | 2,11 | 0,019199 |
| SMC1A | 2,09 | 0,000554 |
| UBR5 | 2,09 | 1,57E-05 |
| FAM98B | 2,07 | 0,002364 |
| SF3A2 | 2,07 | 0,004451 |
| CAMTA2 | 2,06 | 0,004478 |
| NFATC2IP | 2,06 | 4,41E-05 |
| SVEP1 | 2,06 | 0,010826 |
| WDR3 | 2,06 | 0,007161 |
| ZXDA | 2,06 | 0,033232 |
| CDC42EP1 | 2,05 | 0,003648 |
| POLR1B | 2,05 | 0,001552 |
| SCARNA7 | 2,05 | 0,001451 |
| TMEM178B | 2,05 | 0,017907 |
| LIG4 | 2,04 | 0,012687 |
| NPTXR | 2,03 | 0,016856 |
| YLPM1 | 2,03 | 0,0003 |
| FAM120C | 2,02 | 0,013125 |
| IRS2 | 2,01 | 0,003319 |
| BCL3 | 2 | 0,016054 |
| DAGLA | 2 | 0,017176 |
| RAB14 | 2 | 0,000195 |
| SUPT16H | 2 | 0,009683 |
| TMED7 | 2 | 0,001526 |
| ZMIZ2 | 2 | 0,003344 |
| ZNF888 | 2 | 0,027102 |
| CBX5 | 1,99 | 6,46E-05 |
| SAMD9L | 1,99 | 0,019229 |
| VPS37C | 1,99 | 0,01312 |
| ZNF221 | 1,99 | 0,033232 |
| COL12A1 | 1,98 | 0,002492 |
| HNRNPH2 | 1,98 | 0,009816 |
| MARCKS | 1,98 | 0,00108 |
| NECTIN1 | 1,98 | 0,003359 |
| SMC3 | 1,98 | 0,006401 |
| SMG8 | 1,98 | 0,005702 |
| SON | 1,98 | 0,000719 |
| HSD17B4 | 1,97 | 0,012356 |
| RAP2C | 1,97 | 0,001002 |
| TET3 | 1,97 | 0,00108 |
| ARL6IP6 | 1,96 | 0,001166 |
| BRD4 | 1,96 | 0,001236 |
| POM121C | 1,96 | 0,000586 |
| SRRM2 | 1,96 | 8,29E-05 |
| TOP1 | 1,96 | 0,007066 |
| AL031587.5 | 1,95 | 0,011276 |
| EPN1 | 1,95 | 0,005678 |
| MAST4 | 1,95 | 0,04438 |
| MYOF | 1,95 | 0,001451 |


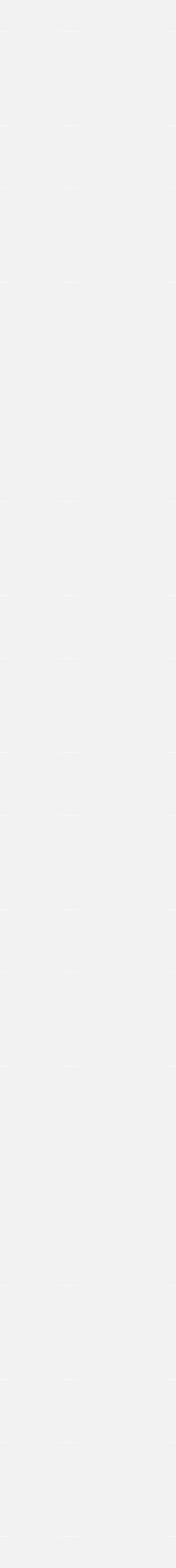


| DHX36 | 1,94 | 0,009784 |
| --- | --- | --- |
| NCOR2 | 1,94 | 0,01749 |
| SLFN11 | 1,93 | 0,002032 |
| TMEM8B | 1,93 | 0,030496 |
| ACTR6 | 1,92 | 0,038294 |
| FOXJ2 | 1,92 | 0,001315 |
| NATD1 | 1,92 | 0,007202 |
| NUP160 | 1,92 | 0,024106 |
| PSAT1 | 1,92 | 0,004771 |
| VPS41 | 1,92 | 0,002647 |
| ZFHX4 | 1,92 | 0,000304 |
| ADAMTSL4 | 1,91 | 0,015213 |
| EIF5B | 1,91 | 0,013755 |
| ELFN2 | 1,91 | 0,022742 |
| TRIP4 | 1,91 | 0,028566 |
| CCDC120 | 1,9 | 0,048041 |
| DCAF1 | 1,9 | 0,007427 |
| LEMD3 | 1,9 | 0,007714 |
| MCCC2 | 1,9 | 0,009406 |
| USP9X | 1,9 | 0,000813 |
| BRWD3 | 1,89 | 0,001032 |
| COL4A6 | 1,89 | 0,048614 |
| DDX20 | 1,89 | 0,024583 |
| EFTUD2 | 1,89 | 0,018566 |
| MYO5A | 1,89 | 0,000363 |
| PLAGL1 | 1,89 | 0,007338 |
| SART3 | 1,89 | 0,004504 |
| GRAMD1B | 1,88 | 0,012766 |
| SF3B4 | 1,88 | 0,004953 |
| TRIM13 | 1,88 | 0,00447 |
| CEBPZ | 1,87 | 0,006399 |
| CLTC | 1,87 | 0,004478 |
| COPB2 | 1,87 | 0,018715 |
| GEMIN5 | 1,87 | 0,027269 |
| NKRF | 1,87 | 0,016649 |
| PLAGL2 | 1,87 | 0,002967 |
| AR | 1,86 | 0,009351 |
| GTF3C1 | 1,86 | 0,012556 |
| KLHL28 | 1,86 | 0,024761 |
| MTHFD1 | 1,86 | 0,022111 |
| NCAPD3 | 1,86 | 0,007964 |
| NPRL3 | 1,86 | 0,011963 |
| PIK3R4 | 1,86 | 0,020724 |
| WDR7 | 1,86 | 0,013386 |
| AASDH | 1,85 | 0,042012 |
| ANKFY1 | 1,85 | 0,00301 |
| CHD9 | 1,85 | 0,001472 |
| CTPS1 | 1,85 | 0,007537 |
| DLST | 1,85 | 0,001575 |
| FBXO30 | 1,85 | 0,004943 |


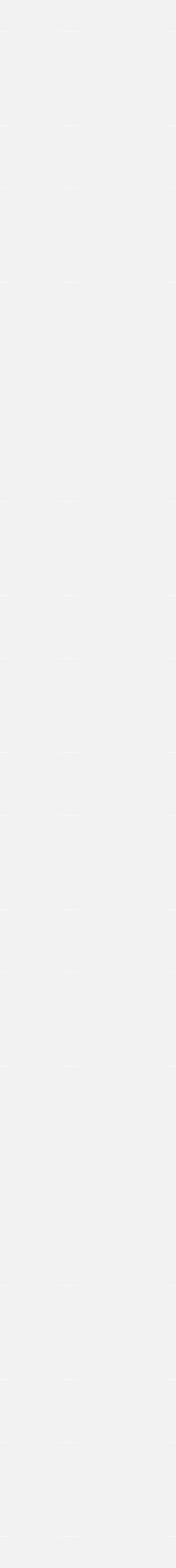


| MDGA1 | 1,85 | 0,043779 |
| --- | --- | --- |
| TRIM5 | 1,85 | 0,018156 |
| ZNF607 | 1,85 | 0,025731 |
| AARS | 1,84 | 0,011116 |
| ARID2 | 1,84 | 0,002333 |
| HNRNPM | 1,84 | 0,010043 |
| MMP24OS | 1,84 | 0,005863 |
| NEURL1B | 1,84 | 0,002201 |
| PREP | 1,84 | 0,005009 |
| PRICKLE2 | 1,84 | 0,005593 |
| PROSER3 | 1,84 | 0,011712 |
| AC004943.2 | 1,83 | 0,043402 |
| FAM160B1 | 1,83 | 0,003045 |
| FIGN | 1,83 | 0,005163 |
| SEC24D | 1,83 | 0,01381 |
| THRA | 1,83 | 0,009064 |
| ATIC | 1,82 | 0,011266 |
| CAMK1D | 1,82 | 0,009148 |
| CNOT1 | 1,82 | 0,000347 |
| IBA57 | 1,82 | 0,040616 |
| KBTBD7 | 1,82 | 0,040506 |
| RECQL | 1,82 | 0,030015 |
| RNF20 | 1,82 | 0,030753 |
| RPAP3 | 1,82 | 0,020991 |
| VAPB | 1,82 | 0,000414 |
| ZNF219 | 1,82 | 0,035867 |
| CRYBG3 | 1,81 | 0,019199 |
| LMNB1 | 1,81 | 0,00929 |
| NACC2 | 1,81 | 0,009609 |
| PIK3C2A | 1,81 | 0,005266 |
| RUNX3 | 1,81 | 0,032767 |
| ZBTB21 | 1,81 | 0,004685 |
| BMS1 | 1,8 | 0,033384 |
| DLD | 1,8 | 0,012046 |
| NCOA7 | 1,8 | 0,002637 |
| PLRG1 | 1,8 | 0,033329 |
| POGZ | 1,8 | 0,001353 |
| ZNF106 | 1,8 | 0,000631 |
| ZNF551 | 1,8 | 0,021424 |
| AMIGO2 | 1,79 | 0,027058 |
| ASXL2 | 1,79 | 0,002797 |
| DENND4C | 1,79 | 0,015438 |
| MYORG | 1,79 | 0,042896 |
| NHS | 1,79 | 0,034874 |
| TSC22D4 | 1,79 | 0,024377 |
| CCNK | 1,78 | 0,018413 |
| DOCK7 | 1,78 | 0,042714 |
| G3BP2 | 1,78 | 0,002332 |
| SEC23IP | 1,78 | 0,037567 |
| SUCLA2 | 1,78 | 0,015724 |


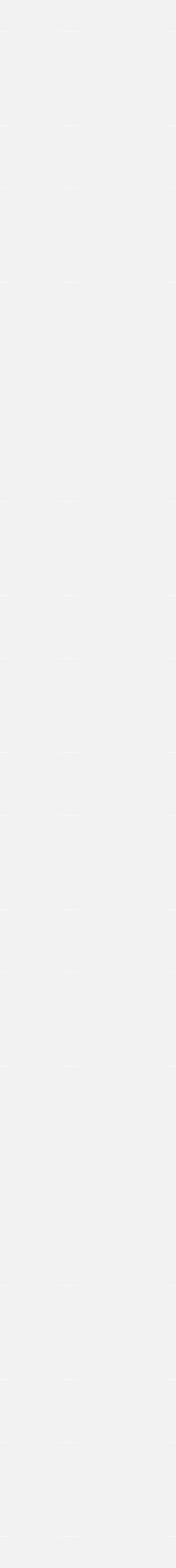


| TOPORS | 1,78 | 0,014124 |
| --- | --- | --- |
| UBR3 | 1,78 | 0,016497 |
| WDFY3 | 1,78 | 0,001371 |
| DIO2 | 1,77 | 0,007614 |
| DOCK4 | 1,77 | 0,017798 |
| ERC1 | 1,77 | 0,009031 |
| ERF | 1,77 | 0,013416 |
| MCM10 | 1,77 | 0,026995 |
| MTR | 1,77 | 0,007964 |
| NOL11 | 1,77 | 0,044104 |
| POLR1A | 1,77 | 0,028233 |
| POM121 | 1,77 | 0,006086 |
| TRERF1 | 1,77 | 0,005063 |
| TRPS1 | 1,77 | 0,02826 |
| TSPYL1 | 1,77 | 0,001935 |
| YTHDC2 | 1,77 | 0,045855 |
| ARFGEF3 | 1,76 | 0,025196 |
| CSTF2T | 1,76 | 0,020999 |
| DDX1 | 1,76 | 0,029098 |
| DMXL1 | 1,76 | 0,003715 |
| FASTKD2 | 1,76 | 0,042233 |
| TRIP12 | 1,76 | 0,00196 |
| WDR47 | 1,76 | 0,024643 |
| ACADM | 1,75 | 0,010333 |
| ATG2B | 1,75 | 0,003324 |
| BAG3 | 1,75 | 0,019395 |
| DDX3X | 1,75 | 0,018266 |
| ERCC6L | 1,75 | 0,028489 |
| HSP90AA1 | 1,75 | 0,023752 |
| MINPP1 | 1,75 | 0,042958 |
| NEK9 | 1,75 | 0,015949 |
| SYT16 | 1,75 | 0,044134 |
| TOP2B | 1,75 | 0,007066 |
| WDR36 | 1,75 | 0,04438 |
| COPA | 1,74 | 0,041701 |
| DHX40 | 1,74 | 0,019459 |
| DMXL2 | 1,74 | 0,021561 |
| DOP1A | 1,74 | 0,037632 |
| EPG5 | 1,74 | 0,016524 |
| GIT2 | 1,74 | 0,013925 |
| KLHL24 | 1,74 | 0,013386 |
| MPLKIP | 1,74 | 0,024103 |
| POLR3A | 1,74 | 0,017697 |
| PTK2B | 1,74 | 0,037832 |
| SF3B3 | 1,74 | 0,027771 |
| URB1 | 1,74 | 0,01869 |
| ADGRL1 | 1,73 | 0,023475 |
| BEND3 | 1,73 | 0,039406 |
| CORO1C | 1,73 | 0,003582 |
| CRKL | 1,73 | 0,002197 |


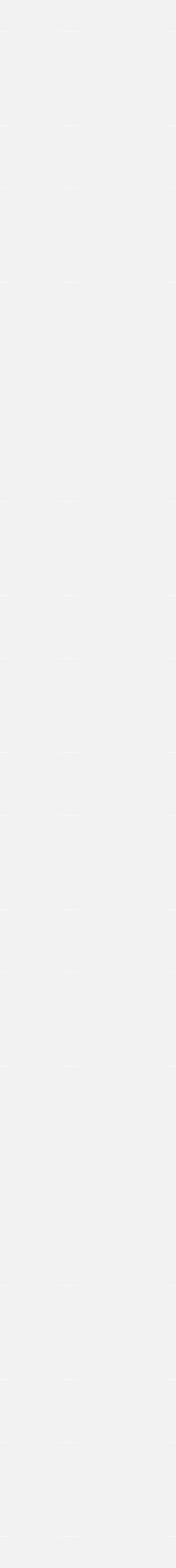


| EED | 1,73 | 0,028789 |
| --- | --- | --- |
| FBXO11 | 1,73 | 0,017493 |
| GCH1 | 1,73 | 0,020541 |
| IREB2 | 1,73 | 0,004428 |
| PAICS | 1,73 | 0,002123 |
| RBMXL1 | 1,73 | 0,012451 |
| RNF111 | 1,73 | 0,039845 |
| SMNDC1 | 1,73 | 0,014471 |
| CHD1 | 1,72 | 0,022644 |
| FAM222B | 1,72 | 0,033972 |
| MAMLD1 | 1,72 | 0,020665 |
| NPEPPS | 1,72 | 0,012968 |
| NUP214 | 1,72 | 0,032662 |
| PACS1 | 1,72 | 0,0035 |
| PLS3 | 1,72 | 0,027236 |
| PTPN14 | 1,72 | 0,001692 |
| AP1G1 | 1,71 | 0,005593 |
| CPSF7 | 1,71 | 0,004825 |
| DNAJC13 | 1,71 | 0,011256 |
| MED12 | 1,71 | 0,00733 |
| MIB1 | 1,71 | 0,003195 |
| NHLRC2 | 1,71 | 0,013803 |
| RAB3B | 1,71 | 0,001753 |
| RALGAPB | 1,71 | 0,012032 |
| RAPH1 | 1,71 | 0,002963 |
| STK35 | 1,71 | 0,008742 |
| ZBTB10 | 1,71 | 0,016877 |
| DNMBP | 1,7 | 0,030715 |
| DOCK5 | 1,7 | 0,002992 |
| GPATCH8 | 1,7 | 0,020406 |
| HDAC2 | 1,7 | 0,017493 |
| PDCD4 | 1,7 | 0,019496 |
| WDR11 | 1,7 | 0,026166 |
| ABCD3 | 1,69 | 0,013297 |
| ANAPC1 | 1,69 | 0,011066 |
| COL5A2 | 1,69 | 0,026243 |
| DYNC1H1 | 1,69 | 0,011245 |
| FAM160A1 | 1,69 | 0,030753 |
| HEATR6 | 1,69 | 0,031621 |
| KIAA1109 | 1,69 | 0,013803 |
| PRDM11 | 1,69 | 0,03023 |
| SAMD4B | 1,69 | 0,012752 |
| VIRMA | 1,69 | 0,024321 |
| ATP6V1B2 | 1,68 | 0,024643 |
| CTNNA1 | 1,68 | 0,037269 |
| DDX46 | 1,68 | 0,033733 |
| DNMT1 | 1,68 | 0,045856 |
| FBXO38 | 1,68 | 0,046632 |
| GLCCI1 | 1,68 | 0,021442 |
| MAST3 | 1,68 | 0,022542 |


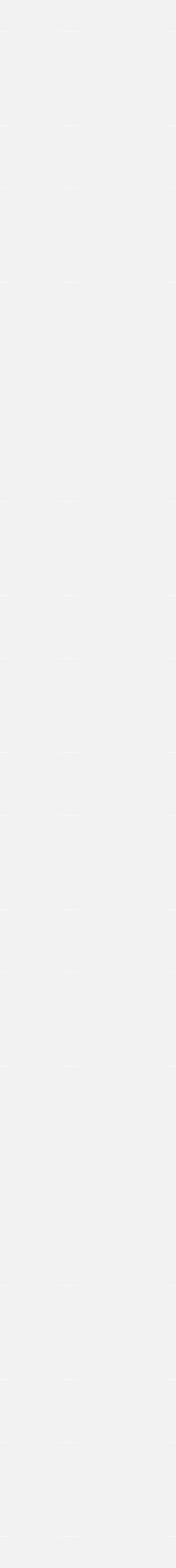


| MYO6 | 1,68 | 0,045818 |
| --- | --- | --- |
| NF1 | 1,68 | 0,006862 |
| SRGAP1 | 1,68 | 0,014568 |
| TEAD3 | 1,68 | 0,023595 |
| TUG1 | 1,68 | 0,010064 |
| USP14 | 1,68 | 0,026166 |
| WNK1 | 1,68 | 0,003319 |
| BLMH | 1,67 | 0,011218 |
| GRINA | 1,67 | 0,014701 |
| ITPR2 | 1,67 | 0,037847 |
| MYO1E | 1,67 | 0,044752 |
| NOL9 | 1,67 | 0,023025 |
| NT5DC3 | 1,67 | 0,00686 |
| PAK4 | 1,67 | 0,044413 |
| PAPSS1 | 1,67 | 0,048926 |
| PIKFYVE | 1,67 | 0,007311 |
| RAP1GAP2 | 1,67 | 0,028754 |
| TRAF6 | 1,67 | 0,029498 |
| ZNF148 | 1,67 | 0,006623 |
| APOBEC3C | 1,66 | 0,023025 |
| APOOL | 1,66 | 0,030843 |
| ATXN1 | 1,66 | 0,017697 |
| CYB5RL | 1,66 | 0,04387 |
| DDHD1 | 1,66 | 0,018784 |
| EIF4G3 | 1,66 | 0,005624 |
| LRBA | 1,66 | 0,010908 |
| MAP3K9 | 1,66 | 0,031094 |
| MAVS | 1,66 | 0,027521 |
| MED13 | 1,66 | 0,004775 |
| N4BP2 | 1,66 | 0,044454 |
| SDCBP | 1,66 | 0,011202 |
| TCEAL9 | 1,66 | 0,016357 |
| TRIM14 | 1,66 | 0,014761 |
| ZNF462 | 1,66 | 0,006012 |
| AHNAK | 1,65 | 0,004293 |
| DOCK9 | 1,65 | 0,009985 |
| MTHFR | 1,65 | 0,043674 |
| NARS | 1,65 | 0,049028 |
| NIF3L1 | 1,65 | 0,026166 |
| PDS5B | 1,65 | 0,017191 |
| PFKFB2 | 1,65 | 0,048243 |
| PHIP | 1,65 | 0,037967 |
| RANBP6 | 1,65 | 0,04022 |
| RBM15 | 1,65 | 0,026375 |
| SRP54 | 1,65 | 0,049409 |
| TBL1X | 1,65 | 0,026678 |
| TULP3 | 1,65 | 0,036366 |
| ZFP36L2 | 1,65 | 0,042908 |
| ALDH7A1 | 1,64 | 0,014021 |
| CBLL1 | 1,64 | 0,048504 |


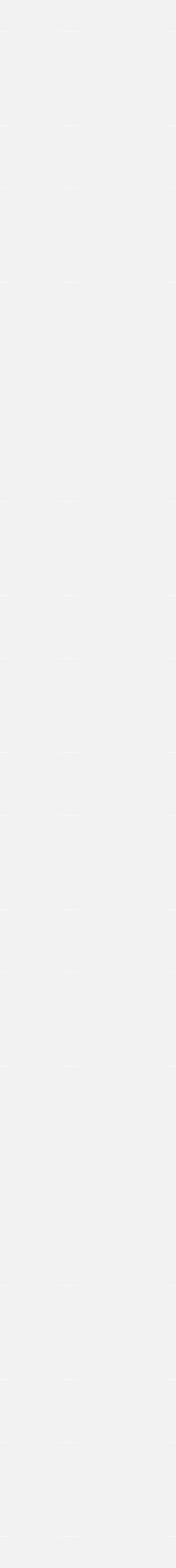


| CCDC82 | 1,64 | 0,043851 |
| --- | --- | --- |
| DCAF7 | 1,64 | 0,010168 |
| EIF4A3 | 1,64 | 0,034914 |
| FAM168A | 1,64 | 0,006668 |
| KIF24 | 1,64 | 0,028489 |
| MDN1 | 1,64 | 0,041353 |
| PML | 1,64 | 0,023736 |
| PRMT6 | 1,64 | 0,048848 |
| RANBP2 | 1,64 | 0,019229 |
| SCAF11 | 1,64 | 0,031544 |
| SETX | 1,64 | 0,018609 |
| TAF1 | 1,64 | 0,01499 |
| TANC2 | 1,64 | 0,004497 |
| ZBED5 | 1,64 | 0,045056 |
| MPHOSPH8 | 1,63 | 0,042752 |
| MYNN | 1,63 | 0,048625 |
| PALLD | 1,63 | 0,00675 |
| PBRM1 | 1,63 | 0,014761 |
| SBNO1 | 1,63 | 0,013508 |
| SEMA3A | 1,63 | 0,026078 |
| ZNF765 | 1,63 | 0,033282 |
| ZNF845 | 1,63 | 0,045805 |
| ZSWIM6 | 1,63 | 0,012968 |
| ATP6V1A | 1,62 | 0,042774 |
| ERCC6 | 1,62 | 0,028489 |
| MAP3K1 | 1,62 | 0,024063 |
| METTL16 | 1,62 | 0,01558 |
| MPP5 | 1,62 | 0,019258 |
| NCOR1 | 1,62 | 0,02023 |
| SELENON | 1,62 | 0,016313 |
| SIK3 | 1,62 | 0,019111 |
| SPATA13 | 1,62 | 0,049615 |
| SRPRA | 1,62 | 0,022284 |
| WEE1 | 1,62 | 0,035551 |
| BIRC6 | 1,61 | 0,028575 |
| HELLS | 1,61 | 0,031652 |
| IGF1R | 1,61 | 0,016693 |
| LIG3 | 1,61 | 0,032138 |
| MACF1 | 1,61 | 0,014615 |
| MAT2A | 1,61 | 0,038683 |
| NCOA6 | 1,61 | 0,016198 |
| PDP1 | 1,61 | 0,026678 |
| RTCB | 1,61 | 0,042896 |
| RUNX1 | 1,61 | 0,021062 |
| SPRED2 | 1,61 | 0,0298 |
| TSC22D2 | 1,61 | 0,020438 |
| USP34 | 1,61 | 0,009784 |
| DDX21 | 1,6 | 0,043779 |
| FTO | 1,6 | 0,049895 |
| LPP | 1,6 | 0,013544 |


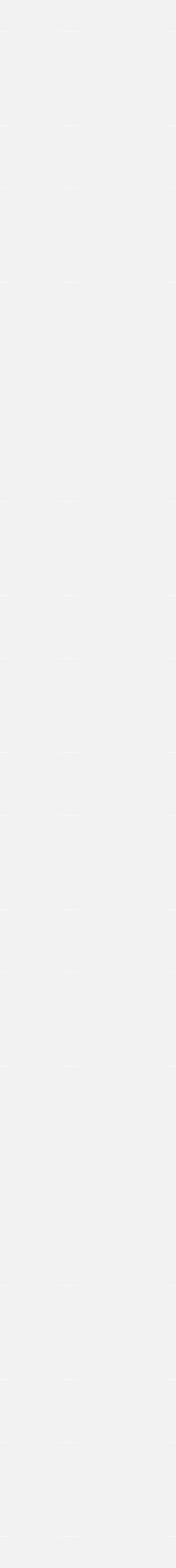


| MED20 | 1,6 | 0,032051 |
| --- | --- | --- |
| NAV2 | 1,6 | 0,027014 |
| PDE1C | 1,6 | 0,010379 |
| PKM | 1,6 | 0,028566 |
| QSER1 | 1,6 | 0,032528 |
| UBXN7 | 1,6 | 0,009406 |
| ZNF281 | 1,6 | 0,043779 |
| ABL2 | 1,59 | 0,011266 |
| C2CD3 | 1,59 | 0,029498 |
| CLASP1 | 1,59 | 0,023914 |
| FN1 | 1,59 | 0,031621 |
| ICE1 | 1,59 | 0,025196 |
| LRPPRC | 1,59 | 0,048861 |
| NIPBL | 1,59 | 0,029242 |
| NRIP1 | 1,59 | 0,015232 |
| PAK2 | 1,59 | 0,010016 |
| PHF20 | 1,59 | 0,023516 |
| RGP1 | 1,59 | 0,017329 |
| TAF2 | 1,59 | 0,047451 |
| UBR4 | 1,59 | 0,010492 |
| ADAT1 | 1,58 | 0,037967 |
| GLI3 | 1,58 | 0,034866 |
| GNA12 | 1,58 | 0,037128 |
| KPNA6 | 1,58 | 0,043402 |
| MAP1B | 1,58 | 0,025353 |
| MAPK14 | 1,58 | 0,030753 |
| NEU3 | 1,58 | 0,041545 |
| PAN3 | 1,58 | 0,020557 |
| PEG10 | 1,58 | 0,017043 |
| RFX7 | 1,58 | 0,022644 |
| SIPA1L2 | 1,58 | 0,046799 |
| SMAD3 | 1,58 | 0,014242 |
| THUMPD1 | 1,58 | 0,038338 |
| TP53BP2 | 1,58 | 0,037967 |
| USP40 | 1,58 | 0,043165 |
| BHLHE40 | 1,57 | 0,04742 |
| CRYBG1 | 1,57 | 0,026855 |
| MIEF1 | 1,57 | 0,032281 |
| MKI67 | 1,57 | 0,021889 |
| PAFAH1B1 | 1,57 | 0,011198 |
| PJA2 | 1,57 | 0,04553 |
| PRR14L | 1,57 | 0,01896 |
| RAB11FIP1 | 1,57 | 0,020592 |
| RREB1 | 1,57 | 0,042637 |
| STXBP1 | 1,57 | 0,02617 |
| SYNCRIP | 1,57 | 0,048277 |
| TMPO | 1,57 | 0,020991 |
| AAK1 | 1,56 | 0,030407 |
| ATXN1L | 1,56 | 0,039925 |
| DICER1 | 1,56 | 0,026375 |


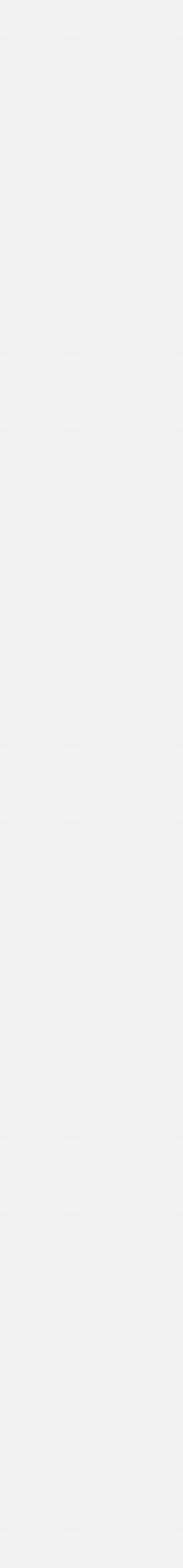


| GBF1 | 1,56 | 0,039763 |
| --- | --- | --- |
| GJA1 | 1,56 | 0,043402 |
| PCNX1 | 1,56 | 0,024485 |
| PXN | 1,56 | 0,023271 |
| ZNF587 | 1,56 | 0,035882 |
| ANKRD17 | 1,55 | 0,049895 |
| ARHGEF7 | 1,55 | 0,045818 |
| FRMD6 | 1,55 | 0,019485 |
| MECP2 | 1,55 | 0,045722 |
| PSMD12 | 1,55 | 0,035743 |
| RBPJ | 1,55 | 0,028668 |
| TFCP2 | 1,55 | 0,046532 |
| TNRC6B | 1,55 | 0,026788 |
| TUBGCP4 | 1,55 | 0,042235 |
| APBB2 | 1,54 | 0,020718 |
| CAND1 | 1,54 | 0,049007 |
| FAM208B | 1,54 | 0,02591 |
| HIF1AN | 1,54 | 0,021424 |
| HLCS | 1,54 | 0,045645 |
| NSD3 | 1,54 | 0,023881 |
| SF1 | 1,54 | 0,020991 |
| ARF3 | 1,53 | 0,024611 |
| ARHGAP21 | 1,53 | 0,030715 |
| HPS3 | 1,53 | 0,035819 |
| KMT2A | 1,53 | 0,035849 |
| KPNB1 | 1,53 | 0,037058 |
| LPIN2 | 1,53 | 0,035849 |
| NR2C2 | 1,53 | 0,036826 |
| PSME4 | 1,53 | 0,023595 |
| SMARCC1 | 1,53 | 0,043732 |
| SWAP70 | 1,53 | 0,029709 |
| TNKS2 | 1,53 | 0,028566 |
| VPS26A | 1,53 | 0,049267 |
| XIAP | 1,53 | 0,038949 |
| ZNF609 | 1,53 | 0,025731 |
| BMPR2 | 1,52 | 0,044307 |
| GLG1 | 1,52 | 0,049989 |
| MICAL2 | 1,52 | 0,023687 |
| PPP3CA | 1,52 | 0,029034 |
| RBSN | 1,52 | 0,038627 |
| BPTF | 1,51 | 0,03306 |
| NEDD4L | 1,51 | 0,037268 |
| PUM2 | 1,51 | 0,031876 |
| RBM12 | 1,51 | 0,048281 |
| USP13 | 1,51 | 0,032808 |
| VPS13D | 1,51 | 0,044307 |
| DOCK1 | 1,5 | 0,032698 |
| PDPR | 1,5 | 0,045776 |
