# Supplementary figures and images for: Expression of targets of the RNA-binding protein AUF-1 in human airway epithelium indicates its role in cellular senescence and inflammation
Source: Front Immunol. 2023 Jul 7;14:1192028. doi: 10.3389/fimmu.2023.1192028 (PMC10360199; doi:10.3389/fimmu.2023.1192028)

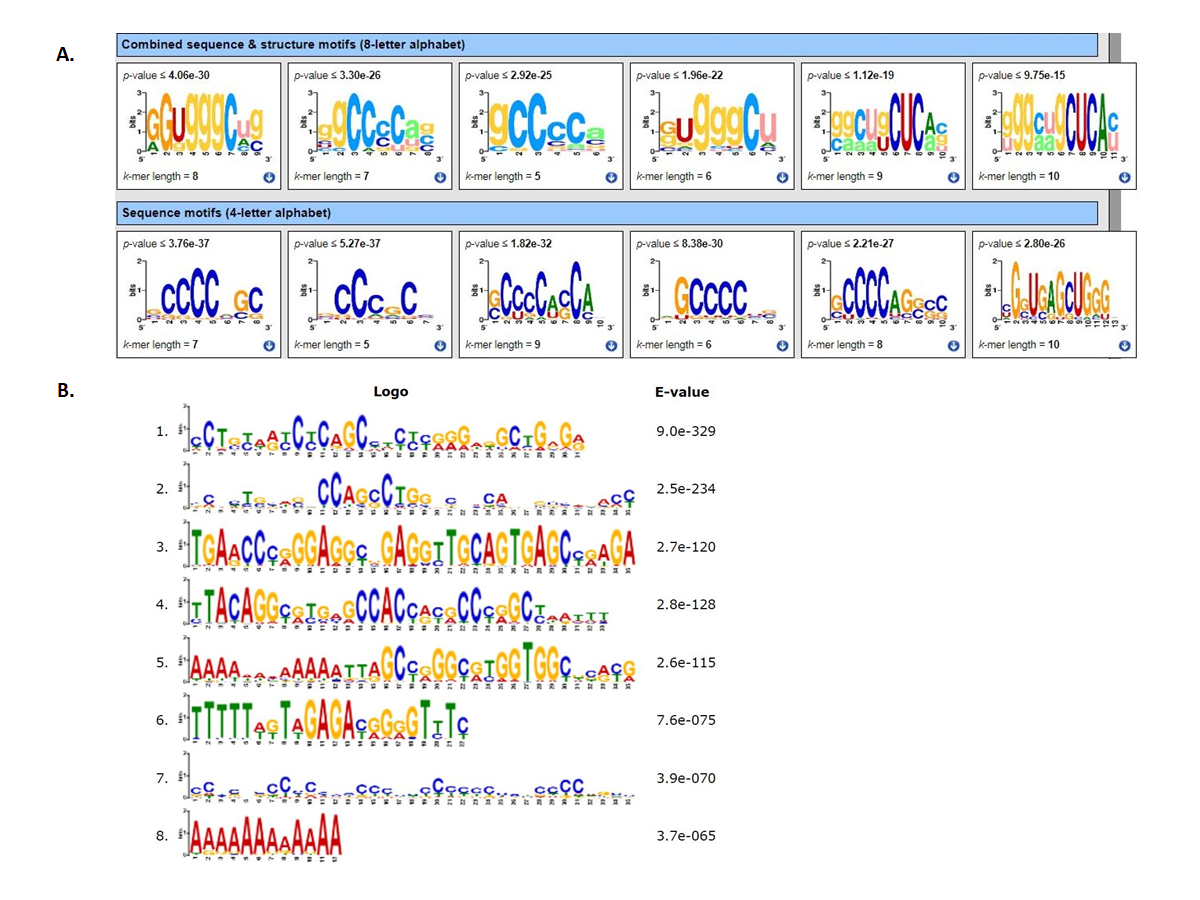

Supplement: Supplementary file 3 [file Image_1.tif]

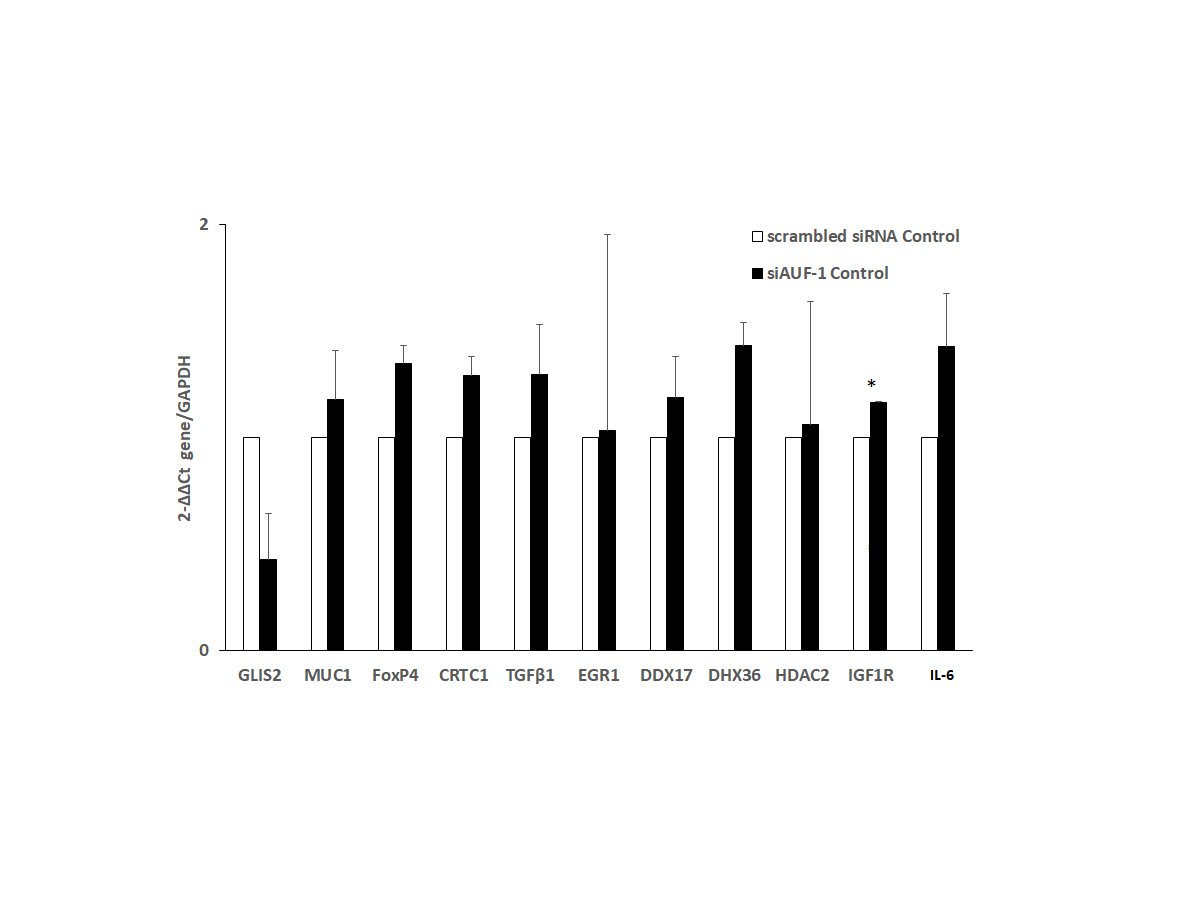

Supplement: Supplementary file 4 [file Image_2.tif]

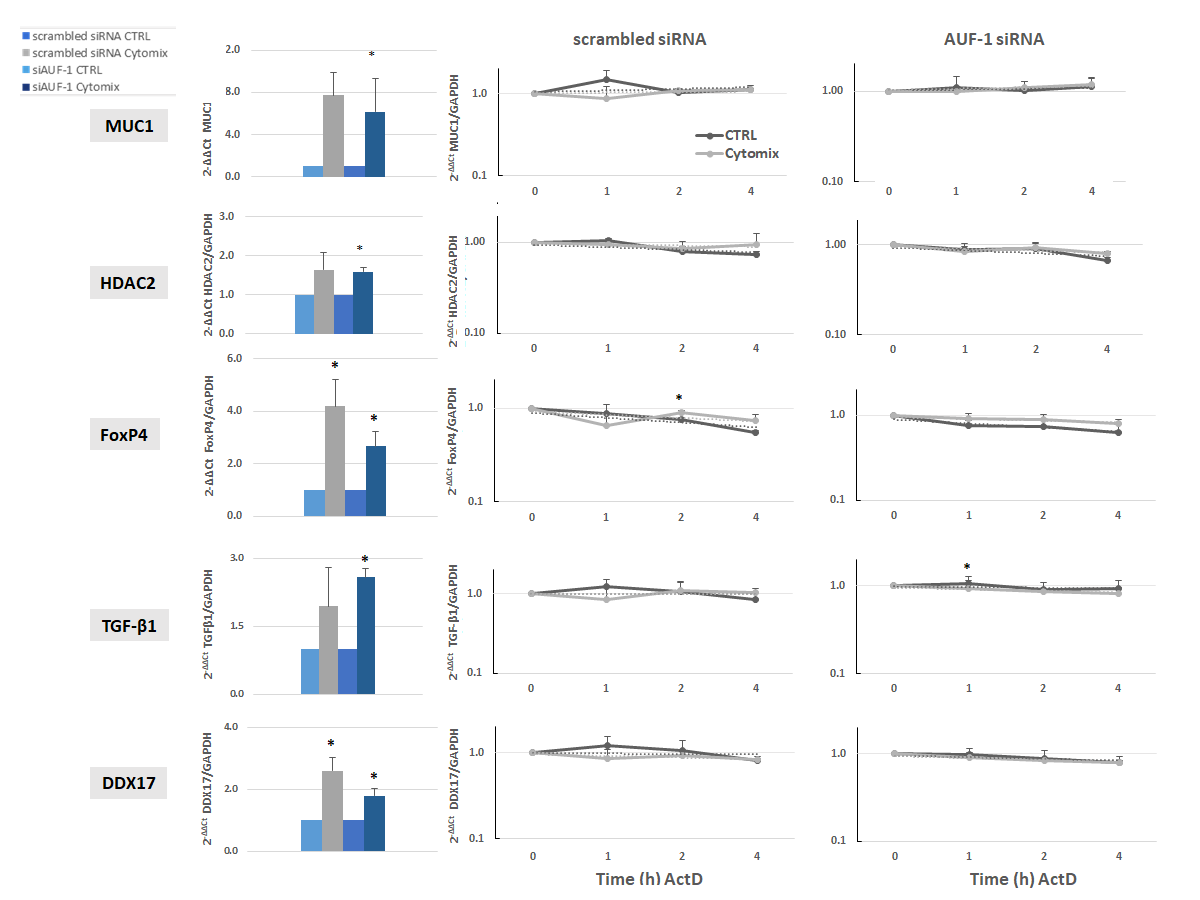

Supplement: Supplementary file 5 [file Image_3.tif]

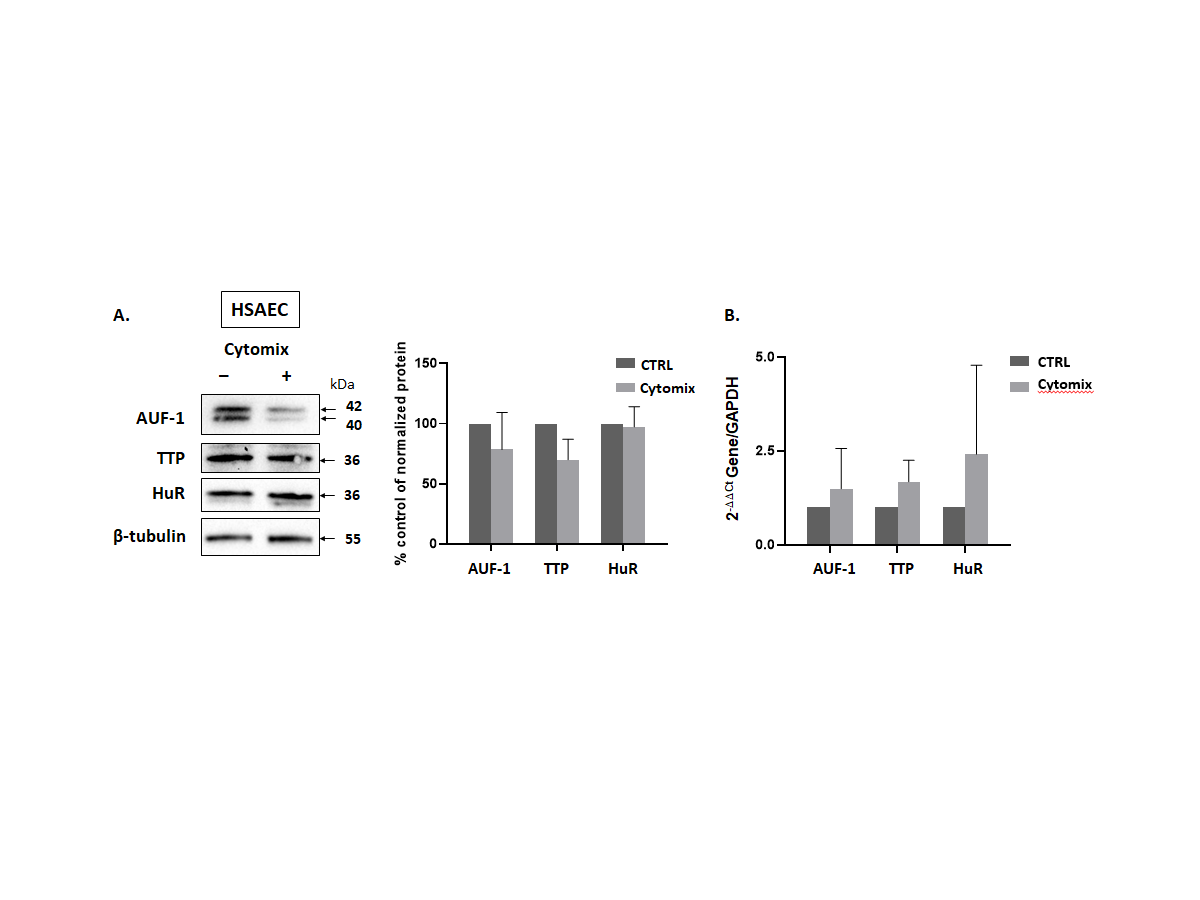

Supplement: Supplementary file 6 [file Image_4.tif]

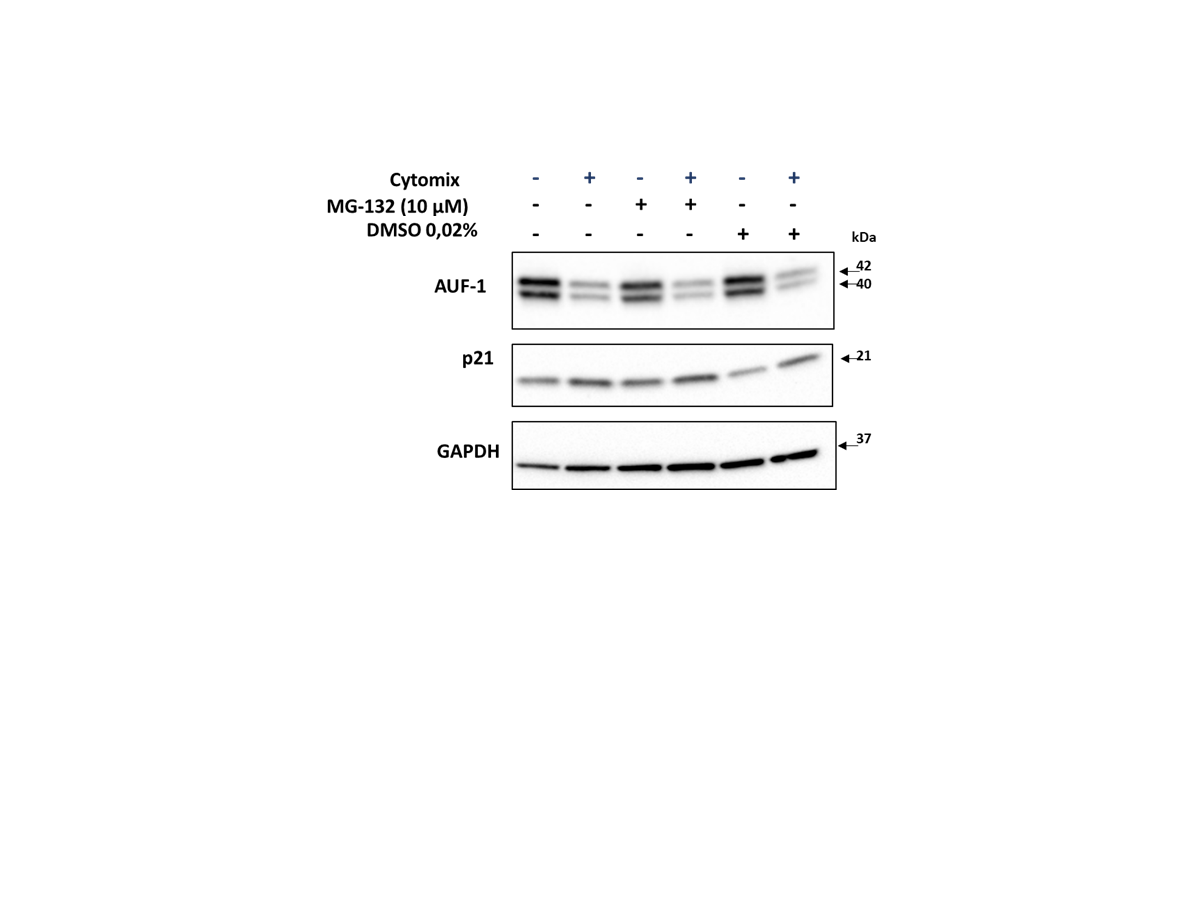

Supplement: Supplementary file 7 [file Image_5.tif]

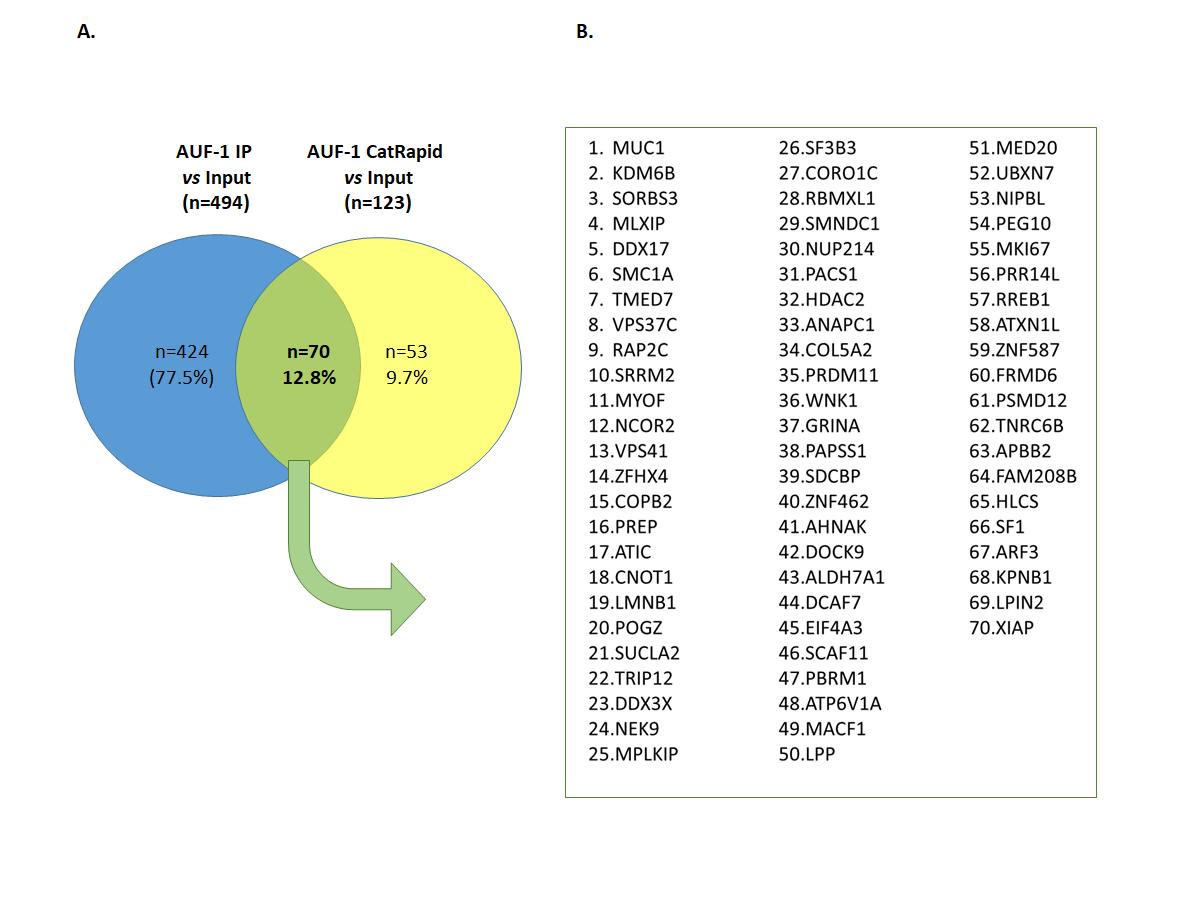

Supplement: Supplementary file 8 [file Image_6.tif]
